# A Randomized, Double-Blind, Placebo-Controlled Assessment of BMS-936558, a Fully Human Monoclonal Antibody to Programmed Death-1 (PD-1), in Patients with Chronic Hepatitis C Virus Infection

**DOI:** 10.1371/journal.pone.0063818

**Published:** 2013-05-22

**Authors:** David Gardiner, Jay Lalezari, Eric Lawitz, Michael DiMicco, Rheem Ghalib, K. Rajender Reddy, Kyong-Mi Chang, Mark Sulkowski, Steven O’ Marro, Jeffrey Anderson, Bing He, Vikram Kansra, Fiona McPhee, Megan Wind-Rotolo, Dennis Grasela, Mark Selby, Alan J. Korman, Israel Lowy

**Affiliations:** 1 Bristol-Myers Squibb, Pennington, New Jersey, United States of America; 2 Quest Clinical Research, San Francisco, California, United States of America; 3 Alamo Medical Research, San Antonio, Texas, United States of America; 4 Advanced Clinical Research Institute, Anaheim, California, United States of America; 5 The Liver Institute at Methodist Hospital, Dallas, Texas, United States of America; 6 University of Pennsylvania School of Medicine, Philadelphia, Pennsylvania, United States of America; 7 Philadelphia Veterans Affairs Medical Center, Philadelphia, Pennsylvania, United States of America; 8 Johns Hopkins University School of Medicine, Baltimore, Maryland, United States of America; 9 Springfield Clinic Infectious Diseases, Springfield, Illinois, United States of America; 10 Bristol-Myers Squibb, Princeton, New Jersey, United States of America; 11 Bristol-Myers Squibb, Wallingford, Connecticut, United States of America; 12 Bristol-Myers Squibb, Milpitas, California, United States of America; 13 Regeneron Pharmaceuticals, Tarrytown, New York, United States of America; University of Sydney, Australia

## Abstract

Expression of the programmed death 1 (PD-1) receptor and its ligands are implicated in the T cell exhaustion phenotype which contributes to the persistence of several chronic viral infections, including human hepatitis C virus (HCV). The antiviral potential of BMS-936558 (MDX-1106) – a fully human anti-PD-1 monoclonal immunoglobulin-G4 that blocks ligand binding – was explored in a proof-of-concept, placebo-controlled single-ascending-dose study in patients (N = 54) with chronic HCV infection. Interferon-alfa treatment-experienced patients (n = 42) were randomized 5∶1 to receive a single infusion of BMS-936558 (0.03, 0.1, 0.3, 1.0, 3.0 mg/kg [n = 5 each] or 10 mg/kg [n = 10]) or of placebo (n = 7). An additional 12 HCV treatment-naïve patients were randomized to receive 10 mg/kg BMS-936558 (n = 10) or placebo (n = 2). Patients were followed for 85 days post-dose. Five patients who received BMS-936558 (0.1 [n = 1] or 10 mg/kg) and one placebo patient achieved the primary study endpoint of a reduction in HCV RNA ≥0.5 log_10_ IU/mL on at least 2 consecutive visits; 3 (10 mg/kg) achieved a >4 log_10_ reduction. Two patients (10 mg/kg) achieved HCV RNA below the lower limit of quantitation (25 IU/mL), one of whom (a prior null-responder) remained RNA-undetectable 1 year post-study. Transient reductions in CD4^+^, CD8^+^ and CD19^+^ cells, including both naïve and memory CD4^+^ and CD8^+^ subsets, were observed at Day 2 without evidence of immune deficit. No clinically relevant changes in immunoglobulin subsets or treatment-related trends in circulating cytokines were noted. BMS-936558 exhibited dose-related exposure increases, with a half-life of 20–24 days. BMS-936558 was mostly well tolerated. One patient (10 mg/kg) experienced an asymptomatic grade 4 ALT elevation coincident with the onset of a 4-log viral load reduction. Six patients exhibited immune-related adverse events of mild-to-moderate intensity, including two cases of hyperthyroidism consistent with autoimmune thyroiditis. Further investigation of PD-1 pathway blockade in chronic viral disease is warranted.

**Trial Registration:**

ClinicalTrials.gov NCT00703469 NCT00703469

## Introduction

Virus-induced suppression of host immunity contributes to the persistence of chronic infections with clinically important viruses including hepatitis C virus (HCV), hepatitis B virus (HBV), and human immunodeficiency virus (HIV) [1]–[3]. Various immunomodulators have been evaluated as therapeutics for these infections, with the goal of overcoming and/or reversing virus-induced immunosuppression. These include interferon-alfa, which is well-established in therapy of HBV and HCV infections, as well as interferon-lambda, toll-like receptor 7 agonists, interleukin-2, interleukin-7, therapeutic vaccines, and other agents [1], [4]–[8]. In the case of HBV and HIV infections, although antiviral therapy provides significant clinical benefits, durable control of the infections with immune modulation remains an unmet goal for many patients.

Multiple mechanisms of viral immune evasion may contribute to viral persistence [3], [9]–[11]. For example, virus interactions with host immune cells can attenuate interferon pathways and cause dysfunction of dendritic cells, macrophages, and natural killer cells [9]. Also, rapid selection of immune escape variants can evade the adaptive immune response. While T cells play a critical role in viral clearance, chronic immune activation resulting from prolonged antigen expression can trigger T cell exhaustion and dysfunction, further contributing to viral persistence [1], [9], [10], [12]. Analysis of T cells in the lymphocytic choriomeningitis virus (LCMV) mouse model of chronic viral infection has demonstrated that the exhausted T cell phenotype is driven, at least in part, by the expression and function of the inhibitory receptor, programmed death 1 (PD-1) [13].

The PD-1 cell surface receptor and its ligands PD-L1 (B7–H1) and PD-L2 (B7–DC) belong to the CD28–B7 family of T-cell regulatory pathways with a critical role in maintaining the balance between protective immunity against foreign pathogens and destructive autoimmunity [14]–[16]. PD-1 is induced upon activation on various immune cell subsets, including CD4^+^ and CD8^+^ T cells, natural killer cells, B cells, monocytes and some dendritic cells. PD-L1 is expressed on multiple lymphoid and peripheral cell types and is induced by inflammatory cytokines commonly associated with viral infection, such as IFN-gamma. Expression of PD-L2 is more restricted to myeloid cells, including dendritic cells [16], [17]. Engagement of PD-1 by either of its ligands globally reduces T cell activity through the inhibition of cytokine production, cytolytic function and T-cell proliferation [13]. PD-1/PD-L1 interactions also contribute to T regulatory function and development [18], [19], and data demonstrate that the PD-1 pathway is a major mechanism utilized by human tumors to evade immune responses [20], [21]. Several solid tumors have been shown to over-express the ligands for PD-1, PD-L1 and PD-L2, allowing these tumors to directly suppress T-cells activated by tumor-specific antigens [22]–[24]. This understanding of the function of the PD-1/PD-L1 interaction in tumor immune evasion has led to several approaches to restore immune response to tumors by suppression of the PD-1 pathway.

The PD-1 pathway has been implicated in T-cell exhaustion associated with chronic viral infections in humans, including HCV infection [12], [14]. Persistent viremia has been associated with upregulation of PD-1 expression on virus-specific CD8+ T-cells [25]–[28]. In patients with chronic HIV, HBV or HCV infections, enhanced T cell expression of PD-1 has been associated with T-cell exhaustion, manifested by reduced virus-specific proliferative capacity and cytokine expression [25], [26], [29], [30]. Importantly, CD8^+^ cell function could be partially restored *ex vivo* by blockade of the PD-1 pathway [25], [26], [29], [31]–[34]. Consistent with these findings, treatment of simian immunodeficiency virus (SIV)-infected macaques with anti-PD-1 antibodies resulted not only in rapid expansion of SIV-specific CD8+ T-cells with improved functional characteristics *in vivo*, but also an increase in anti-SIV humoral immunity as well as increased survival [35], [36]. Similarly, in vivo blockade of the PD-1 pathway resulted in enhanced antiviral effector T cell responses and control of murine LCMV infection [13]. Thus, the PD-1 pathway is a target with potentially broad application for therapy of multiple chronic viral infections. In addition, other inhibitory pathways have been implicated in suppression of immune responses against chronic viral infections, including cytotoxic T lymphocyte-associated antigen 4 (CTLA-4), T cell immunoglobulin 3 (Tim-3) and lymphocyte activation gene-3 (Lag-3) [2], [12], [37], [38].

BMS-936558 (MDX-1106) is a fully human monoclonal immunoglobulin G4 (IgG4 [S228P]) that targets PD-1 and inhibits its binding to PD-L1 and PD-L2. BMS-936558 monotherapy, when dosed continuously on a Q2W schedule, has demonstrated significant and durable responses in pretreated patients with advanced non-small cell lung cancer, melanoma and renal cell carcinoma, with generally good tolerability [21], [39], [40]. These early results have led to the initiation of Phase III registrational trials across each indication (NCT01668784, NCT01642004, and NCT01673867). The study described herein is the first clinical evaluation of BMS-936558 in patients with chronic HCV infection. The study was undertaken as a Proof-of-Concept to determine whether exploiting PD-1 blockade has potential as a therapy for chronic viral infections in which PD-1 upregulation contributes to viral immune escape and persistence.

## Materials and Methods

The protocol for this trial and supporting CONSORT checklist are available as supporting information; see Protocol S1 and Checklist S1, respectively.

### Ethics Statement

Written informed consent was obtained from all patients. The study was approved by five institutional review boards (IRBs) responsible for the seven study sites: the Western IRB (Baltimore, MD; 3 sites); Johns Hopkins Medicine IRB (Baltimore, MD); the University of Pennsylvania Office of Regulatory Affairs (Philadelphia, PA); Aspire IRB (La Mesa, CA), and the Fox Commercial IRB Ltd (Springfield, IL). The study was conducted in compliance with the Declaration of Helsinki, Good Clinical Practice Guidelines, and local regulatory requirements.

### Study Design and Patients

This was a randomized, modified double-blind (see below), placebo-controlled single ascending dose study (clinicaltrials.gov identifier NCT00703469) evaluating the safety, pharmacokinetics and immunogenicity of BMS-936558 in HCV-infected patients enrolled at seven US centers (Dallas and San Antonio, Texas; San Francisco and Anaheim, California; Baltimore, Maryland; Philadelphia, Pennsylvania, and Springfield, Illinois) between October 2008 and July 2009.

Eligible patients were adult (≥ 18 years) men and women with chronic HCV genotype 1 infection of at least 6 months duration and screening serum HCV RNA ≥100,000 IU/mL. Both HCV treatment-naïve patients and patients who failed prior interferon-alfa-based therapy (treatment-experienced) were included. Treatment experience was defined as lack of sustained virologic response after at least 12 weeks of interferon-alfa or pegylated interferon-alfa combined with ribavirin. Patients were asymptomatic or with only minor HCV symptoms not restricting normal activities. Liver biopsy results consistent with chronic HCV infection without evidence or history of bridging fibrosis or cirrhosis, were required within 2 years prior to study entry. Eligible patients had adequate bone marrow, liver, and renal function (creatinine <1.5×ULN), as well as normal blood pressure, electrocardiograms, chest x-rays, and alfa-fetoprotein levels. Females of child-bearing potential and all males were required to use adequate contraception for 70 days (females) or 180 days (males) after study drug administration. Patients coinfected with human immunodeficiency virus, hepatitis B virus, or with other active infections (e.g. respiratory/urinary tract, herpes simplex) were excluded; as were patients using immunosuppressive, antiviral, or interferon-based therapies within 28 days of study drug administration, or with a history or evidence of autoimmune or immunodeficiency disease, cancer, bleeding disorders, alcohol or drug misuse, or any serious medical condition other than hepatitis C.

In the first part of the study, treatment-experienced patients were assigned to one of six escalating dose cohorts (0.03, 0.1, 0.3, 1.0, 3.0, or 10 mg/kg BMS-936558), with 6 patients per cohort. Within each cohort, patients were randomly assigned in a 5∶1 ratio to receive a single dose of BMS-936558 or placebo by intravenous infusion administered over 15 minutes (0.03 mg/kg), 30 minutes (0.1 mg/kg) or 60 minutes (all other doses). Patients were observed for infusion reactions at their study sites for 6 hours post-infusion.

Study visits consisted of a screening visit (Day −28 to −6), a pre-treatment visit (Day −5 to −2) and Days 1 (dosing), 2, 3, 8, 15, 22, 29, 31 (delayed-type hypersensitivity skin testing only), 43, 57 and 85. Randomization was by computer-generated tables provided by the sponsor. The sponsor notified the site pharmacist (who prepared the infusion) of the treatment assignment. All other site staff remained blinded to treatment assignments. The sponsor was not blinded. Randomized patients were assigned to the next available dose cohort following a safety assessment and agreement to escalate among the investigators and the sponsor’s medical monitor.

The study began with the lowest dose (0.03 mg/kg). Each subsequent cohort was initiated when all patients in the previous cohort reached Day 29 without dose-limiting toxicities (DLTs; adverse events or laboratory abnormalities of at least grade 3, considered at least potentially related to study drug and reported within 28 days of dosing). Dose escalations continued until the last cohort was enrolled or the maximum tolerated dose (MTD) was identified, defined as the highest dose without a DLT. After completion of dose escalation in treatment-experienced patients, treatment-naïve and additional treatment-experienced patients were enrolled at the highest dose achieved (10 mg/kg or the MTD) and randomized as described.

All patients were followed for 85 days after dosing. Patients with HCV RNA declines >2 log_10_ IU/mL were followed for an additional 12 months after completion of the protocol in a separate, long-term follow-up study.

### Endpoints and Assessments

The primary endpoint for pharmacologic activity was the proportion of subjects in each dosing cohort who achieved clinical response, defined as a decline in HCV RNA from baseline of at least 0.5 log_10_ IU/mL in 2 or more consecutive measurements (COBAS TaqMan HCV test, Roche Diagnostics, Pleasanton, CA; lower limit of quantitation of 25 IU/mL and a limit of detection of approximately 10 IU/mL). Secondary pharmacodynamics endpoints included the magnitude and duration of HCV RNA changes, changes in levels of serum cytokines, and clinical immunology assessments (PBMC reactivity to HCV antigens, type IV hypersensitivity response to *Candida albicans*/tetanus toxoid antigen skin test, and anti-tetanus antibody titers). Safety assessments included treatment-emergent adverse events and laboratory abnormalities, physical examinations, immunogenicity (human anti-human antibody), treatment-associated cytokine changes, and changes in immune cell subsets by flow cytometry. Peripheral blood mononuclear cells (PBMCs) were collected throughout the study and stored for analysis of antigen-specific T-cell responses via ELISpot using HCV-specific peptide pools. IL28B genotype (rs12979860 single nucleotide polymorphism) was assessed as part of the follow-up study in 3 individuals with >4 log_10_ IU/mL reductions in HCV RNA, and classified as homozygous CC, heterozygous CT or homozygous TT by a TaqMan genotyping assay (Applied Biosystems, Foster City, CA).

Rebound viremia in subjects who experienced transient declines in HCV RNA was assessed for potential superinfection events by examination of the HCV NS5A gene region. HCV RNA was extracted from plasma samples obtained at baseline and at various postinfusion time points, and NS5A amplicons generated by RT-PCR were sequenced. Resulting population sequences were subjected to a Basic Local Alignment Search Tool (BLAST) search against NS5A genotype 1a samples in the BMS clinical database and the Los Alamos National Laboratory (LANL) HCV database. A neighbor-joining tree was created based on the alignment using the Molecular Evolutionary Genetics Analysis (MEGA) version 5.05 software package (http://www.megasoftware.net) [41] with a bootstrap test of 500 replicates. The tree was rooted using a genotype 1b sequence.

HCV genotyping and protocol-defined laboratory safety assessments including cytokines and flow cytometric analyses were performed by Covance Central Laboratory Services, Inc. (Indianapolis, IN, USA). Flow cytometry for determination of immune cell cluster of differentiation (CD) antigens and HLA-DR was performed by Covance Inc. using fluorescently labeled monoclonal antibodies from Beckton Dickinson (BD Biosciences, San Jose, CA, USA). HCV genotype was determined by Trugene HCV genotyping assay (Siemens Healthcare Diagnostics, Tarrytown, NY).

A quantitative enzyme-linked immunosorbent assay (ELISA) was used for pharmacokinetic assessments of BMS-936558. Recombinant PD-1/Fc chimera (R&D Systems, Minneapolis, MN) was adsorbed onto a microtiter plate to capture BMS-936558 contained in serum samples. Captured BMS-936558 was detected using a commercially obtained, purified goat anti-human antibody labeled with alkaline phosphatase, with a *p*-nitrophenyl phosphate substrate for colorimetric readout, and compared to a standard curve for quantitation. Serum concentrations of BMS-936558 were assessed on Day 1 pre-infusion and at 1, 1.25, 1.5, 2, 3, 4, 6, 24, and 48 hours after start of infusion. Additional samples were taken at 0.25, 0.5, and 0.75 hours after start of infusion for patients receiving 0.03 mg/kg, and at 0.5 and 0.75 hours after start of infusion for subjects receiving 0.1 mg/kg. Single serum samples for pharmacokinetic parameters were taken on Days 8, 15, 22, 29, 43, 57, and 85 for all patients. Serum concentration versus time data were analyzed by non-compartmental methods using the program Kinetica (Thermo Scientific, Philadelphia, PA, USA). Actual sampling times were used for PK parameter calculations.

### Statistical Methods

The study employed an initial dose escalation design of 5 patients dosed with drug and 1 with placebo dosed at each level, and a single occurrence of a set of predefined DLTs was deemed unacceptable. Based upon binomial probability, this design had an approximately 77% likelihood of observing no DLTs at a dose level if the true rate of DLT occurrence was 5%, and a 76% probability of observing at least one DLT if the true rate of DLT occurrence was 25%. Additional patients were enrolled following determination of the maximum tolerated dose. Analysis of antiviral activity, immunobiologic findings and safety were conducted on a modified intent-to-treat population (ITT-exposed), comprising all patients who received at least a partial dose of study drug. Descriptive statistics were used to summarize all antiviral activity, safety, immunobiologic and pharmacokinetic data.

## Results

### Patient Disposition and Baseline Characteristics

**Figure 1:**
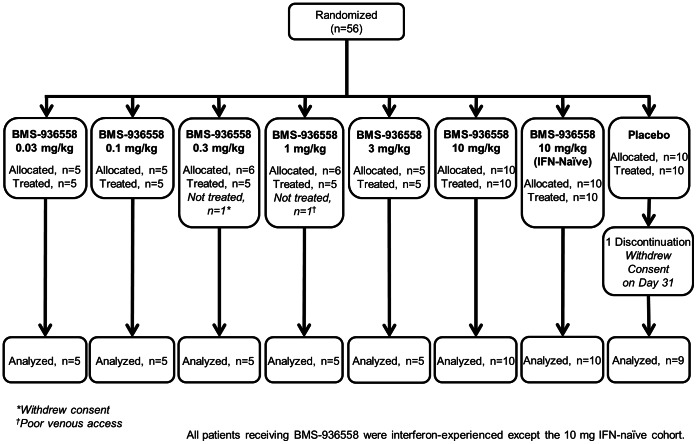
Patient disposition.

A total of 56 patients were randomized, of whom 54 were treated between August 8, 2008 and July 27, 2009 (45 BMS-936558, 9 placebo). Patient disposition is shown in Figure 1. All treated subjects in the ITT-exposed population completed the Day 29 evaluations. One patient assigned to placebo withdrew consent at Day 31 and discontinued the study. No patient discontinued due to an adverse event.

There were no clinically relevant differences between dose groups in baseline disease or demographic parameters (Table 1). Most patients were white males with 57% (31/54) HCV genotype 1a infections overall; treatment-naïve patients were somewhat younger than other treatment groups. Ten subjects who received 10 mg/kg of BMS-936558 and 2 placebo subjects were treatment-naive. Among treatment-experienced patients, approximately 38% (16/42) had prior null response to peginterferon-alfa/ribavirin therapy, 12% (5/42) had partial response, 36% (15/42) had post-treatment relapse, and 14% (6/42) had received incomplete or inadequate therapy.

**Table 1:** Baseline characteristics.

		BMS-936558 (mg/kg)							Placebo
		0.03	0.1	0.3	1	3	10	10 (Naïve)	
N		5	5	5	5	5	10	10	9
Age, mean (SD) years		55.8 (7.3)	51.6 (3.4)	50.8 (5.4)	48.2 (10.4)	52.0 (10.1)	53.5 (5.8)	44.3 (9.9)	47.1 (4.4)
Sex, n (%) male		2 (40)	2 (40)	2 (40)	3 (60)	3 (60)	6 (60)	9 (90)	7 (77.8)
Weight, mean (SD) kg		88.9 (12.9)	84.0 (10.7)	83.2 (16.2)	78.0 (21.7)	80.1 (26.7)	75.6 (11.7)	85.0 (14.3)	79.8 (8.8)
Race, n (%)	White	4 (80)	3 (60)	4 (80)	5 (100)	4 (80)	8 (80)	8 (80)	6 (66.7)
	Black	1 (20)	2 (40)	1 (20)	0	1 (20)	2 (20)	1 (10)	3 (33.3)
	Other	0	0	0	0	0	0	1 (10)	0
Time since diagnosis, mean (SD) years		12.7 (6.6)	5.5 (3.1)	7.3 (2.3)	8.2 (5.0)	6.8 (2.9)	10.9 (6.9)	7.3 (5.3)	9.6 (8.1)
HCV RNA, mean (SD) log_10_ IU/mL		6.63 (0.21)	6.42 (0.47)	6.45 (0.38)	6.48 (0.45)	6.42 (0.51)	6.31 (0.56)	6.41 (0.70)	6.42 (0.65)
HCV genotype, n (%)	1a	3 (60)	3 (60)	3 (60)	3 (60)	3 (60)	4 (40)	5 (50)	7 (77.8)
	1b	2 (40)	2 (40)	2 (40)	1 (20)	1 (20)	4 (40)	2 (20)	1 (11.1)
	1 (unsubtyped)	0	0	0	1 (20)	0	2 (20)	3 (30)	1 (11.1)
	1b/2b	0	0	0	0	1 (20)	0	0	0
Treatment experience	Naive	0	0	0	0	0	0	10	2
	Null response	4	1	0	2	1	4	0	4
	Partial response	0	1	0	0	1	1	0	2
	Relapse	1	1	2	3	3	4	0	1
	Treatment incomplete or inadequate	0	2	3	0	0	1	0	0

### Antiviral Outcomes

In the ITT-exposed population, clinical response (HCV RNA decline ≥0.5 log_10_ IU/mL on at least two consecutive visits) was observed in six patients: one of five (20%) treatment-experienced patients who received BMS-936558 0.1 mg/kg, one of 10 (10%) in the treatment-experienced group receiving BMS-936558 10 mg/kg, three of 10 (30%) in the treatment-naïve group receiving BMS-936558 10 mg/kg, and one of nine (11%) placebo recipients (Table 2). The duration of response was ≥8 weeks for four of the six responders and between 4 and 8 weeks in two.

**Table 2:** Antiviral activity results.

	BMS-936558 (mg/kg)									Placebo
		0.03	0.1	0.3	1	3	10	10 (Naïve)	Total	
N		5	5	5	5	5	10	10	45	9
Clinical response^a^	n (%)	0	1 (20.0)	0	0	0	1 (10.0)	3 (30.0)	5 (11.1)	1 (11.1)
	95% CI	ND	ND	ND	ND	ND	0.3, 44.5	6.7, 65.2	ND	ND
Duration of response(weeks), n (%)	≥4 to <8	0	1 (20.0)	0	0	0	0	1 (10.0)	2 (4.4)	0
	≥8	0	0	0	0	0	1 (10.0)	2 (20.0)	3 (6.7)	1 (11.1)
Max. log_10_ HCV RNAdecrease from BL, n (%)	<0.5	5 (100.0)	4 (80.0)	5 (100.0)	5 (100.0)	5 (100.0)	9 (90.0)	7 (70.0)	40 (88.9)	8 (88.9)
	≥0.5 to <1.0	0	1 (20.0)	0	0	0	0	1 (10.0)	2 (4.4)	1 (11.1)
	≥2	0	0	0	0	0	1 (10.0)	2 (20.0)	3 (6.7)	0

BL, baseline; 95% CI, 95% confidence interval.

a Defined as ≥0.5 log decline from baseline in HCV RNA on at least 2 consecutive measures.

**Figure 2:**
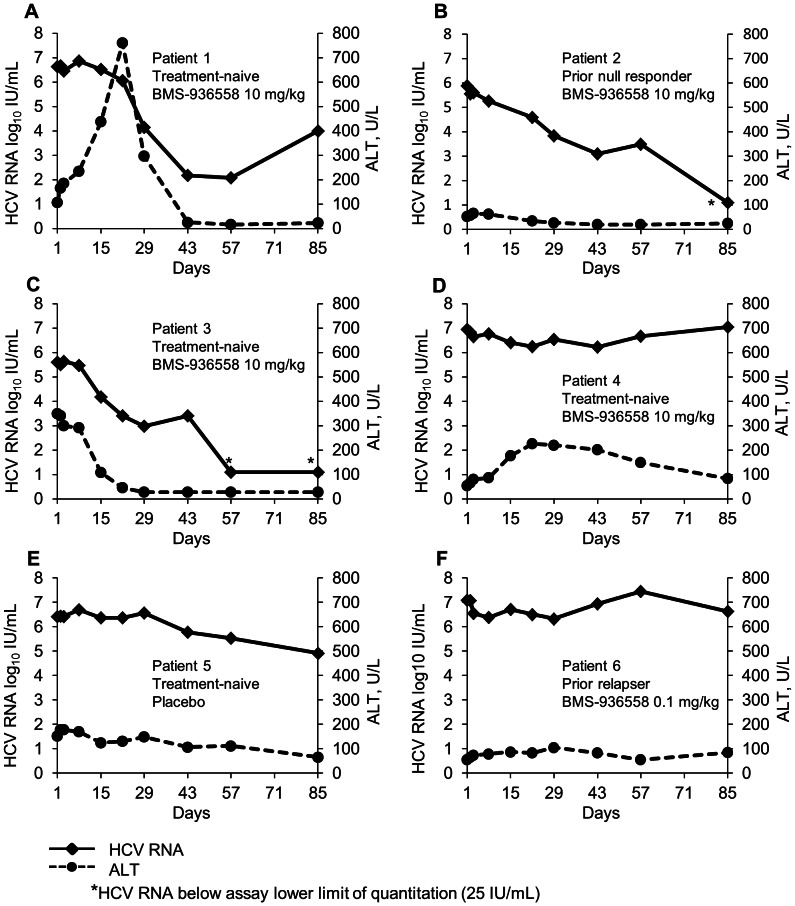
HCV RNA and ALT changes in patients with clinical response.

Among the six clinical responders, three patients receiving BMS-936558 10 mg/kg – two treatment-naïve and one prior null responder – experienced reductions in serum HCV RNA that exceeded 4 log_10_ IU/mL at nadir (Figure 2 and Table 2). Of these three, one *IL28B*-CC homozygous, treatment-naive patient with HCV GT-1a from a pre-study genotype assay (patient 1; Fig. 2A) experienced a 4.55 log_10_ decline in HCV RNA which subsequently began to return to pre-treatment levels on-study. One prior null responder with HCV genotype 1a from pre-study assay data showed HCV RNA that dropped below the assay lower limit of quantitation at study Day 85 (Patient 2; Fig. 2B), subsequently became undetectable, and remained undetectable for more than 1 year after study completion. This patient was an *IL28B*-TT homozygote, consistent with prior non-response to interferon-alfa treatment [42], [43]. Finally, one treatment-naïve, *IL28B*-CC patient demonstrated HCV RNA below the assay lower limit of quantitation at Study days 57 through 85 (patient 3; Fig. 2C). In post-study follow up this patient became transiently undetectable before HCV RNA returned to pretreatment levels. This patient was infected with HCV genotype 1 but his subtype could not be established. In addition, in this latter patient, population genotypic analysis of the NS5A region from baseline and relapse samples confirmed that re-infection had not occurred (data not shown). All three patients were male, and all received BMS-936558 in July 2009. There were no other homogeneous factors or features among them.

### Immunologic Outcomes

No clinically relevant changes in immunoglobulin subsets (IgM, IgA, IgG) were observed. Mean changes from baseline were <15% in both BMS-936558 and placebo groups for all three subsets during follow-up.

**Figure 3:**
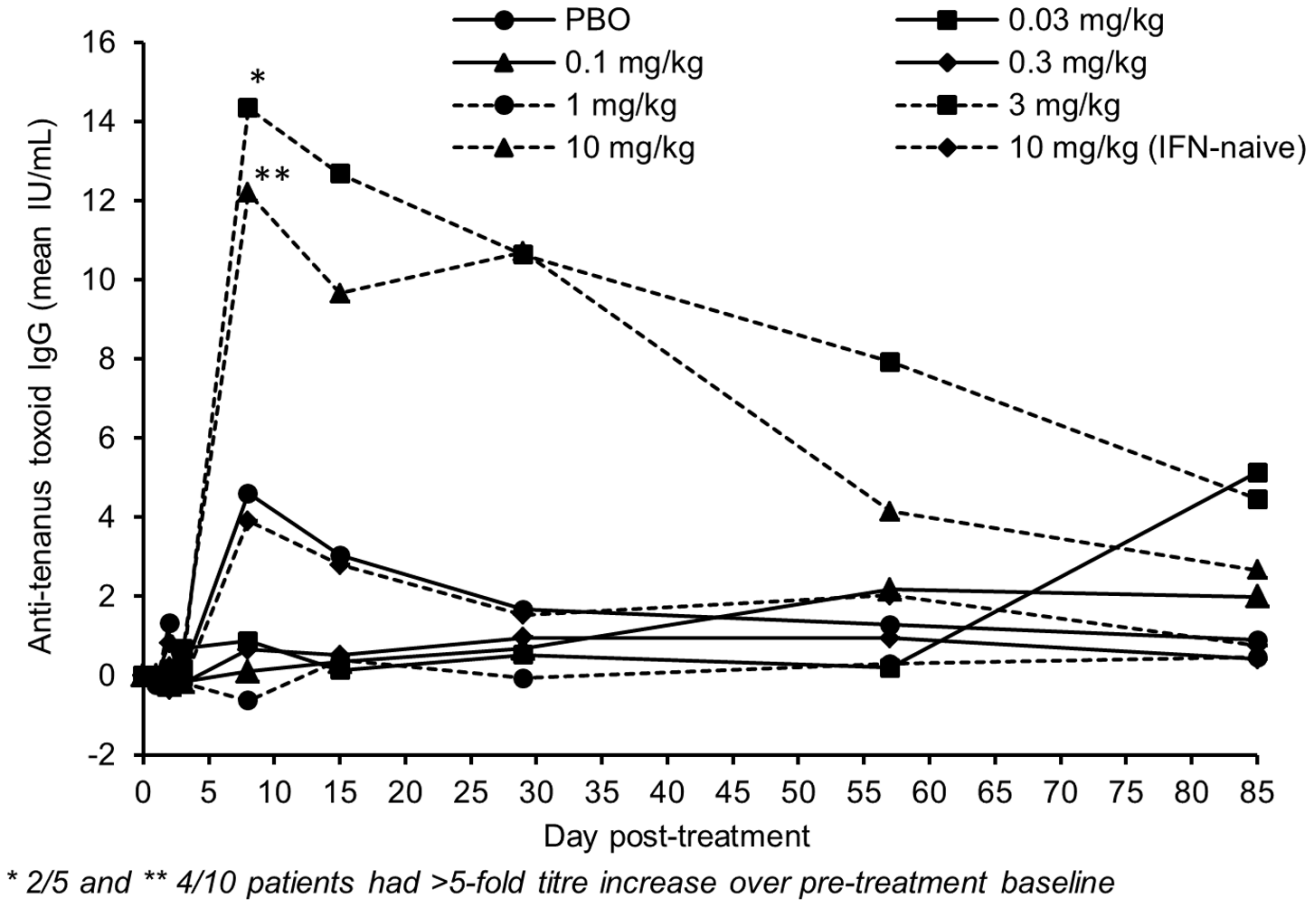
Mean changes from baseline in anti-tetanus antibody titer.

The immunoadjuvant activity of BMS-936558 on antibody titers to tetanus toxoid was explored. Anti-tetanus antibody levels varied between patients but median levels were generally comparable for those who received study drug (any dose) versus placebo. Some patients in the placebo group showed transient increases in tetanus titers at Day 8, while subjects at dose levels of 0.03 through 1 mg/kg showed only minimal changes throughout the study. In contrast, greater than 5-fold increases in anti-tetanus antibody titer over pre-treatment baseline were seen at Day 8 in two patients who received 3.0 mg/kg BMS-936558 (9-fold and 11-fold over baseline) and in four treatment-experienced patients who received 10 mg/kg (6-fold to 36-fold over baseline). These increases subsequently declined back towards baseline (Figure 3).

In quantitative type IV hypersensitivity skin tests, there were no treatment-related patterns in *Candida-*specific or tetanus-specific erythema or induration.

Four patients had positive responses in BMS-936558 immunogenicity assessments (human-anti-human antibody [HAHA] analysis) conducted pretreatment, Day 29 and Day 85. One patient was positive pretreatment but negative thereafter, two patients were positive only on Day 85, and one patient was positive on Days 29 and 85. The latter three patients received low doses (0.03 or 0.3 mg/kg) of BMS-936558.

There were no apparent treatment-related trends in mean serum levels of IL-13, IP-10, neopterin, TGF-β1, IFN-γ, IL-10, IL-12, IL-1B, IL-2, IL-4, IL-5, IL-6, IL-8, or TNF-α during follow-up (data not shown).

Mean absolute values and percentages of CD3^+^, CD4^+^, CD8^+^, CD19^+^, CD3^+^/CD4^+^ (± CD38^+^, ± HLA-DR^+^), CD3^+^/CD8^+^ (± CD38^+^, ± HLA-DR^+^), CD4^+^/CD25^+^, CD16^+^/CD56^+^, and in the CD4^+^/CD8^+^ and CD3^+^/CD19^+^ ratios on peripheral blood lymphocytes, were examined by flow cytometry. Treatment was associated with substantial but transient declines at Day 2 in absolute CD4^+^ and CD8^+^ cells (both the memory and naïve subsets) and in CD19^+^ cells (Table 3) that were not dose-related. Substantial changes in cell percentages were not evident (Table 3). Cell counts generally returned to pretreatment levels after one week. PBMCs were obtained for ELISpot analysis of antigen-specific T-cell responses pre- and post-treatment. Unfortunately, cell viability was very low (generally <30% viable cells) and not permissive of ELISpot analysis.

**Table 3:** Immune cell changes from baseline at Day 2.

		Mean (SD) change from baseline to Day 2			
		Absolute cells/µL		Percentage cells	
		All BMS-936558 (N = 45)	Placebo (N = 9)	All BMS-936558 (N = 45)	Placebo (N = 9)
CD4^+^	Overall	−269 (179)	+41 (228)	−3.0 (3.5)	+1.0 (4.0)
	CD45RA^+^/RO^–^ (naïve)	−49 (64)	−3 (31)	–	–
	CD45RO^+^/RA^–^ (memory)	−207 (156)	−3 (220)	–	–
CD8^+^	Overall	−124 (98)	−21 (105)	0 (2.4)	−1.0 (2.0)
	CD45RA^+^/RO^–^ (naïve)	−44 (51)	−13 (52)	–	–
	CD45RO^+^/RA^–^ (memory)	−60 (51)	−10 (39)	–	–
CD19^+^		−74 (69)	−13 (73)	+0.4 (2.9)	−0.2 (2.2)

### Safety

The most common adverse events were fatigue, headache, diarrhea, and pharyngeal pain (Table 4), with no pattern suggesting dose-related differences in frequencies. All events were mild or moderate in intensity (grade 1 or 2) with the exception of one severe (grade 4) ALT elevation in a treatment-naïve patient who received BMS-936558 10 mg/kg. Infection-related adverse events suggested neither treatment-related immune deficits nor exaggerated immune responses during the observation period. The grade 4 ALT elevation occurred in a 51 year-old male diagnosed with hepatitis C in 2006 and naive to prior therapy. ALT was normal at baseline but began increasing two days after dosing with BMS-936558 10 mg/kg (Figure 2A). ALT levels peaked on Day 22 at 17-fold above the upper limit of normal (43 U/L), concurrent with the maximum reduction in HCV RNA (4.55 log_10_ IU/mL). Other indicators of hepatic function, such as bilirubin levels and INR, remained stable. Serologic evaluation for concurrent liver disease was unrevealing. This event resolved without intervention.

**Table 4:** Adverse events reported in ≥5% BMS-936558 (combined) or placebo recipients.

	BMS-936558 (mg/kg)								Placebo
	0.03	0.1	0.3	1	3	10	10 (Naïve)	Total	
N	5	5	5	5	5	10	10	45	9
Any AE, n (%)	3 (60)	4 (80)	4 (80)	4 (80)	4 (80)	6 (60)	10 (100)	35 (78)	6 (67)
Fatigue, n (%)	0	2 (40)	0	0	2 (40)	2 (20)	3 (30)	9 (20)	1 (11)
Headache, n (%)	0	3 (60)	1 (20)	1 (20)	0	1 (10)	2 (20)	8 (18)	1 (11)
Diarrhea, n (%)	0	2 (40)	0	2 (40)	2 (40)	0	0	6 (13)	1 (11)
Pharyngeal pain, n (%)	0	1 (20)	0	1 (20)	1 (20)	0	2 (20)	5 (11)	1 (11)
Cough, n (%)	0	1 (20)	0	2 (40)	1 (20)	0	0	4 (9)	1 (11)
Upper abdominal pain, n (%)	0	1 (20)	0	0	2 (40)	0	0	3 (7)	0

Immune-related adverse events, defined as clinically significant events consistent with an immune-mediated mechanism and of otherwise unknown etiology, were identified in six BMS-936558 recipients. These events included one patient who experienced hyperthyroidism, hypothyroidism, urticaria and pruritus; one patient who experienced hyperthyroidism and diarrhea; two patients with diarrhea alone, and one patient each with blister and rash, respectively. All events were mild to moderate in intensity and resolved without specific intervention. Hypo- and hyperthyroidism were established by assay of TSH, total T3 and free T4.

In addition to the grade 4 ALT elevation previously described, two other potentially immune-related events are of particular note: one case of hyperthyroidism in a patient on concomitant levothyroxine that required medical intervention, and one exacerbation of adult onset diabetes.

The hyperthyroidism event concerned an interferon-alfa-experienced 52 year-old male diagnosed with HCV infection in 1995 and hypothyroidism in 1997, requiring levothyroxine administration prior to study entry. The patient’s baseline TSH (1.96 MIU/L) and total T3 (2.2 nmol/L) were within the normal ranges of 0.34–5.6 MIU/L and 1.2–2.3 nmol/L, respectively. The patient received 1 mg/kg of BMS-936558 and subsequently reported insomnia on Day 5, diarrhea and increased appetite on Day 13, and anxiety on Day 29. Thyroid function tests on Day 29 revealed low TSH (0.14 MIU/L) and mildly elevated total T3 (2.7 nmol/L); the subject was diagnosed with hyperthyroidism and instructed to discontinue levothyroxine. Antithyroglobulin antibody (ATGA) titer was slightly high at pre-treatment baseline (17.0 IU/mL [normal range 0.0–14.4], increased to 264 IU/mL at Day 29, and subsequently increased further to a maximum of 397 IU/mL on Day 57. Symptoms improved; TSH measured 26.28 MIU/L on Day 57 and the patient was restarted on levothyroxine 25µg daily. TSH and ATGA remained elevated through the end of the study and the subject remained on treatment for hypothyroidism.

The exacerbation of diabetes concerned a 45 year old Caucasian male with HCV infection since 2000 and ongoing diabetes treated with metformin, who experienced significant worsening of blood glucose control on study day 22 requiring the introduction of insulin which persisted until study conclusion. Computer-assisted tomography did not reveal a structural cause for the changes and no coincident infection or other precipitating cause of the exacerbation of his diabetes was identified. Anti-insulin antibodies and other markers of autoimmune diabetes were not examined [44].

### Pharmacokinetics

BMS-936558 demonstrated dose-related increases in exposure across the range of doses studied, with near dose-proportional increases over the dose range of 1 to 10 mg/kg (Table 5). Specifically, for an increase in dose in the ratio of 1.0∶ 3.0∶ 10, there was an increase in C_max_ in a ratio of 1.0∶ 3.0∶ 7.3 and an increase in AUC_INF_ of 1.0∶ 3.4∶ 9.5. Furthermore, BMS-936558 exhibited a long serum half-life (T_½_), of 20.6 days for the 1 mg/kg cohort, increasing slightly to 23.7 days for the 10 mg/kg cohort. The variability of derived exposure measures C_max_ and AUC_INF_ was consistently modest (coefficients of variation between 13% and 20%) at doses from 1 to 10 mg/kg, but higher between 0.03 and 0.3 mg/kg, most likely due to assay limitations for quantifying the low serum levels obtained at the lower doses.

**Table 5:** Summary of pharmacokinetic parameters.

BMS-936558Dose (mg/kg)	N	C_max_ (µg/mL)g. mean (% CV)	T_max_ (hours) median (min-max)	AUC_0-T_ (µg·h/mL)g. mean (% CV)	AUC_INF_ (µg·h/mL)g. mean (% CV)	T_½_ (days) mean (SD)
0.03^a^	3	1.41 (17)	3.25 (1.8–6.3)	4.20 (151)	NR	NR
0.1	5	3.52 (129)	0.63 (0.5–3.0)	113 (99)	NR	NR
0.3	5	6.86 (21)	1.03 (1.0–3.0)	1597 (35)	NR	NR
1	5	26.8 (13)	1.27 (1.0–4.0)	7377 (17)	8537 (19)	20.6 (2.81)
3	5	80.4 (14)	1.50 (1.3–3.0)	26,189 (17)	28,799 (19)	23.2 (4.69)
10	20	195 (20)	1.75 (1.0–48.0)	74,378 (17)	81,022 (20)	23.7 (4.41)

a Two subjects in the 0.03 mg/kg dose cohort had serum drug concentrations below the assay lower limit of quantitation for all time points and were excluded from pharmacokinetic statistical analyses.

NR = Not reported; the AUC_INF_ extrapolated area was >20% of the total area resulting in undue potential for error.

## Discussion

These data describe the proof-of-concept evaluation of an anti-PD-1 monoclonal antibody in patients with chronic viral infection. Following administration of a single dose of BMS-936558, HCV RNA reductions ≥0.5 log_10_ IU/mL were observed on ≥2 consecutive visits (protocol-defined clinical response) in five of 45 (11.1%) patients. At the highest administered dose (10 mg/kg), HCV RNA reductions >4 log_10_ IU/mL were observed in three patients. Thus, in this high-dose group, 3/20 patients, or 15%, experienced significant reductions of HCV RNA. Suppression of HCV replication persisted more than eight weeks in most patients, consistent with the hypothesized immunologic mode of action as well as the long serum half-life of BMS-936558 [40]. HCV RNA was below the assay lower limit of quantitation (25 IU/mL) in two patients at the end of the 12-week study follow-up, one of whom, a prior null responder to interferon-alfa, progressed to undetectable HCV RNA and remained so over one year later.

HCV RNA generally varies <0.5 log_10_ IU/mL in patients with established HCV infection and spontaneous remissions are rare [38], [45], therefore it appears that the observed reductions in virus titer are related to treatment with BMS-936558. While conclusions are limited by the small number of patients treated in this study, responders did not have a higher rate of immune-related adverse events and neither BMS-936558 exposure nor immunogenicity predicted response. The modest rate of response may have been due, at least in part, to the administration of only a single dose to establish proof of concept. In addition, several other processes affecting the HCV-specific host immune response may conceivably have contributed to these observations, though data are lacking in this small patient set. For example, response may be influenced by levels of PD-1 ligand expression in the liver or lymphoid organs. The effectiveness of anti-PD-1 blockade could be limited by the emergence of HCV variants with T cell escape mutations conferring reduced immunogenicity and recognition by T cells targeting the original virus. Finally, other cell surface inhibitory receptors in addition to PD-1–such as lymphocyte activation gene-3 (LAG-3), T cell immunoglobulin mucin-3 (TIM-3), and cytotoxic T-lymphocyte-associated antigen-4 (CTLA-4) – are implicated in the co-regulation of the T cell exhaustion phenotype [46], [47], and their influence may also limit the effectiveness a single-locus blockade of PD-1. It is noteworthy that in *ex vivo* studies of T-cells from patients with chronic HCV infection, the modest functional impairment of circulating CD8^+^ T-cells could be reversed by PD-1 blockade alone, whereas the marked dysfunction of intrahepatic CD8^+^ T-cells, which expressed very high levels of PD-1, could not [33]. However, impairment of both circulating and intrahepatic CD8^+^ cells in HCV infection could be reversed by simultaneous blockade of both PD-1 and CTLA-4 [38].

Several immunologic findings were noteworthy. First, as might be expected from a human monoclonal antibody, human-anti-human (HAHA) antibody responses were not detected in most patients through 85 days post-dose, suggesting such responses did not influence safety or antiviral activity. Second, a transient decrease in CD4^+^, CD8^+^ and CD19^+^ lymphocytes was observed post treatment. These were changes were generally transient and not associated with adverse events suggesting immune deficits. Similar observations have been made in cancer patients following anti-PD-1 administration [21] and were hypothesized to represent redistribution of lymphocyte subsets into tumor and tissue compartments. Finally, the immunoadjuvant activity of BMS-936558 on anti-tetanus toxoid antibody levels was explored. Marked increases in anti-tetanus antibody levels over pre-treatment baseline were seen in several patients who received 3.0 mg/kg BMS-936558 and 10 mg/kg which exceeded changes observed in patients who received lower doses or placebo. A finding of an effect of anti-PD-1 blockade on antibody titers would be consistent with a role for PD-1 in B cell as well as T follicular helper cell function as has been described [48], [49]. Increases in anti-SIV titers have previously been shown in SIV-infected macaques treated with an anti-PD-1 antibody [36].

A single dose of BMS-936558 was generally well tolerated; most common adverse events were non-specific in nature. Immune-related adverse events were anticipated with this intervention; such events could result from a break in host tolerance to self-antigens or from a liver-directed antiviral T-cell response following reversal of immune suppression. Overall, immune-related events were infrequent, mostly mild to moderate in intensity, and generally similar to those reported in studies of BMS-936558 in patients with solid tumors [21]. Most immune-related events resolved spontaneously.

Endocrine abnormalities, including thyroid abnormalities, can be autoimmune in nature and potentially indicate altered immunologic self-tolerance. Two patients experienced low TSH levels (hyperthyroidism) followed by abnormally high TSH; one also experienced increased titers of ATGA and symptoms consistent with hyperthyroidism. This pattern resembles some cases of immune-mediated thyroiditis seen in clinical practice and would be consistent with reduced self-tolerance to thyroid specific antigens. Administration of anti-PD-1 therapy requires a vigilant follow up for signs and symptoms consistent with autoimmune disease.

One patient who received BMS-936558 10 mg/kg and achieved a significant clinical response had a marked grade 4 ALT elevation. In studies of solid tumors, ALT elevations have been observed in the absence of identifiable hepatic metastases, indicating the potential for non-specific hepatic inflammation. The ALT elevation in this subject occurred concomitant with a >4 log_10_ reduction of HCV RNA, a finding consistent with activation of HCV-specific, liver-directed T cell-mediated immunity in acute HCV infection [50]. Thus, it is possible that this ALT elevation was a specific result of the predicted mechanism of action of BMS-936558. Importantly, bilirubin and measures of hepatic function remained stable and the event resolved without intervention, concomitant with a return of HCV RNA towards baseline levels.

In summary, this study establishes proof-of-concept that PD-1 blockade with BMS-936558 can lead to persistent suppression of HCV replication in some patients with chronic infection, including those who do not respond to interferon-alfa therapy. While the therapeutic application of this approach in HCV infection is limited by the recent, ongoing development of highly effective new treatments, the promising results of this study suggest that further exploration of PD-1 pathway blockade is warranted across other chronic viral diseases, possibly in combination with other immunomodulatory or direct-acting antiviral agents. The potential for autoimmune adverse events, or of activity-related hepatic inflammation in liver infections, will require further evaluation in a larger dataset following multiple-dose administration.

## Supporting Information

CONSORT Checklist **Checklist S1**
(DOC)

Protocol S1 **Trial Protocol**
(PDF)
