# Supplementary material for: A Randomized, Double-Blind, Placebo-Controlled Assessment of BMS-936558, a Fully Human Monoclonal Antibody to Programmed Death-1 (PD-1), in Patients with Chronic Hepatitis C Virus Infection
Source: PLoS One. 2013 May 22;8(5):e63818. doi: 10.1371/journal.pone.0063818 (PMC3661719; doi:10.1371/journal.pone.0063818)
Supplement: Protocol S1 — Trial Protocol (PDF) [file pone.0063818.s002.pdf]

MEDAREX

Clinical Protocol MDX1106-02 Amendment 04

A Phase 1, Double-blind, Randomized, Multicenter, Placebo-controlled, Safety and Pharmacokinetic Dose-escalation Study of a Single Intravenous Administration of MDX-1106, a Fully Human Monoclonal Antibody to PD-1, in Subjects with Active Hepatitis C Genotype 1 Infection

Medical Monitor

Israel Lowy, MD, PhD
Senior Director, Clinical Science
Office Phone Number: +1-908-479-2447
Office Fax Number: +1-908-479-2410
Mobile Phone Number: +1-908-770-6477
Email: ilowy@medarex.com

Clinical Operations Contact

Araceli Doronila, BS, CNMT
Manager, Clinical Operations
Office Phone Number: +1-908-479-2630
Office Fax Number: +1-908-479-2415
Mobile Phone Number: +1-908-770-5637
Email: adoronila@medarex.com

SAE Reporting FAX Number
+1-908-479-2408

Central Emergency Phone Number
+1-800-246-2912

If any Medarex contact information is changed during the course of the study, this will be done by Medarex, with written notification to the Investigator(s), and will not require (a) protocol amendment(s).

Replace all previous version(s) of the protocol with this revised Protocol Amendment 04. Please provide a copy of this revised protocol to all study personnel under your supervision.

Property of Medarex, Inc.
519 Route 173 West
Bloomsbury, NJ 08804

Confidential

The information in this document contains trade secrets and commercial information that are privileged or confidential and may not be used, divulged, published, or otherwise disclosed without the written consent of Medarex, Inc. These restrictions on disclosure will apply equally to all future information supplied to you or generated by you in connection with the study.

TABLE OF CONTENTS

SYNOPSIS 5

ABBREVIATIONS 9

TIME AND EVENTS SCHEDULE 11

1. INTRODUCTION AND RATIONALE 17
1.1. Overview 17
1.2. PD-1 and the Immune Response 17
1.2.1. PD-1 and Immunotherapy of Chronic (Viral) Infections 20
1.2.2. PD-1 and Immunotherapy of Tumors 22
1.3. MDX-1106 23
1.3.1. Summary of Clinical Safety in Oncology 23
1.3.2. Rationale for MDX-1106 Dosage Selection in Hepatitis C 25

2. STUDY OBJECTIVES 25
2.1. Primary Objective(s) 25
2.2. Secondary Objective(s) 26

3. OVERVIEW OF STUDY DESIGN 26

4. STUDY POPULATION 28
4.1. Inclusion Criteria 29
4.2. Exclusion Criteria 30

5. RANDOMIZATION AND BLINDING 32

6. ASSIGNMENT TO STUDY 33

7. DOSAGE AND ADMINISTRATION 33
7.1. Dosing Overview 33
7.2. Packaging and Labeling 35
7.3. Storage 35
7.4. Inventory 35
7.5. Dispensing Supplies 35
7.6. Ordering Study Medication 36

8. MDX-1106: TOXICITY, AND MANAGEMENT 36
8.1. Dose Escalation 36
8.2. Dose-limiting Toxicity 36
8.3. Stopping Rules for Dose-limiting Toxicity and Delayed Dose-limiting Toxicity 37
8.4. Stopping Rules for the Expansion Cohorts 37
8.5. Infusion Reactions 38
8.6. Possible Toxicities 39
8.6.1. Hepatotoxicity Evaluation 41
8.6.2. Rationale for Consideration of Corticosteroid Treatment of Serious Immune Related Adverse Events (e.g., Hepatitis, or other) in Subjects with Active Hepatitis C Infection 43

9. COMPLIANCE 43

TABLE OF CONTENTS *(continued)*

10. CONCOMITANT THERAPY....44

11. STUDY EVALUATIONS....45
11.1. Study Procedures by Visit....45
11.1.1. Overview....45
11.1.2. Screening Period....45
11.1.3. Pre-treatment Period....47
11.1.4. Treatment Period....48
11.1.5. Follow-Up Period....50
11.1.6. Off Study Visit....51
11.2. Pharmacokinetic Evaluations....53

12. EFFICACY EVALUATIONS....53
12.1. Primary Efficacy Parameter....53
12.2. Secondary Efficacy Parameter....53

13. Safety Evaluations....54
13.1. Safety Precautions....54

14. ADVERSE EVENT REPORTING....54
14.1. Definitions....55
14.2. Protocol-Defined Adverse Events of Importance....56
14.3. Serious Adverse Events....57
14.4. Instructions for Rapid Notification of Serious Adverse Events....57
14.4.1. Reporting Responsibility....57
14.4.2. Reporting Procedures....58
14.4.3. Overdose....58
14.4.4. Pregnancy....58
14.4.4.1. Reporting of Pregnancy....59
14.4.5. Contact Persons and Numbers....59

15. STATISTICAL METHODS....59
15.1. Sample Size Determination....59
15.2. Study Populations....60
15.2.1. Safety Population....60
15.2.2. Per-protocol Population....60
15.2.3. Pharmacokinetic Population....60
15.3. Statistical Consideration....60
15.3.1. Demographics and Baseline Characteristics....61
15.3.2. Extent of Exposure....61
15.3.3. Concomitant Medication....61
15.3.4. Efficacy....61
15.3.5. Safety....62
15.3.6. Pharmacokinetic and Immunogenicity Parameters....63
15.4. Statistical Software....63

16. ETHICAL ASPECTS....64
16.1. Ethics and Good Clinical Practice....64

TABLE OF CONTENTS *(continued)*

16.2. Institutional Review Board/Independent Ethics Committee....64
16.3. Informed Consent....64

17. ADMINISTRATIVE REQUIREMENTS....65
17.1. Protocol Amendments....65
17.2. Monitoring Procedures....66
17.3. Recording of Data and Retention of Documents....66
17.4. Auditing Procedures....67
17.5. Publication of Results....67
17.6. Disclosure and Confidentiality....68
17.7. Discontinuation of Study....68
17.8. Data Management....68
17.8.1. Data Collection....68
17.8.2. Database Management and Quality Control....68

18. REFERENCES....70

19. APPENDICES....74
Appendix 1: Categories of Prior HCV Treatment Experience....75
Appendix 2: Instructions for Placing and Reading the DTH Skin Tests for Tetanus and *Candida* Antigens....76
Appendix 3: DMID Adult Toxicity Tables....80
Appendix 4: Pre-existing Autoimmune Diseases....90
Appendix 5: List of Immune-Related Adverse Event Symptoms....91

SYNOPSIS

TITLE

A Phase 1, Double-blind, Randomized, Multicenter, Placebo-controlled, Safety and Pharmacokinetic Dose-escalation Study of a Single Intravenous Administration of MDX-1106, a Fully Human Monoclonal Antibody to PD-1, in Subjects with Active Hepatitis C Genotype 1 Infection

PROTOCOL NUMBER

MDX1106-02

OBJECTIVES

Primary:

The primary objective of the study is to examine the safety, tolerability, and immunogenicity of a single dose of MDX-1106.

Secondary:

The secondary objectives of the study are to determine:

1) The pharmacokinetic profile of a single dose of MDX-1106;
2) Changes in Hepatitis C viral load from baseline, by magnitude and duration, and in relation to the administered dose of MDX-1106;
3) The proportion of subjects in each dosing cohort who experience a 0.5-log or greater decline from baseline viral load, that is repeated on at least 2 consecutive measures;
4) Changes in levels of serum cytokines and peripheral blood mononuclear cell (PBMC) reactivity to Hepatitis C viral antigens from baseline and in relation to MDX-1106 dose; and
5) Changes in delayed-type hypersensitivity (DTH) reactivity to a *Candida*/tetanus antigen skin test, anti-tetanus antibody titers, and PBMC cellular response to common recall antigens in relation to the MDX-1106 dose.

OVERVIEW OF STUDY DESIGN

This is a Phase 1, randomized, double-blind, placebo-controlled, multicenter, single-dose, dose-escalation, safety and pharmacokinetic study of MDX-1106. Subjects who failed or relapsed after a prior interferon based therapy will be enrolled into 1 of 6 escalating dose cohorts. The first cohort will receive 0.03 mg/kg of MDX-1106 or placebo as an intravenous (i.v.) infusion; subsequent cohorts will receive 0.1, 0.3, 1, 3, or 10 mg/kg of MDX-1106 or placebo. In the first 2 cohorts, no more than 1 subject will receive study drug on the same day. Each cohort will enroll 6 subjects (subjects will be randomized such that 5 subjects will receive MDX-1106 and 1 subject will receive placebo). Each subject will receive a single dose of study drug during the study.

Subjects who withdraw from the study prior to Day 29 for reasons other than safety concerns will be replaced. After the dose escalation cohorts are enrolled (up to 36 subjects) 6 additional interferon experienced subjects will be enrolled in an expansion cohort to obtain further safety information and a preliminary estimate of efficacy. These subjects will be randomly assigned to receive either placebo (1 subject) or MDX-1106 (5 subjects) at or below the maximum tolerated dose (MTD), or the highest tested dose if the MTD is not identified. An additional cohort of 12 subjects who are interferon naïve will be enrolled in parallel with the interferon experienced expansion cohort, and randomly assigned to receive either placebo (2 subjects) or MDX-1106 (10 subjects) at the same dose level as the expansion cohort.

The MTD is the highest dose where no subject has experienced a DLT. The MTD may or may not be definitively identified by the initial dose levels.

The study will consist of Screening, Pre-treatment, Treatment, and Follow-up Periods. Beginning with the 0.03 mg/kg dose, MDX-1106 will be administered as an i.v. infusion. Subjects will be monitored for infusion reactions over a 6-hour observation period at the investigative unit (i.e., hospital or clinic unit with resuscitative capabilities) with ready access to an intensive care unit. Subjects who experience an adverse event, including an infusion reaction of Grade 2 during the 6 hour post dose observation period that does not resolve during this time, or any adverse event of Grade ≥ 3 regardless of resolution, will be observed for a minimum of an additional 18 hours or until the adverse event resolves with vital sign measurements every 4 hours and additional evaluations as medically indicated for the management of the adverse event. If there are 2 or more ≥ Grade 2 infusion reactions in a cohort, all subsequent subjects will be pre-medicated with diphenhydramine and acetaminophen. Prior to discharge, subjects will be informed regarding a wide range of symptoms that may be associated with the development of late-occurring hypersensitivities and immune related adverse events (irAEs) (defined as a clinically significant adverse event of any organ that is associated with drug exposure, of otherwise unknown etiology, and consistent with an immune-mediated mechanism), and will be given instruction regarding when they should contact the Investigator and/or report directly to the emergency room.

No 2 subjects will be treated on the same day in the first 2 cohorts. At any dose level, dosing of subjects at the next higher dose level will not be initiated until all 6 subjects in a cohort have been dosed and observed for 4 weeks in the absence of a dose-limiting toxicity (DLT). A DLT is defined as an adverse event or clinically significant laboratory abnormality that emerges within 28 days of dosing with MDX-1106, and whose severity is ≥ Grade 3 and could be considered even remotely related to study treatment. If an event is deemed a DLT, Medarex will unblind the subject to confirm that MDX-1106 was administered. All ≥ Grade 2 infusion reactions that occur during the study will be evaluated as to whether or not the event is a DLT, and reviewed with the Division of Antiviral Products (DAVP) of the Food and Drug Administration (FDA). Events clearly considered unrelated to treatment (e.g., automobile accident, or occurring after administration of placebo) will not be considered a DLT. If 1 DLT is observed in any subject, that cohort will have exceeded the MTD and no further dose escalation will occur. If the previous dose level was well tolerated and no subjects experienced a DLT at that level, an intermediate dose level may be defined by protocol amendment in consultation with the FDA. The MTD will be the highest dose where no subjects have experienced a DLT.

If a DLT occurs in the expansion cohort or the interferon naïve cohort, enrollment will be interrupted. The event will be reviewed (which may include unblinding), by Medarex, the Principal Investigator, and DAVP, (with subsequent IRB notification), to determine if enrollment may continue at that dose, or stopped and a new expansion cohort initiated at a lower dose. A delayed DLT is defined as an event that otherwise meets DLT criteria, but occurs 28 days after MDX-1106 administration. Delayed DLTs will not automatically impact upon dose escalation, but will be collected and evaluated by the Investigators and Medarex on an ongoing basis. If 2 or more such delayed DLTs are noted within a cohort, accrual will be held pending analysis (which may include unblinding), and will be restarted only with Investigator, Medarex, and DAVP approval (with subsequent IRB notification). The potential of a delayed DLT arising after completion of the planned 12-week study period is exceedingly unlikely, as 12 weeks is well in excess of 5 times the anticipated 2-week half-life of MDX-1106.

During the Pre-treatment Period (within 2 to 5 days prior to infusion), placement of DTH skin tests will occur; these skin tests will be read 48 to 72 hours later, just prior to dosing with MDX-1106, and measured again at 24 and 48 hours after dosing. A repeat DTH skin test will be placed on Day 29 and measured 48 to 72 hours later, on Day 31. Blood (for biochemistry and hematology, including liver function tests [LFTs] and HCV viral loads) will be collected at Screening, pretreatment, and on Days 1, 2, 3, 8, 15, 22, 29, 43, 57, and 85, and urine samples will be collected at Screening, pretreatment, and on Days 1, 3, 8, 29, and 85. Tests for drug and alcohol abuse will be conducted at Screening and prior to

study dosing, and at the time of an adverse event if the Investigator considers a causal relationship of the event to drug or alcohol abuse. Positive screens for drugs that the subject is not known to be taking are exclusionary or protocol violations. If there is uncertainty regarding interpretation of the urine drug screen, it should be repeated to confirm the result and, in the case of a conflicting result, a third test should be done and the consensus result based on 2 of 3 findings. Immunogenicity testing will be performed on Days 1, 29, and 85; immune safety tests will be performed during the Pre-treatment Period and on Days 8, 29, and 85; and PBMCs for cryopreservation, lymphocytes for phenotype analysis by fluorescent-activated cell sorter (FACS), and serum for cytokines, and for tetanus antibody titer will be collected during the Pre-treatment Period and on Days 1, 2, 3, 8, 15, 29, 57, and 85. Prothrombin time and INR will be measured at Screening and on Days 8, 29, and 85. Subjects will be evaluated for vital signs, adverse events, and concomitant medication use at each visit. Evaluation of safety and tolerability will include assessment of adverse events, toxicity (regardless of Investigator/Medarex attribution to MDX-1106), physical examination including vital sign measurements, electrocardiogram (ECG) measurement, and blood and urine sampling for clinical laboratory parameters.

STUDY POPULATION

Up to 42 subjects with active Hepatitis C genotype 1 or mixed Hepatitis C genotype (e.g., 1/2, 1/3, 1/4, etc., only if genotype 1 can be shown to be present) infection who received previous therapy with interferon and ribavirin or peginterferon and ribavirin without a sustained virological response (SVR), or who relapsed following an SVR will be enrolled in the study. An additional 12 subjects who are naïve to an interferon based therapy will also be enrolled in the study. The duration of participation of an individual subject in the study from screening to completion will be up to 16 weeks, and the overall study is expected to take up to approximately 18 months to reach last subject, last visit.

DOSAGE AND ADMINISTRATION

MDX-1106 or placebo will be administered as an i.v. infusion, with MDX-1106 dosing of the first cohort to begin at a level of 0.03 mg/kg. Subsequent cohorts of subjects will be administered MDX-1106 at escalating dose levels of 0.1, 0.3, 1, 3, and 10 mg/kg.

PHARMACOKINETIC EVALUATIONS

Serum concentrations of MDX-1106 will be assessed on Day 1 at pre-infusion and 1, 1.25, 1.5, 2, 3, 4, 6, 24, and 48 hours post infusion start time. [If the infusion is slowed beyond the 60-minute timeframe, then the post-infusion samples will be collected relative to the infusion end time (at end of infusion, 0.25, 0.5, 1, 2, 3, 5, 23, and 47 hours after end of infusion)]. Additional samples will be taken at 0.25, 0.5, and 0.75 hours post infusion start time for subjects in the first cohort (0.03 mg/kg) and at 0.5 and 0.75 hours post infusion start time for subjects in the second cohort (0.1 mg/kg). Single serum samples for pharmacokinetic parameters will be taken on Days 8, 15, 22, 29, 43, 57, and 85.

SAFETY EVALUATIONS

Assessment of safety will be determined by clinical laboratory tests (blood and urine sampling for clinical laboratory parameters), immunogenicity evaluations, physical examinations including vital sign measurements, ECG, and the incidence and severity of adverse events.

EFFICACY EVALUATIONS

The primary efficacy parameter is the proportion of MDX-1106-treated subjects in each dosing cohort and placebo-treated subjects who experience a 0.5-log or greater decline from baseline viral load, that is repeated on at least 2 consecutive measures.

The secondary efficacy parameters include the following: 1) changes in Hepatitis C viral load from baseline, by magnitude and duration, and in relation to the administered dose of MDX-1106 or placebo; 2) changes in levels of serum cytokines and PBMC reactivity to Hepatitis C viral antigens from baseline and in relation to MDX-1106 or placebo dose; and 3) changes in DTH reactivity to a *Candida*/tetanus antigen skin test, anti-tetanus antibody titers, and PBMC cellular response to common recall antigens in relation to the MDX 1106 or placebo dose.

STATISTICAL METHODS

The sample size of up to 54 subjects (up to 36 subjects in the dose-escalation phase, 6 subjects in the expansion cohort, and 12 subjects in the interferon naïve cohort) for this study is based on the study design for dose escalation, safety evaluation requirements, and preliminary estimate of efficacy, and is not determined from power analysis. The safety parameters include adverse events, vital sign measurements, clinical laboratory tests, immunogenicity assays, electrocardiogram measurements, and physical examinations. Efficacy parameters include the activity of MDX-1106 to alter the Hepatitis C viral load, levels of serum cytokines, antibody titer to tetanus, PBMC reactivity to Hepatitis C viral antigens and common recall antigens; and changes in DTH reactivity to *Candida*/tetanus antigen test in relation to the MDX-1106 dose. Pharmacokinetic parameters include t_{max}, C_{max}, $t_{1/2}$, $AUC_{0\text{-}t}$, and AUC_{inf}. All parameters will be summarized by dose cohort using descriptive statistics including 95% confidence limits around mean changes if applicable.

ABBREVIATIONS

Abbreviation	Term
APC	Antigen-presenting cells
ASM	Anti-smooth muscle antibody titer
AUC	Area under the curve
BUN	Blood urea nitrogen
CBC	Complete blood count
CHO	Chinese hamster ovary
C_{max}	Maximum concentration
CMV	Cytomegalovirus
CRF	Case report form
CRP	C-reactive protein
DC	Dendritic cell
DAVP	Division of Antiviral Products
DCM	Dilated cardiomyopathy
DLT	Dose-limiting toxicity
DMID	Division of Microbiology and Infectious Disease
DTH	Delayed-type hypersensitivity
ECG	Electrocardiogram
EDC	Electronic data capture
ER	Emergency room
FACS	Fluorescent-activated cell sorter
FDA	Food and Drug Administration
GGT	Gamma-glutamyl transpeptidase
GVHD	Graft-versus-host disease
HBsAg	Hepatitis B surface antigen
HCV	Hepatitis C virus
HBV	Hepatitis B virus
HEENT	Head, eyes, ears, nose and throat
HIPAA	Health Information Portability and Accountability Act
HIV	Human immunodeficiency virus
HuMAb	Fully human monoclonal antibody
i.v.	Intravenous
IFN	Interferon
INR	International normalized ratio

Abbreviation	Term
irAE	Immune-related adverse event
IRB/IEC	Institutional review board/independent ethics committee
ITIM	Immunoreceptor tyrosine inhibitory motif
ITSM	Immunoreceptor tyrosine-based switch motif
LCMV	Lymphocytic choriomeningitis virus
LDH	Lactate dehydrogenase
LFT	Liver function test
MHC	Major histochemical complex
MTD	Maximum-tolerated dose
PBMC	Peripheral blood mononuclear cell
PD-1	Programmed death-1
PPD	Purified protein derivative
PVG	Pharmacovigilance
SGOT (AST)	Serum glutamic-oxaloacetic transaminase (Aspartate aminotransferase)
SGPT (ALT)	Serum glutamic-pyruvic transaminase (Alanine aminotransferase)
SOP	Standard operating procedures
SVR	Sustained virological response
TCR	T-cell receptor
t_{max}	Time at which C_{max} occurs
TNF	Tumor necrosis factor
TSH	Thyroid stimulating hormone

TIME AND EVENTS SCHEDULE

Table 1: Time and Events Schedule

**Examination**	**Screening**	**Pre-treatment**	**Treatment**			**Follow-up**							**Off Study**
**Timepoint (Day)**	**Day –28 to Day –6**	**Day –5 to Day –2**	**1[1]**	**2**	**3**	**8[2]**	**15[2]**	**22[2]**	**29[2]**	**31**	**43[3]**	**57[3]**	**85**
**Visit**	**1**	**2**	**3**	**4**	**5**	**6**	**7**	**8**	**9**		**10**	**11**	**12**
MDX-1106 or placebo infusion			**X**										
Informed consent/ HIPAA authorization[4]	**X**												
Inclusion/exclusion criteria	**X**												
Demographics and medical history[5]	**X**												
Serum β-HCG pregnancy test		**X**[6]											**X**[6]
Vital sign measurements[7]	**X**	**X**	**X**[8]	**X**	**X**	**X**	**X**	**X**	**X**		**X**	**X**	**X**
Physical examination	**X**[9]	**X**[10]	**X**[10]	**X**[10]	**X**[10]	**X**[10]	**X**[10]	**X**[10]	**X**[10]		**X**[10]	**X**[10]	**X**[9]
Chest radiograph	**X**												**X**
Electrocardiogram	**X**			**X**									**X**
Liver biopsy[11]	**X**												
Hematology	**X**	**X**	**X**[12]	**X**	**X**	**X**	**X**	**X**	**X**		**X**	**X**	**X**
Biochemistry	**X**	**X**	**X**[12]	**X**	**X**	**X**	**X**	**X**	**X**		**X**	**X**	**X**
Urinalysis	**X**	**X**	**X**[12]		**X**	**X**			**X**				**X**
Drug and alcohol tests	**X**		**X**[13]										
α-fetoprotein	**X**												

continued

Table 1: Time and Events Schedule

**Examination**	**Screening**	**Pre-treatment**	**Treatment**			**Follow-up**							**Off Study**
**Timepoint (Day)**	**Day –28 to Day –6**	**Day –5 to Day –2**	**1[1]**	**2**	**3**	**8[2]**	**15[2]**	**22[2]**	**29[2]**	**31**	**43[3]**	**57[3]**	**85**
**Visit**	**1**	**2**	**3**	**4**	**5**	**6**	**7**	**8**	**9**		**10**	**11**	**12**
Viral markers – HIV and Hepatitis B	X												
Prothrombin time and INR	X					X			X				X
Immune safety assays[14]		X				X			X				X
Serum sample – pharmacokinetics			X[15]	X	X	X	X	X	X		X	X	X
Immunogenicity assays			X[16]						X				X
HCV genotype	X												
HCV viral load	X	X	X[15]	X	X	X	X	X	X		X	X	X
DTH skin test		X[17]	X[18]	X[18]	X[18]				X[17]	X[18]			
Lymphocyte FACS		X	X[12]	X	X	X	X		X			X	X
Cryopreserved PBMCs		X	X[12]	X	X	X	X		X			X	X
Serum for cytokines, anti-tetanus antibody		X	X[12]	X	X	X	X		X			X	X
Concomitant medications	X	X	X	X	X	X	X	X	X		X	X	X
Adverse events[19]	X	X	X	X	X	X	X	X	X		X	X	X

Key: DTH = delayed-type hypersensitivity; FACS = fluorescent-activated cell sorter; HCV = Hepatitis C Virus; HIPPAA = Health Insurance Portability and Accountability Act; INR = International Normalized ratio; PBMC = peripheral blood mononuclear cell

Footnotes can be found on the following page.

TABLE 1 FOOTNOTES

1 Subjects will be monitored for 6 hours post dose (with a minimum additional 18 hours for subjects with unresolved infusion reactions of Grade 2 or infusion reactions that are Grade ≥ 3 regardless of resolution.). Prior to discharge, subjects will be informed regarding a wide range of symptoms that may be associated with the development of late-occurring hypersensitivities and irAEs, and will be given instruction regarding when they should contact the Investigator and/or report directly to the emergency room.

2 Visit to occur within +/- 1 day.

3 Visit to occur within +/- 2 days.

4 Informed consent/HIPAA must be signed before the initiation of all study treatment and procedures. May be signed prior to Day -28.

5 To include all prior treatment for Hepatitis C.

6 Serum β-HCG pregnancy test within 2 to 5 days prior to infusion and at Visit 12 for all women except those who have had a hysterectomy or bilateral oophorectomy, who are post menopausal (> 24 months since last menses), or who are > 60 years of age.

7 Subjects with an extended observation period (i.e., those subjects with unresolved infusion reactions of Grade 2 or infusion reactions that are Grade ≥ 3 regardless of resolution) will have additional vitals taken at least every 4 hours for the remainder of the observation period.

8 On the day of infusion, vital sign measurements will be collected prior to the infusion and every 15 minutes during the infusion and for a half hour post-infusion end-time, and at 2, 3, 4, and 6 hours post infusion start time (see Table 2).

9 Complete physical examination (including examination of skin, head, eyes, ears, nose, throat [HEENT]), neck, joints, lungs, heart, abdomen (including liver and spleen), stool for occult blood, lymph nodes, and extremities. Neurological examination should include assessment of cranial nerves, motor, sensory, and deep tendon reflexes.

10 Abbreviated physical examination: at minimum to include eyes, throat, neck/lymph nodes, chest, abdomen and skin, and others as directed by symptoms. Abnormal findings from screening exam considered of potential significance to course of Hepatitis C disease should be explicitly followed.

11 To be performed if not done within the past 2 years. A separate informed consent, provided by the site, must be signed by the Subject before the liver biopsy is performed.

(continued)

TABLE 1 FOOTNOTES *(continued)*

[12] To be drawn/collected 4 hours post infusion start time.

[13] Urine drug screens, provided by the central laboratory, should be done at Screening, just prior to infusion, and at any later time as required (e.g., at the time of an adverse event if the Investigator considers a causal relationship of the event to drug or alcohol abuse). Positive screens for drugs that the subject is not known to be taking are exclusionary or protocol violations. If there is uncertainty regarding interpretation of the urine drug screen, it should be repeated to confirm the result, and in the case of a conflicting result, a third should be done and the consensus result based on 2 out of 3 findings. Alcohol blood test at Screening, just prior to dosing, and at any later time as required.

[14] Autoimmune sera to be drawn and stored for subsequent batch analysis for additional tests as necessary.

[15] Initial sample to be drawn prior to infusion, and subsequent PK or viral load samples to be drawn as outlined in Table 2.

[16] Predose only.

[17] Quantitative DTH tests will involve placement of skin tests for Tetanus and *C. albicans* antigens at Visits 2 and 9, as outlined in Appendix 2.

[18] Measurement of induration at 48 to 72 hours post placement at Visit 2 (AND must be read prior to infusion if not included in the 48 to 72 hour timepoint), 24 and 48 hours post infusion start time (Visits 4 and 5), and 48 to 72 hours post placement at Visit 9. Measurements to be performed and recorded as outlined in Appendix 2.

[19] After signing the informed consent and HIPAA authorization, but prior to treatment with study medication, adverse events will be recorded on the appropriate Medical History CRF. Adverse event assessment includes specific elicitation of symptoms that may be indicative of immune related adverse events (see Appendix 5).

Table 2: Day 1 Detailed Time and Events Schedule

**Examination**	**Time (hours)**										
	**0**	**0.25**[1]	**0.5**[1]	**0.75**[1]	**1**[1]	**1.25**[1]	**1.5**[1]	**2**[1]	**3**[1]	**4**[1]	**6**[1]
MDX-1106 infusion –0.03 mg/kg or placebo	**X------------X**										
MDX-1106 infusion – 0.1 mg/kg or placebo	**X---------------------X**										
MDX-1106 infusion – ≥0.3 mg/kg or placebo	**X---X**										
Vital signs – all cohorts[2]	**X**[3]	**X**	**X**	**X**	**X**	**X**	**X**	**X**	**X**	**X**	**X**
Physical exam										**X**	
Serum sample – pharmacokinetics	**X**[3]	**X**[4]	**X**[5]	**X**[5]	**X**	**X**	**X**	**X**	**X**	**X**	**X**
Immunogenicity assay	**X**[3]										
Hematology										**X**	
Biochemistry										**X**	
Urinalysis										**X**	
Drug and alcohol screen	**X**										
Lymphocyte FACS										**X**	
Cryopreserved PBMCs										**X**	
Serum for cytokines and anti-tetanus antibody										**X**	
HCV viral load	**X**[3]									**X**	
Adverse events	**X**[3]	**X--X**									

Footnotes can be found on the following page.

TABLE 2 FOOTNOTES:

1 Denotes time post infusion start. If the infusion is slowed beyond the 60-minute timeframe, then the post-infusion samples will be collected relative to the infusion end time (at end of infusion, 0.25, 0.5, 1, 2, 3, and 5 hours after end of infusion).

2 Subjects with an extended observation period (i.e., those with unresolved infusion reactions of Grade 2 or infusion reactions that are Grade ≥ 3 regardless of resolution) will have additional vitals taken at least every 4 hours for the remainder of the observation period.

3 Assessment to be performed immediately pre-infusion.

4 Pharmacokinetic sample to be drawn immediately post infusion for the 0.03 mg/kg cohort only.

5 Pharmacokinetic samples to be drawn for both 0.03 and 0.1 mg/kg cohorts only.

1. INTRODUCTION AND RATIONALE

1.1. Overview

Persistence of chronic viral infections, such as Hepatitis B (HBV), Hepatitis C (HCV), and human immunodeficiency virus-1 (HIV-1) requires evasion of the adaptive immune response, in particular the cellular response of CD8+ and CD4+ T cells. A strong and durable viral antigen specific T-cell response is required for successful clearance and control of these infections. Although this response may also cause some bystander tissue damage, evidence, particularly in viral hepatitis, suggests that the major cause of tissue injury is the lack of a potent, viral antigen-specific T-cell response, allowing for viral persistence and recruitment of non-specific T cells, and the latter contributes to subsequent bystander pathology.[1] Therefore, enhancement of antigen-specific T-cell responses is a reasonable objective for immunotherapy approaches to these diseases.

Recent data have implicated a T-cell surface protein, programmed death-1 (PD-1), as a key mediator of inhibitory signals that limit the activity of viral antigen-specific T cells in chronic HCV, HBV and HIV, as well as in the murine lymphocytic choriomeningitis (LCMV) experimental model.[2] Elevated expression of PD-1 is found on viral antigen-specific T cells compared with that seen in the overall T cell population. Antibody-mediated blockade of the interaction of PD-1 with its main ligand, PD-L1, leads to an in vitro reversal of antigen-specific anergy. In vivo experiments have also shown an improvement in control of viremia when animals infected with LCMV are treated with antibodies that block this interaction.[3] Blockade of PD-1 may therefore be an effective approach to immunotherapy of chronic viral infections in subjects.

1.2. PD-1 and the Immune Response

Primary antigen-specific T-cell immune responses initiate after the integration of 2 signals received by the T cell from the antigen-presenting cell (APC).[4] Signal 1 is antigen specific, and is a result of the T-cell receptor (TCR) interacting with the (peptide) antigen displayed on APC in the context of the Major Histocompatibility Complex (MHC) Type I or Type II surface molecules, for CD8 and CD4 T cells, respectively. Signal 2 is not antigen specific, but is a costimulatory signal that arises from the interaction of the T cell CD28 surface molecule with the B7 molecule on the APC (either B7.1/CD80, or B7.2/CD86), and results in additional intracellular signals and secreted cytokines that drive an effective immune response. The absence of a costimulatory signal results in recognition without activation, or anergy, and may lead to death (by apoptosis) of antigen-specific T cells. Clearance of antigen is followed by the down-regulation of the activated T-cell response, mostly by apoptosis. A subpopulation of the

T cells matures into long-lived memory CD8 and CD4 cells that can then be promptly reactivated upon re-exposure to the antigen by the APC. It is believed that these regulatory mechanisms arose to maintain tolerance of the immune system to normal self antigens, while permitting it to effectively deal with abnormal or foreign antigens.

CD28, CD80, and CD86 are members of the immunoglobulin superfamily of costimulatory receptors. This family is quite large, and T-cell stimulation is a complex process involving the integration of numerous positive and negative costimulatory signals in addition to antigen recognition by the TCR (Figure 1).[5] Collectively, these signals govern the balance between T-cell activation and tolerance to antigens.

Figure 1: T-cell Stimulation

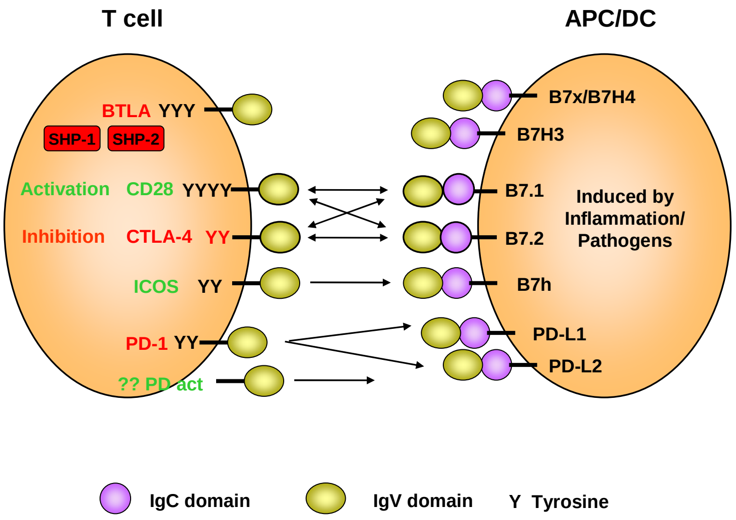

PD-1 (or CD279) is a member of the CD28 family of T-cell costimulatory receptors that include CD28, CTLA-4, ICOS, and BTLA. PD-1 is a 55 kD type I transmembrane protein that is part of the immunoglobulin gene superfamily.[6] PD-1 contains an intracellular membrane proximal immunoreceptor tyrosine inhibitory motif (ITIM) and a membrane distal tyrosine-based switch motif (ITSM). Two ligands specific for PD-1 have been identified: PD-L1 (also known as B7-H1 or CD274) and PD-L2 (also known as B7-DC or CD273). PD-L1 and PD-L2 have been shown to down-regulate T-cell activation upon binding to PD-1 in both murine and human systems.[7,8,9] PD-1 delivers a negative signal by the recruitment of the SHP-2 phosphatase to the phosphorylated tyrosine residue in the ITSM in its cytoplasmic region.[10,11] PD-1 is highly expressed on activated T cells and B cells.[6] PD-1 expression can also be detected on memory T cell subsets with variable levels of expression.

Further evidence for a negative regulatory role of PD-1 comes from studies of PD-1 null mice. PD-1-deficient mice develop various autoimmune phenotypes, including dilated cardiomyopathy (DCM), a lupus-like syndrome with arthritis and nephritis, and accelerated diabetes mellitus.[12,13,14] The emergence of these autoimmune phenotypes is dependent upon the genetic background of the mouse strain and many of these phenotypes emerge at different times and show variable penetrance. PD-1 deficiency on the C57BL/6 background results in development of a late-onset progressive arthritis and lupus-like glomerulonephritis,[12,13] while on the BALB/c background, it results in the development of a lethal DCM that shows incomplete penetrance, with concomitant evidence of autoantibodies to troponin-I.[13,15] As seen in Table 3, some inconsistencies in the phenotypes of the knockout strains have been noted, such as the difference in the phenotypes of the PD-1 knockout in Balb/c and 129 strains as well as the lack of phenotype in the PD-L1/PD-L2 knockout in Balb/c.

Table 3: Autoimmune Phenotypes in PD-1 and PD-L1/PD-L2 Null Strains[a]

Genotype	Phenotype	Age at onset	Penetrance/Comments	Ref
C57Bl/6-*Pcdcd1*$^{-/-}$	SLE-like	>6 months	~50%	12
BALB/c-*Pcdcd1*$^{-/-}$	Dilated cardiomyopathy	5-25 weeks	10%-60% - variable among different mouse colonies	13,16,17
	Gastritis	10-20 weeks	~80%	18
NOD-*Pdcd1*$^{-/-}$	Diabetes	4-10 weeks	100%	14
BALB/c-*Fcgr2*$^{-/-}$ *Pdcd1*$^{-/-}$	Hydronephrosis	10-20 weeks	35%	18
2C-*Pdcd1*$^{-/-}$ *H-2* $^{b/d}$	GVH-like	5-10 weeks	25%-100% - variable depending on genetic background	12,16
NOD-*PD-L1*$^{-/-}$*PD-L2*$^{-/-}$	Diabetes	6-8 weeks	100% - similar phenotype to PD-1 null mutation seen in Wang[9]	19
BALB/c-*PD-L1*$^{-/-}$ *PD-L2*$^{-/-}$	No phenotype	>8 months	100% - discrepancy regarding DCM seen with BALB/c-*Pcdcd1*$^{-/-}$	19
129svEv-Brd -*PD-1*$^{-/-}$	No phenotype	> 1 year	100% - no autoimmune phenotype (Note: this strain is a putative human PD-1 replacement which was shown to express no human or mouse PD-1)	20

[a] Adapted from Okazaki and Honjo[16]

In other murine models, PD-1 blockade has been found to play a role in the development of autoimmune diseases such as encephalomyelitis,[21] graft-versus-host disease (GVHD),[18] and type I diabetes.[22] The potential role of the PD-1/PD-L pathways in regulating tolerance and autoimmunity in humans has been recently summarized.[2]

1.2.1. PD-1 and Immunotherapy of Chronic (Viral) Infections

The role of PD-1 and PD-L1 in viral immunity has recently been investigated, and PD-1 expression has been found to be a critical mediator of T-cell unresponsiveness in the murine LCMV model system.[3] In this study, administration of an anti-PD-L1 antibody (as well as an anti-PD-1 antibody) which inhibits PD-1/PD-L1 interactions resulted in a diminution of LCMV viremia in an animal that had an established chronic infection. This study has been extensively reviewed and has generated a great deal of interest in the importance of this pathway in modulating the persistence of chronic viral infections.[23,24] PD-1 deficiency enhances anti-viral immunity at effector sites, resulting in rapid clearance of adenovirus in the liver.[25] In this report, it was noted that PD-L1 was expressed on vascular endothelium in peripheral tissues. Liver nonparenchymal cells, including sinusoidal endothelial cells and Kupffer cells, also constitutively expressed PD-L1 and inhibited proliferation and cell division of activated T cells expressing PD-1. The absence of PD-1 (in T cells derived from a knock-out mouse), as compared to wild-type T cells, enhanced the proliferation of effector T cells in the adenovirus infected liver and resulted in accelerated clearance of the virus. These results indicated that PD-1 plays an important role in T-cell tolerance at the effector phase and that blockade of the PD-1 pathway can augment antiviral immunity.

More recent data have implicated the role of PD-1 in human infections, particularly in HCV and HIV, and shown that HCV-specific CD8 cell exhaustion may represent a mechanism of HCV persistence.[26] PD-1 expression was studied longitudinally during acute HCV infection. Most HCV-specific CD8 cells expressed PD-1 at the time of acute illness, irrespective of the final outcome. PD-1 expression declined with the acquisition of a memory phenotype and recovery of an efficient CD8 cell function in resolving HCV infections, whereas high levels were maintained when HCV persisted and HCV-specific CD8+ T cells remained dysfunctional. Moreover, Investigators reported that the expression of CD127, a component of the IL-7 receptor, and a marker for a mature effector memory cell, correlated inversely with PD-1 expression, suggesting that PD-1 signaling marked a blockade in normal maturation that accompanied effective T cell control of HCV viremia. Blocking PD-1/PD-L1 interaction with an anti-PD-L1 antibody improved the capacity of expansion of virus-specific CD8 cells in vitro. Radziewicz et al.[27] compared peripheral and intrahepatic HCV-specific CD8+ T cells and found that in chronic HCV infection, peripheral HCV-specific T cells expressed high levels of PD-1 and that blockade

of the PD-1/PD-L1 interaction led to an enhanced proliferative capacity. Importantly, intrahepatic HCV-specific T cells, in contrast to those in the periphery, not only express high levels of PD-1 but also decreased IL-7 receptor alpha (CD127), an exhausted phenotype that is HCV antigen-specific and compartmentalized to the liver, the site of viral replication. Further, Investigators showed that the exhausted T-cell phenotype was exerting no selective pressure on HCV sequences, as the epitope to which the T cells were specific was abundant in the circulating HCV quasi-species. Corroborative data has recently been published by another group.[28]

Similar data implicating the PD-1 pathway has been found in the setting of chronic HIV-1 infection. In chronic HIV infection, HIV-specific CD8+ T cells are functionally impaired, showing a reduced capacity to produce cytokines and effector molecules as well as an impaired capacity to proliferate in response to HIV antigens and eliminate HIV infected cells. Trautman et al. reported that PD-1 expression levels were significantly correlated with both viral load and the reduced capacity for cytokine production and proliferation of HIV-specific CD8+ T cells.[29] Notably, cytomegalovirus (CMV)-specific CD8+ T cells from the same donors did not upregulate PD-1 and maintained the production of high levels of cytokines. Blocking PD-1 engagement to its ligand (PD-L1) enhanced the capacity of HIV-specific CD8+ T cells to survive and proliferate, leading to an increased production of cytokines and cytotoxic molecules in response to cognate antigen. Researchers concluded that accumulation of HIV-specific dysfunctional CD8+ T cells in the infected host could prevent the renewal of a functionally competent HIV-specific CD8+ repertoire. Day et al. reported that PD-1 is significantly upregulated on HIV-specific T cells, and expression correlates with impaired HIV-specific CD8 T-cell function. PD-1 expression on CD4 T cells also showed a positive correlation with viral load and an inverse correlation with CD4 T-cell count, and blockade of the pathway augmented HIV-specific CD4 and CD8 T-cell function.[30] Petrovas et al. found that PD-1 expression was found to be low on naive CD8+ T cells and increased on memory CD8+ T cells according to antigen specificity.[31] Memory CD8+ T cells specific for HIV antigens from poorly controlled chronically infected HIV subjects expressed greater levels of PD-1 than memory CD8+ T cells specific for a well-controlled persistent virus, such as CMV, or antigen-specific T cells for an acutely infectious virus, such as vaccinia. PD-1 expression was independent of maturational markers on memory CD8+ T cells and was not directly associated with an inability to produce cytokines. In their analysis, the level of PD-1 surface expression was the primary determinant of apoptosis sensitivity of virus-specific CD8+ T cells. Blockade of PD-1 led to changes in the ability of the cells to survive and expand, which, over several days, affected the number of cells expressing cytokines. They propose that PD-1 is a major regulator of apoptosis that can impact the frequency of antiviral T cells in chronic infections such as HIV, and could be manipulated to improve HIV-specific CD8+ T cell numbers. A recent report from this group also demonstrates

that the level of PD-1 on HIV specific CTL remained high in the presence of preserved viral epitope in the circulating quasispecies, whereas the level of PD-1 expression dropped after viral escape.[32] These results are similar to the HCV results reported by Radiewicz, et al.[27] and indicate that PD-1 expressing T cells are not exerting any selective pressure to drive viral escape on circulating virus. These reports of the involvement of the PD-1 pathway in chronic HIV infection have stimulated much interest and the potential importance of this pathway has been extensively reviewed by others.[33,34]

Sharpe et al.[2] have recently reviewed the data supporting the role of the PD-1 receptor and its ligands in regulating autoimmunity and infection. This review provides further references to the involvement of this pathway in LCMV, HCV and HIV, in other viral infections, such as HBV, respiratory syncytial virus, rhinovirus and herpes viruses, as well as in multiple non-viral infections, such as *Mycobacterium tuberculosis, Helicobacter pylori, Schistosoma mansoni* and *Leishsmania Mexicana*. Additional background material on the role of the PD-1/PD-L1 pathway may be found in the Investigators Brochure.

1.2.2. PD-1 and Immunotherapy of Tumors

Medarex opened an IND in multiple tumor types in July 2006 (100,052); a brief review of the data submitted to support the IND follows. The rationale for IND 100,052 was the potential role for PD-1 blockade as a tumor immunotherapy. Immunotherapy of tumors rests on the premise that tumors can be recognized as foreign rather than as self, and effectively attacked. Many tumors express tumor-specific antigens, and ongoing immune surveillance is believed to abort the progression of many tumors as they arise. Tumor progression may depend upon acquisition of mechanisms to evade an effective immune response.[35] Immune evasion may occur by exploiting any of the regulatory checkpoints that control the immune response, including the display of antigens and control of costimulatory pathways. Current immunotherapy efforts focus on the effective introduction of cancer antigens via therapeutic vaccination, and the modulation of regulatory checkpoints by costimulation and cytokine manipulation in order to break the apparent tolerance of the immune system to tumor antigens.

Several published murine tumor studies using anti-PD-1 and anti-PD-L1 antibodies or PD-1 null mice support the role of this pathway for therapeutic intervention in cancer. Two metastatic models have been shown to be sensitive to PD-1 blockade.[36] Studies reveal that antitumor activity by PD-1 blockade functions in PD-L1+ tumors as well as in tumors that are negative for the expression of PD-L1.[37,38,39,40,41]

In humans, constitutive PD-L1 expression is normally limited to macrophage-lineage cells, although expression of PD-L1 can be induced on other cells of hematopoietic origin as well, including activated T cells. However, aberrant expression of PD-L1 by tumor cells has been

reported in a number of human malignancies.[42,43,44,45,46,47,48] PD-L1 expressed by tumor cells has been shown to enhance apoptosis of activated tumor-specific T cells in vitro.[39] In renal cell carcinoma, high intratumoral expression levels of PD-L1 is related to tumor aggressiveness.[43] Subjects with high tumor and/or lymphocyte PD-L1 levels are almost 5 times more likely to die from their cancer than subjects exhibiting low levels of PD-L1 expression. It has been reported that PD-L1 and PD-L2 expression may be a significant prognostic marker in post-operative esophageal cancer subjects.[44]

These studies suggest that both normal host mechanisms, i.e., expression of PD-L1 in antigen-presenting cells, as well as tumor specific expression of PD-L1 may limit the antitumor response. Consequently, both PD-L1+ and PD-L1- tumors may be targeted using this approach. Additional background material on the role of the PD-1/PD-L1 pathway may be found in the Investigators Brochure.

1.3. MDX-1106

MDX-1106 is a fully human IgG4 HuMAb that targets the PD-1 cell surface membrane receptor on T lymphocytes and other cells of the immune system.

MDX-1106 is developed by recombinant DNA technology and has been selected for its ability to bind to PD-1 with high affinity and to interfere with the binding of the PD-1 ligands, PD-L1 (B7-H1) and PD-L2 (B7-DC), as well as with the ability to promote IFN-γ secretion and T-cell proliferation. The antibody was initially isolated as an IgG1 and was subsequently engineered to be expressed as an IgG4 with a mutation in the hinge region (S228P) so as to minimize effector function and to provide for increased stability. These properties allow the antibody to activate T-cell responses that have been suppressed through the engagement of the PD-1 molecule with its ligand(s).

1.3.1. Summary of Clinical Safety in Oncology

Initial safety experience of single dose administration of MDX-1106 is available from ongoing Protocol MDX1106-01. Subjects with advanced or refractory malignancies (prostate, colorectal, melanoma, renal cell, and non-small cell lung cancer) received a single dose of MDX-1106 and were monitored for 12 weeks. Subjects without significant disease progression or toxicity during the 12-week observation following the first dose could receive 2 additional doses (at the same dose initially given), administered 4 weeks apart and followed by another 12-week observation before repeating a 2-dose cycle. The dose levels, 0.3, 1.0, 3.0, and 10 mg/kg, were administered to cohorts of 6 subjects with a cohort expansion of an additional 15 subjects at the 10 mg/kg dose level (the highest dose tested; a maximum tolerated dose [MTD] was not defined). All 39

planned subjects for enrollment received at least 1 dose, and no dose-limiting toxicities (DLTs) have occurred in this study.

As of 15 April 2008, 17 subjects have experienced 40 serious adverse events; only 2 of these serious adverse events were considered related to MDX-1106 treatment by the Investigator. Significant adverse events that are likely to be immune-related adverse events (irAEs) and that reflect on safety include polyarticular arthropathy (2 subjects, both low-grade adverse events) and diarrhea/colitis (1 subject, one of the 2 related serious adverse events).

There have been 2 cases of apparent flare of a syndrome of bilateral polyarticular arthropathy in subjects, both had a prior history of similar type syndromes that was unknown to the Investigators at the time of enrollment (1 subject received MDX-1106 3 mg/kg and 1 received 10 mg/kg). These were not high-grade adverse events and promptly responded to moderate corticosteroid treatment. These subjects are ineligible for re-treatment, despite 1 subject having had apparent shrinkage in pulmonary lung cancer lesions and despite the other having had shrinkage in cutaneous melanoma lesions.

A serious adverse event of diarrhea/colitis has been reported in a subject with ocular melanoma. The subject developed colitis more than 5 weeks after receiving his fifth dose of MDX-1106 1 mg/kg over almost 8 months. The colitis has been successfully controlled with steroids and infliximab, administered according to treatment guidelines developed for the management of irAEs observed in the ipilimumab (a fully human monoclonal antibody to CTLA-4/CD152, an immunomodulatory antibody in clinical development by Medarex) development program. The subject continues to maintain a stable response to his disease but is no longer eligible for further treatment with MDX-1106. The Investigator considered the colitis probably related to MDX-1106. This is the first instance of colitis in the MDX-1106 clinical program and it is notable that the colitis did not occur until approximately 9 months after the subject's first dose of MDX-1106. It is also noteworthy that 21 subjects have each received at least a single dose of MDX-1106 10 mg/kg and 3 of these subjects have received 3 doses of 10 mg/kg without such an adverse event. Initial pharmacokinetic data indicate a half-life of approximately 14 days for MDX-1106 and analysis of serum from the index case demonstrated no detectable antibody at the time he received his fourth dose. The potential for additional instances of colitis to emerge will be closely monitored in this study.

Given the expected mechanism of action of MDX-1106, namely disinhibition of cellular immune responses, it is possible that syndromes may develop that are most consistent with an underlying enhanced immune response as the driving factor. Such events may consist of rash, diarrhea and colitis, autoimmune hepatitis, arthritis, glomerulonephritis, or cardiomyopathy. With the limited clinical experience to date, these possibilities are largely based upon preclinical studies in mice

deficient in PD-1, as well as experience with other monoclonal antibodies that act by disinhibiting the immune response.

1.3.2. Rationale for MDX-1106 Dosage Selection in Hepatitis C

The dose levels for this Phase 1 clinical study were selected based on an evaluation of the toxicology data, in vivo efficacy in oncology models, and clinical safety experience to date with administration to oncology subjects. In vitro data on clinical specimens from subjects with infectious diseases shows MDX-1106 to have potency similar to that seen in specimens from subjects with tumors. In general, efficacy is expected to manifest at a dose between 1 and 10 mg/kg.

Preclinical toxicology studies have demonstrated that monkeys tolerated 50 mg/kg on a weekly basis for 5 doses without clinical consequence. Chronic dosing studies in which animals were dosed bi-weekly with up to 50 mg/kg for 3 months have also demonstrated that the treatments were well tolerated without clinical consequence.

MDX-1106 does bind to a cytoplasmic pituitary determinant in normal human tissues as well as in cynomolgus tissues, and no endocrinopathies have emerged in the preclinical toxicology studies, nor in the clinical experience to date. As noted above, there have been no DLTs observed in 39 subjects who have been treated, including 21 subjects dosed at 10 mg/kg. There has been no indication of any cytokine release syndrome, nor any hepatotoxicity (including in 9 subjects with known hepatic metastases).

As a conservative approach, the initial dose chosen for Protocol MDX1106-02 will be a single dose of 0.03 mg/kg. This dose should be sufficient to at least transiently saturate PD-1 receptors, is 10-fold lower than the starting dose used in the oncology study, greater than 100-fold lower than the doses that have been safely reached in the oncology trial, as well as over 100-fold lower than the suggested dose level by NOAEL considerations from preclinical toxicology studies. Additional information on these considerations can be found in the Investigator Brochure. Subsequent dose levels increase by 0.5 log (approximately 3-fold) and are designed to achieve an adequate pharmacokinetic profile within what is expected to be the dynamic range of efficacy of the antibody.

2. STUDY OBJECTIVES

2.1. Primary Objective(s)

The primary objective of the study is to examine the safety, tolerability, and immunogenicity of a single dose of MDX-1106.

2.2. Secondary Objective(s)

The secondary objectives of the study are to determine:

1. The pharmacokinetic profile of a single dose of MDX-1106;
2. Changes in Hepatitis C viral load from baseline, by magnitude and duration, and in relation to administered dose of MDX-1106;
3. The proportion of subjects in each dosing cohort who experience a 0.5-log or greater decline from baseline viral load, that is repeated on at least 2 consecutive measures;
4. Changes in levels of serum cytokines and PBMC reactivity to Hep C viral antigens from baseline and in relation to MDX-1106 dose; and
5. Changes in delayed-type hypersensitivity (DTH) reactivity to a Candida/tetanus antigen skin test, anti-tetanus antibody titers, and PBMC cellular response to common recall antigens in relation to the MDX-1106 dose.

3. OVERVIEW OF STUDY DESIGN

This is a Phase 1, randomized, double-blind, placebo-controlled, multicenter, single-dose, dose-escalation, safety and pharmacokinetic study of MDX-1106. Subjects who failed or relapsed after prior interferon based therapy will be enrolled into 1 of 6 escalating dose cohorts. The first cohort will receive 0.03 mg/kg of MDX-1106 or placebo as an intravenous (i.v.) infusion; subsequent cohorts will receive 0.1, 0.3, 1, 3, or 10 mg/kg of MDX-1106 or placebo. In the first 2 cohorts, no more than 1 subject will receive study drug on the same day. Each cohort will enroll 6 subjects (subjects will be randomized such that 5 subjects will receive MDX-1106 and 1 subject will receive placebo). Each subject will receive a single dose of study drug during the study.

Subjects who withdraw from the study prior to Day 29 for reasons other than safety concerns will be replaced. After the dose escalation cohorts are enrolled (up to 36 subjects) 6 additional interferon experienced subjects will be enrolled in an expansion cohort to obtain further safety information and a preliminary estimate of efficacy. These subjects will be randomly assigned to receive either placebo (1 subject) or MDX-1106 (5 subjects) at or below the maximum tolerated dose (MTD), or the highest tested dose if the MTD is not identified. An additional cohort of 12 subjects who are interferon naïve will be enrolled in parallel with the expansion cohort, and randomly assigned to receive either placebo (2 subjects) or MDX-1106 (10 subjects) at the same dose level as the expansion cohort. The MTD is the highest dose where no subject has experienced a DLT. The MTD may or may not be definitively identified by the initial dose levels.

The study will consist of Screening, Pre-treatment, Treatment, and Follow-up Periods. Beginning with the 0.03 mg/kg dose, MDX-1106 will be administered as an i.v. infusion. Subjects will be monitored for infusion reactions over a 6-hour observation period at the investigative unit (i.e., hospital or clinic unit with resuscitative capabilities) with ready access to an intensive care unit. Subjects who experience an adverse event, including an infusion reaction of Grade 2 during the 6 hour post dose observation period that does not resolve during this time, or any adverse event of Grade ≥ 3 regardless of resolution, will be observed for a minimum of an additional 18 hours or until the adverse event resolves with vital sign measurements every 4 hours and additional evaluations as medically indicated for the management of the adverse event. If there are 2 or more ≥ Grade 2 infusion reaction in a cohort, all subsequent subjects will be pre-medicated with diphenhydramine and acetaminophen. Prior to discharge, subjects will be informed regarding a wide range of symptoms that may be associated with the development of late-occurring hypersensitivities and immune related adverse events (irAEs), (defined as a clinically significant adverse event of any organ that is associated with drug exposure, of otherwise unknown etiology, and consistent with an immune-mediated mechanism), and will be given instruction regarding when they should contact the Investigator and/or report directly to the emergency room.

No 2 subjects will be treated on the same day in the first 2 cohorts. At any dose level, dosing of subjects at the next higher dose level will not be initiated until all 6 subjects in a cohort have been dosed and observed for 4 weeks in the absence of a dose-limiting toxicity (DLT). A DLT is defined as an adverse event or clinically significant laboratory abnormality that emerges within 28 days of dosing with MDX-1106, and whose severity is ≥ Grade 3 and could be considered even remotely related to study treatment. If an event is deemed a DLT, Medarex will unblind the subject to confirm that MDX-1106 was administered. All ≥ Grade 2 infusion reactions that occur during the study will be evaluated as to whether or not the event is a DLT, and reviewed with the Division of Antiviral Products (DAVP) of the Food and Drug Administration (FDA). Events clearly considered unrelated to treatment (e.g., automobile accident, or occurring after administration of placebo) will not be considered a DLT. If 1 DLT is observed in any subject, that cohort will have exceeded the MTD and no further dose escalation will occur. If the previous dose level was well tolerated and no subjects experienced a DLT at that level, an intermediate dose level may be defined by protocol amendment in consultation with the FDA. The MTD will be the highest dose where no subjects have experienced a DLT.

If a DLT occurs in the expansion cohort or interferon naïve cohort, enrollment will be interrupted. The event will be reviewed (which may include unblinding), by Medarex, the Principal Investigator, and DAVP, (with subsequent IRB notification), to determine if enrollment may continue at that dose, or stopped and a new expansion cohort initiated at a lower dose. A

delayed DLT is defined as an event that otherwise meets DLT criteria, but occurs 28 days after MDX-1106 administration. Delayed DLTs will not automatically impact upon dose escalation, but will be collected and evaluated by the Investigators and Medarex on an ongoing basis. If 2 or more such delayed DLTs are noted within a cohort, accrual will be held pending analysis (which may include unblinding), and will be restarted only with Investigator, Medarex, and DAVP approval (with subsequent IRB notification). The potential of a delayed DLT arising after completion of the planned 12-week study period is exceedingly unlikely, as 12 weeks is well in excess of 5 times the anticipated 2-week half-life of MDX-1106.

During the Pre-treatment Period (within 2 to 5 days prior to infusion), placement of DTH skin tests will occur; these skin tests will be read 48 to 72 hours later, just prior to dosing with MDX-1106, and measured again at 24 and 48 hours after dosing. A repeat DTH skin test will be placed on Day 29 and measured 48 to 72 hours later, on Day 31. Blood (for biochemistry and hematology, including liver function tests (LFTs] and HCV viral loads) will be collected at Screening and on Days 1, 2, 3, 8, 15, 22, 29, 43, 57, and 85, and urine samples will be collected at Screening and on Days 1, 3, 8, 29, and 85. Tests for drug and alcohol abuse will be conducted at Screening and prior to study dosing, and at the time of an adverse event if the Investigator considers a causal relationship of the event to drug or alcohol abuse. Immunogenicity testing will be performed on Days 1, 29, and 85; immune safety tests will be performed during the Pre-treatment Period and on Days 8, 29, and 85; and PBMCs for cryopreservation, lymphocytes for phenotype analysis by fluorescent-activated cell sorter (FACS), and serum for cytokines, and for tetanus antibody titer will be collected during the Pre-treatment Period and on Days 1, 2, 3, 8, 15, 29, 57, and 85. Prothrombin time and INR will be measured at Screening and on Days 8, 29, and 85. Subjects will be evaluated for vital signs, adverse events, and concomitant medication use at each visit. Evaluation of safety and tolerability will include assessment of adverse events, toxicity (regardless of Investigator/Medarex attribution to MDX-1106), physical examination including vital sign measurements, electrocardiogram (ECG) measurement, and blood and urine sampling for clinical laboratory parameters.

4. STUDY POPULATION

Up to 42 subjects with active Hepatitis C genotype 1 infection or mixed Hepatitis C genotype (e.g., 1/2, 1/3, 1/4, etc., only if genotype 1 can be shown to be present) who received previous therapy with interferon and ribavirin or peginterferon and ribavirin without a sustained virological response (SVR) or who relapsed following an SVR will be enrolled in the study. An additional 12 subjects who are naïve to an interferon based therapy will also be enrolled in the study. The duration of participation of an individual subject in the study from screening to

completion will be up to 16 weeks, and the overall study is expected to take up to 18 months to reach last subject, last visit.

The specific inclusion and exclusion criteria for enrolling subjects in this study are described in the following sections.

4.1. Inclusion Criteria

As soon as the subject is considered for this study and prior to conducting any study procedures, the subject will have the study explained to them, and will be asked to sign an informed consent form and provide Health Insurance Portability and Accountability Act (HIPAA) authorization. Informed consent form and HIPAA authorization must be obtained prior to any procedures that do not form a part of the subject's normal care.

Subjects must meet the following criteria during the Screening Period (no more than 28 days prior to study drug administration) to be eligible to participate in the study.

1. Adults at least 18 years of age;
2. Infection with Hepatitis C genotype 1 or mixed Hepatitis C genotype (e.g., 1/2, 1/3, 1/4, etc., only if genotype 1 can be shown to be present) documented by previous testing or by new test (TruGene);
3. Chronically infected (at least 6 months since diagnosis) HCV-positive subjects, as documented by serum that tests positive for HCV antibody and an HCV RNA concentration > 100,000 IU/mL within 28 days of the administration of study drug;
4. Asymptomatic or nearly asymptomatic from Hepatitis C. Subjects must be able to carry on normal activity with only minor signs or symptoms of disease;
5. For interferon experienced subjects: previous therapy with interferon and ribavirin or peginterferon and ribavirin without an SVR or relapsed following an SVR. A course of therapy is defined as at least 12 weeks of interferon in doses of 3 million units 3 times weekly or peginterferon in doses of 180 micrograms for peginterferon alfa-2a or 1.5 micrograms/kg for peginterferon alfa 2b once weekly and ribavirin in starting doses of at least 600 mg daily. Subjects who initiated therapy at these doses, but required dose modification/discontinuation due to side effects will also be eligible (see Appendix 1 for categories of prior HCV treatment). For interferon naïve cohort: subjects previously received no interferon based HCV treatment.
6. Liver biopsy within 2 years of the first dose of study drug, documenting changes consistent with Hepatitis C infection. Biopsy must have no evidence of bridging necrosis or cirrhosis;

7. Adequate bone marrow, liver, and renal function as demonstrated by the following laboratory values:

•	Hemoglobin	> 11 g/dL
•	White blood cell count	> 3,000/mm^3
•	Neutrophil count	> 1,500/mm^3
•	Platelet count	>125,000/mm^3
•	Total bilirubin	< 2 mg/dL
•	Direct bilirubin	< 1.5 times the upper limit of normal (ULN)
•	Albumin	≥ 2.8 g/dL
•	ALT	< 5 times ULN
•	Serum creatinine	< 1.5 times ULN
•	Prothrombin time (PT)/ International normalized ratio (INR)	≤ 1.7

8. Blood pressure within normal limits (systolic: 90 to 140 mmHg, diastolic: 50 to 90 mmHg) and heart rate between 40 and 100 beats per minute. This includes subjects who have achieved these levels while on a regular antihypertensive regimen for ≥ 3 months;

9. Electrocardiogram with no clinically significant abnormalities recorded at the Screening Visit. PR interval within 120 and 200 ms, QRS interval <120 ms, and QTc interval ≤ 440 ms;

10. Chest X-ray consistent with “no active disease”;

11. No history of cirrhosis;

12. α-fetoprotein < 50 μg/L (when between the ULN and 50 μg/L, ultrasonographic exclusion of hepatocellular carcinoma [HCC] is needed);

13. Women must meet 1 of the following criteria: post-menopausal for at least 2 years (without menses for 24 consecutive months); surgically incapable of bearing children (have had a hysterectomy or bilateral oophorectomy); or utilizing a reliable form of contraception, and must agree to continue such use for 70 days after their dose of study drug; and

14. Men must agree to the use of male contraception during the study and for at least 180 days after their dose of study drug.

4.2. Exclusion Criteria

Subjects who fulfill any of the following criteria at Screening will not be eligible for admission into the study:

1. Acute Hepatitis C infection (within 6 months of diagnosis prior to planned infusion);

2. Treatment with any IFN-based therapies and/or antiviral therapies within 28 days of study drug administration;
3. Decompensated liver disease (presence of encephalopathy, ascites, bilirubin > 2, albumin < 2.8, prothrombin time > 1.7 INR, or history of portal hypertension especially those with varices or variceal bleeding);
4. Subjects with a history of myocardial infarction within the previous 5 years or those with NYHA functional class I or worse;
5. History of receipt of any disease modifying anti-rheumatoid arthritis drugs (DMARDs) for 14 days or more including but not limited to auranofin, azathioprine, chlorambucil, cyclophosphamide, cyclosporine, gold sodium thiomalate, hydroxychloroquine sulfate, leflunomide, methotrexate, minocycline, mycophenolate mofetil, and sulfasalazine;
6. Pregnant or nursing;
7. Use of immunosuppressive doses of steroids or any immunosuppressive therapies within 28 days before study drug administration; inhaled and topical corticosteroids are permitted;
8. History of hypersensitivity to any biological agent or antibody;
9. History of significant gastritis (confirmed by endoscopy and resistant to antibiotics/proton pump and histamine receptor inhibitors or associated with hemorrhage);
10. Receipt of any immunoglobulin within 6 months prior to study drug administration;
11. Receipt of any prescription or over the counter drugs within 14 days before study drug administration that may have the potential to cause hepatotoxicity;
12. History of major surgery requiring general or spinal anesthesia within 30 days before study drug administration;
13. Receipt of any investigational drug therapy within 28 days (or less than 5 half-lives) prior to study drug administration; or
14. Any serious medical or psychiatric conditions which, in the opinion of the Investigator, may compromise the safety or compliance of the subject or would preclude the subject from successful completion of the study. Conditions include, but are not limited to the following:
    - HIV-1 or HIV-2;
    - Hepatitis B (positive Hepatitis B surface antigen [HbsAg1]);

- Latent tuberculosis infection defined as a new PPD conversion within the previous 5 years (and lack of evidence for active pulmonary disease) that has not been treated with standard prophylactic therapy (e.g., isoniazid);
- History of acquired or inherited immunodeficiency or autoimmune disease either documented or anecdotal (see Appendix 4 for a non-exhaustive list of exclusionary autoimmune diseases). Exceptions include subjects with a history of atopic disease or childhood arthralgia where the clinical suspicion of autoimmune disease is low;
- Cancer (active tumors in the last 5 years, except for resected non-melanoma skin lesions);
- Bleeding disorders
- Signs and symptoms of an active infection (e.g., upper respiratory tract infection, urinary tract infection, or active herpes simplex infection);
- Alcohol or drug misuse within 6 months of the Screening Period (misuse in this period is defined as more than 1 bout of intoxication or continued use of alcohol when contraindicated by known adverse effects on LFTs). Subjects with a documented addiction-free period of at least 6 months may be enrolled in the study providing that, in the clinical judgment of the Investigator, they are not at risk for relapse. Subjects in a supervised methadone treatment program may also be enrolled.

5. RANDOMIZATION AND BLINDING

The study center will contact Medarex once a subject is determined to be eligible for enrollment for treatment assignment. Medarex will notify the pharmacist regarding whether the subject has been randomized to receive MDX-1106 or placebo, using tables generated by the Medarex Biostatistics group. Subjects will be assigned to the next available dose level, as determined by Medarex.

With the exception of the site pharmacist (site personnel preparing the infusion), the remaining site staff must remain blinded to study drug assignment and care should be exercised so that the blind is not broken. Medarex will not be blinded. In the event of a medical emergency where knowledge of study drug assignment may influence treatment decisions, or for determining whether an apparent DLT has occurred in a subject that received MDX-1106, the blind may be broken. If such an emergency situation arises, the Investigator should first attempt to contact the Medarex medical monitor or designee. If immediate unblinding is required for an emergency medical situation then the Investigator should contact the pharmacist for the unblinding information. The site must inform Medarex or designee within 24 hours of unblinding a subject.

6. ASSIGNMENT TO STUDY

Screening numbers will be assigned by the Principal Investigator/designee at each study site as soon as the subject has signed the informed consent and HIPAA authorization. Subjects that are screened and do not meet all entry criteria will be entered into a screening log. Once assigned, numbers for any screening failures, non-treated, non-evaluable, or discontinued subjects will not be re-used. Subjects will be assigned to receive either MDX-1106 or placebo as described in Section 5.

7. DOSAGE AND ADMINISTRATION

7.1. Dosing Overview

MDX-1106 is provided at a concentration of 10 mg/mL in vials containing 5 or 10 mL. The total dose to be administered will be diluted to a total volume of 60 mL in sterile normal saline (0.9% sodium chloride). In cases where the total volume is more than 60 mL, no dilution is necessary. The dose (in infusion bags) will be filtered through a sterile 0.20 μM-in-line filter prior to administration.

For subjects in the 2 lowest dose cohorts, MDX-1106 will be prepared by diluting sufficient material for a 0.3 mg/kg dose in 60 mL. For the lowest dose cohort (0.03 mg/kg), only 6 mL of this dilution will be administered by infusion over 15 minutes. For the 0.1 mg/kg cohort, only 20 mL will be infused over 30 minutes. For both of these cohorts, the required volume will be transferred to a soluset for the infusion. Subsequent dose cohorts will be administered MDX-1106 at doses of 0.3, 1, 3, 10 mg/kg over a 60-minute period; infusions will be controlled by a volumetric pump with a saline flush at the end of the infusion for all cohorts.

Study Drug Preparation

As MDX-1106 is stored at refrigerated temperatures (2° to 8°C), allow the appropriate number of vials of MDX-1106 to stand at room temperature for approximately 5 minutes.

1. Ensure that the MDX-1106 solution is clear, colorless, and essentially free from particulate matter on visual inspection. If multiple vials are needed for a subject, it is important to use a separate sterile syringe and needle for each vial to prevent problems such as dulling of needle tip, stopper coring, repeated friction of plunger against syringe barrel wall and so on.
2. Aseptically withdraw the required volume of MDX-1106 solution into a syringe. Insert the needle at an angle into the MDX-1106 vial by placing the needle bevel side down against the glass, with the tip touching the neck of the vial. The initial solution concentration is

10 mg/mL. (Note: A sufficient excess of MDX-1106 is incorporated into each vial to account for withdrawal losses).

3. Prepare the MDX-1106 solution for infusion per the example provided below:

   Total dose should be calculated as follows:

   Subject body weight in kg x 3 mg (for the 3 mg/kg cohort) = total dose, mg

   For example, a subject with a body weight of 70 kg and assigned to the 3.0 mg/kg cohort would be administered 210 mg of MDX-1106 (70 kg x 3.0 mg/kg = 210 mg). Twenty-one mL of MDX-1106 and 39 mL of normal saline would be mixed in the i.v. bag and the solution would be infused over 60 minutes.

4. Mix by GENTLY inverting several times. DO NOT shake.
5. Visually inspect the final solution. If the infusion is not clear or the contents appear to contain precipitate, the solution should be discarded and documented on the Drug Accountability Log.
6. Do not enter into each vial more than once. Any partial vials should be safely discarded per the sites SOPs and should not be re-used.
7. Do not prepare MDX-1106 for infusion in glass syringes.

MDX-1106 and placebo should be administered under the supervision of a physician experienced in the use of i.v. agents. Study drug is not to be administered as an i.v. push or bolus injection. The time MDX-1106 was prepared must be documented and recorded on the Drug Accountability Log and i.v. bag label.

Placebo

Placebo, normal sterile saline (0.9% sodium chloride), will be prepared according to the manufacturer's instruction, and is to be provided by the site pharmacy. The volume of placebo will be the same as the corresponding MDX-1106 dose level.

Study Drug Administration

MDX-1106 will be administered at a dosage of 0.03, 0.1, 0.3, 1, 3, 10 mg/kg/dose, administered as an i.v. infusion controlled by a volumetric pump over 15, 30, or 60 minutes (as described above), with a saline flush at the end of the infusion. Placebo will be administered using the rate corresponding to the MDX-1106 dose level.

- Attach the i.v. bag containing the MDX-1106 (or placebo) drug solution to the infusion set, 0.2 μM in-line filter, and infusion pump.

- The infusion rate of the infusion pump should be adjusted to allow for a total infusion time of 15, 30, or 60 minutes (as described above).
- At the end of the infusion period, flush the line with normal saline.

7.2. Packaging and Labeling

MDX-1106 will be supplied at a concentration of 10 mg/mL in vials containing 5 or 10 mL. MDX-1106 vials are labeled and supplied in a carton. Details of the packaging and labeling of clinical supplies may be found in the Pharmacy Manual.

7.3. Storage

MDX-1106 vials must be stored at a temperature of 2°C to 8°C and should be protected from light and not stored in a glass front refrigerator (if no other refrigerator is available, samples should be stored in the carton in a brown paper bag). Recommended safety measures for preparation and handling of MDX-1106 include laboratory coats and gloves. Note: once MDX-1106 has been prepared for administration, the total storage time (combination of refrigeration and room temperature) is not to exceed 24 hours. Stability data for MDX-1106 supports 6 hours at room temperature/under room light and 18 hours at 2°C to 8°C in the refrigerator following dilution and transfer to the i.v. bag. Care must be taken to assure sterility of the prepared solution as the product does not contain any anti-microbial preservative or bacteriostatic agent. No incompatibilities between MDX-1106 and polyolefin bags have been observed.

Placebo, sterile normal saline, should be stored at the pharmacy according to the manufacturer's instructions.

7.4. Inventory

Medarex, Inc. is the manufacturer and provider of the study drug supply. All study drug(s) will be supplied to the Investigator by Medarex or its designee. Study drug supplies must be kept in an appropriate, secure locked area and stored in accordance with the conditions specified on the labels. An accurate record of the date and amount of study drug dispensed to each subject must be available for inspection at any time.

7.5. Dispensing Supplies

Clinical supplies for the Treatment Period will be provided to the site from open-label stock. The investigative site must maintain an accurate record of dispensing the study drug in a Drug Accountability Log, a copy of which must be given to Medarex at the end of the study. The Drug Accountability Log will record the study drug used, dosages, and the time administered. The

Drug Accountability Log will be reviewed and noted by the field monitor during site visits and at the completion of the study.

The placebo, sterile normal saline (0.9% sodium chloride) is to be provided by the investigative site. The manufacturer and lot numbers of the placebo should also be recorded on a Drug Accountability log.

All study drug(s) are to be used only for this protocol and not for any other purpose. All study drug(s), remaining at the time of closure of the study site will be destroyed by the site, in accordance with the site's Standard Operating Procedures (SOPs).

7.6. Ordering Study Medication

Clinical supplies may be requested by completing a Request Form and faxing it to the Clinical Operations Contact at Medarex.

8. MDX-1106: TOXICITY, AND MANAGEMENT

8.1. Dose Escalation

Initially, cohorts of 6 subjects (5 MDX-1106; 1 placebo) will be enrolled in each dose cohort. An additional 12 subjects will be enrolled in the expansion cohort (2 placebo, 10 at or below the MDX-1106 MTD or the highest tested dose if the MTD is not identified). Subjects in the first dose cohort will be administered MDX-1106 at a dose of 0.03 mg/kg; subsequent cohorts of subjects will be administered MDX-1106 at escalating dose levels of 0.1, 0.3, 1, 3, and 10 mg/kg. No 2 subjects will be treated on the same day in the first 2 cohorts. At any dose level, dosing of subjects at the next higher dose level will not be initiated until all 6 subjects in a cohort have been dosed and observed for 4 weeks in the absence of a DLT (see definition in the following section). If the previous dose level was well tolerated and no subjects experienced a DLT at that level, an intermediate dose level may be defined by protocol amendment.

The MTD dose will be the highest dose where no subjects have experienced a DLT. Safety will be confirmed by enrolling 1 additional subject for treatment with placebo and an additional 5 subjects for treatment with a MDX-1106 dose at or below the MTD or the highest dose tested. Safety will also be confirmed for subjects naïve to interferon based therapy by enrolling an additional 2 subjects for treatment with placebo and an additional 10 subjects for treatment with MDX-1106 at the same dose as that chosen for the interferon experienced expansion cohort.

8.2. Dose-limiting Toxicity

A DLT is defined as an adverse event or clinically significant laboratory abnormality that emerges within 28 days of dosing, and whose severity is $\geq$ Grade 3 and could be considered even

remotely related to study treatment. If an event is deemed a DLT, Medarex will unblind the subject to confirm that MDX-1106 was administered. All ≥ Grade 2 infusion reactions will be evaluated as to whether or not the event is a DLT. Events clearly considered unrelated to treatment (e.g., automobile accident, or occurring after administration of placebo) will not be considered a DLT.

All adverse events that meet DLT criteria (see Appendix 3 for Division of Microbiology and Infectious Disease [DMID] toxicity criteria), as well as any unexpected toxicity, must be reported to Medarex within 24 hours.

8.3. Stopping Rules for Dose-limiting Toxicity and Delayed Dose-limiting Toxicity

The dose-escalation schema will proceed based upon DLTs experienced within the Treatment and Follow-up Periods. If a DLT occurs at a protocol specified dose level and if the previous dose level was well tolerated and no subjects experienced a DLT at that level, an intermediate dose level may be defined by protocol amendment.

A delayed DLT is defined as an event that otherwise meets DLT criteria, but occurs after 28 days from MDX-1106 or placebo administration. Delayed DLTs will not automatically impact upon dose escalation, but will be collected and evaluated by the Investigators and Medarex on an ongoing basis. If 2 or more such delayed DLTs are noted within a cohort, accrual will be held pending analysis (which may include unblinding), and will be restarted only with Investigator, Medarex, and DAVP approval (with subsequent IRB notification).

Considering the expected half-life of the molecule, DLTs are most likely to occur during treatment or within the 28 days following treatment. Delayed toxicity will likely represent drug effect and not dose effect. Therefore, assessment of delayed toxicity will not directly impact on dose escalation, but will be assessed separately for dose limitation. Subject participation in this protocol is planned for 85 days. Given the expected half-life of the antibody to be on the order of 14 days, this period of time is well in excess of 5 half-lives. It is therefore exceedingly unlikely that any delayed toxicity will emerge after study completion.

8.4. Stopping Rules for the Expansion Cohorts

If a DLT occurs in the expansion cohorts, enrollment will be paused. The event will be reviewed (which may include unblinding), by Medarex, the Investigator, and DAVP (with subsequent IRB notification), to determine if enrollment may continue at that dose, or stopped and a new expansion cohort initiated at a lower dose.

8.5. Infusion Reactions

It is likely that most infusion-related events will occur within the first 24 hours after beginning the infusion and may be treated by slowing or interruption of the infusion or with supportive treatment.

Since MDX-1106 contains only human protein sequences, it is unlikely to be immunogenic and induce infusion or hypersensitivity reactions. Since this antibody specifically binds to PD-1, this makes it less likely that such a reaction would occur. However, if such a reaction were to occur, it might manifest with fever, chills, rigors, headache, rash, pruritus, arthralgias, hypo- or hypertension, bronchospasm, or other symptoms. All ≥ Grade 2 infusion reactions will be evaluated as to whether or not the event is a DLT.

Prophylactic premedication **WILL NOT** be given initially. Should 2 or more subjects develop ≥ Grade 2 symptoms within a cohort, the following prophylactic pre-medications are recommended for future infusions in subsequent subjects: diphenhydramine 50 mg (or equivalent) and/or 500 to 750 mg paracetamol (acetaminophen) at least 30 minutes prior to MDX-1106/placebo administrations.

Infusion reactions should be graded according to NCI Common Terminology Criteria for Adverse Events (Version 3.0) guidelines, http://ctep.cancer.gov (no specific guidelines regarding infusion reactions are included in the DMID criteria). Treatment recommendations are provided below and may be modified based on local treatment standards and guidelines, as appropriate:

For Grade 1 symptoms: (Mild reaction; infusion interruption not indicated; intervention not indicated)

- Remain at bedside and monitor subject until recovery from symptoms.

For Grade 2 symptoms: (Moderate reaction, requires therapy or infusion interruption but responds promptly to symptomatic treatment [e.g., antihistamines, NSAIDS, narcotics, corticosteroids, i.v. fluids]; prophylactic medications indicated for ≤ 24 hours)

- Stop the MDX-1106 infusion, begin an i.v. infusion of normal saline, and treat the subject with diphenhydramine 50 mg i.v. (or equivalent) and/or 500 to 750 mg paracetamol/acetaminophen; remain at bedside and monitor subject until resolution of symptoms. Corticosteroid therapy may also be administered as appropriate. If the infusion is interrupted, then restart the infusion at 50% of the original infusion rate when symptoms resolve; if no further complications ensue after 30 minutes, the rate may be increased to 100% of the original infusion rate. Monitor subject closely. If symptoms recur then no further MDX-1106 will be administered at that visit. The amount of study drug infused

must be recorded on the case report form (CRF). Subjects who experience an adverse event, including an infusion reaction of Grade 2, during the 6 hour observation period that does not resolve during this time will be observed for an additional 18 additional hours or until the adverse event resolves with vital sign measurements every 4 hours and additional evaluations as medically indicated for the management of the adverse event.

For Grade 3 or Grade 4 symptoms: (Severe reaction, Grade 3: prolonged [i.e., not rapidly responsive to symptomatic medication and/or brief interruption of infusion]; recurrence of symptoms following initial improvement; hospitalization indicated for other clinical sequelae [e.g., renal impairment, pulmonary infiltrates]. Grade 4: life-threatening; pressor or ventilatory support indicated).

- Immediately discontinue infusion of MDX-1106. Begin an i.v. infusion of normal saline, and treat the subject as follows: Recommend bronchodilators, epinephrine 0.2 to 1 mg of a 1:1,000 solution for subcutaneous administration or 0.1 to 0.25 mg of a 1:10,000 solution injected slowly for i.v. administration, and/or diphenhydramine 50 mg i.v. with methylprednisolone 100 mg i.v. (or equivalent), as needed. Subject should be monitored until the Investigator is comfortable that the symptoms will not recur. MDX-1106 will be permanently discontinued. Investigators should follow their institutional guidelines for the treatment of anaphylaxis. Subjects who experience an adverse event, including an infusion reaction of Grade ≥ 3, regardless of resolution, will be observed for 18 additional hours or until the adverse event resolves with vital sign measurements every 4 hours and additional evaluations as medically indicated for the management of the adverse event.

In the case of late-occurring hypersensitivity symptoms (e.g., appearance of a localized or generalized pruritus within 1 week after treatment), symptomatic treatment may be given (e.g., oral antihistamine, or corticosteroids).

8.6. Possible Toxicities

There is not enough clinical experience with MDX-1106 to define expected toxicities. Possible toxicities could affect the immune system, hematologic, cardiovascular, hepatic, musculoskeletal, and other systems, and may include the following:

- **Allergic reaction/hypersensitivity:** Fever, chills, shakes, itching, rash, hyper- or hypotension, difficulty breathing. It is likely that most infusion-related events will occur within the first 24 hours after beginning the infusion and may be treated by slowing or interruption of the infusion or with supportive treatment, as indicated.
- **Widespread immune activation/cytokine storm:** Cytokine storm events may initially look like allergic reaction/hypersensitivity but are distinguished by more sustained and

profound hemodynamic disturbances related to the widespread release of cytokines, such as IL-1 and TNF. Symptoms may include fever, myalgia, change in mental status, hypotension, pulmonary infiltrates, metabolic acidosis, and acute renal failure. Cytokine storm has been observed with an agonistic anti-CD28 antibody (TGN1412) but is not expected with MDX-1106 and has not been seen in preclinical testing nor in human subjects with cancer treated to date.

- **Gastrointestinal system:** Colitis, characterized by new onset of diarrhea, which may be accompanied by abdominal pain and or GI bleeding. Events of Grade 3 or 4 diarrhea, as well as Grade 2 diarrhea with blood in the stool should prompt an investigation for colitis. All events of colitis ≧ Grade 2 are deemed to be of special interest, and should be reported using the serious adverse event reporting procedures, even if the event itself is not deemed as serious. A diarrhea management algorithm is provided in the Investigator Brochure for treatment of this adverse event, should it arise. Any Grade 2 event of colitis (per CTCAE) that also results in additional medical requirements, such as high dose steroids, blood transfusion or intravenous hyperalimentation, will be defined as a Grade 3 adverse event. Subjects are to be carefully monitored until recovery of the colitis to ≤ Grade 1.
- **Immune suppression:** Subjects should be monitored for signs of new infection or return of a previous infection with rash, fever, chills, other localizing symptoms, or sepsis that could require antibiotics either as prevention or treatment.
- **Musculoskeletal system:** Muscle or joint aches or swelling, weakness.
- **Blood:** A decrease in blood components (platelets, white or red blood cells) that could lead to infection, bleeding, or anemia.
- **Liver:** Liver cell damage/inflammation with increased liver cell enzymes and decreased liver function.
- **Skin:** The most likely adverse events are rash and pruritus, which generally resolve when drug therapy is discontinued.
- **Immune-related adverse events (irAEs):** An immune-related adverse event (irAE) is defined as a clinically significant adverse event of any organ that is associated with drug exposure, of otherwise unknown etiology, and consistent with an immune-mediated mechanism. Serological, immunological, and histological (biopsy) data should be used to support an irAE diagnosis. Appropriate efforts should be made to rule out neoplastic, infectious, metabolic, toxin, or other etiologic causes of the adverse event before assessing it as an irAE. Given the expected mechanism of action of MDX-1106, namely disinhibition of cellular immune responses, it is possible that syndromes may develop that are most consistent with an underlying enhanced immune response as the driving factor. Such events

may consist of persistent rash, diarrhea and colitis, autoimmune hepatitis, arthritis, glomerulonephritis, or cardiomyopathy. The spectrum of irAEs is currently hypothetical, as very few human subjects have been treated to date, and are based upon preclinical studies in mice deficient in PD-1, as well as experience with other monoclonal antibodies that act by disinhibiting the immune response. Such irAEs may resolve with time, or may require institution of counteracting immunosuppressive therapies.

Medarex has observed irAEs in another development program with an immunostimulatory antibody, ipilimumab. Ipilimumab-induced irAEs are typically low grade and self limited, more often occur after multiple doses, and most frequently involve the gastrointestinal tract (diarrhea/colitis), skin (rashes), liver (hepatitis), and endocrine systems (a variety of endocrinopathies). Management algorithms for high grade irAEs have been established, where timely application of defined immunosuppressive regimens appear to be effective in limiting the morbidity and mortality from such events without compromising therapeutic efficacy. Additional clinical experience will be required to define the spectrum of irAE-like events that may emerge in the MDX-1106 program, and these algorithms are useful guides towards establishing an effective management approach as experience accumulates.

8.6.1. Hepatotoxicity Evaluation

One of the potential concerns resulting from disinhibition of HCV-specific T cell responses by virtue of PD-1 blockade with MDX-1106 is that these T cells may attack HCV-infected hepatocytes and result in significant hepatoxicity, either by directly killing the infected hepatocytes or via bystander effects. As noted in the introduction, most animal models suggest that it is in fact the failure of antigen-specific T cells to adequately control HCV replication that results in the recruitment of additional non-specific T cells and hepatocyte injury via bystander effects.[1] Thus, it is hoped that activation of HCV T-cell responses may result in decreased viral loads without additional or excess hepatocyte injury. Nonetheless, careful monitoring for hepatotoxicity is indicated.

In the absence of actual clinical experience with MDX-1106 in HCV subjects, it is premature to define a precise treatment algorithm for the management of hepatoxicity for subjects in this trial. Careful single dose administration and follow-up prior to dose escalation should allow for early detection of potential hepatotoxicity signals before they become serious. The information provided below is to support decision-making, and is not a prescribed treatment algorithm.

Should hepatoxicity develop after treatment with MDX-1106, subjects will need to be followed with heightened surveillance of their clinical status, as well as undergo an evaluation as to the apparent cause of the hepatoxicity. Hepatoxicity in this setting may in principle be mediated by

viral lytic effects, direct hepatocyte toxicity, or via autoimmune mechanisms. HCV is not considered to be a lytic virus for hepatocytes[1], but viral load should be carefully followed as well. MDX-1106 has not shown any evidence of direct hepatocyte toxicity in preclinical models or in the oncology subjects treated to date, but this should be considered. All other potentially hepatoxic concomitant medications should also be carefully reviewed. The presence of other immune related phenomena, such as rash, or new elevations in the titers of auto-antibodies such as ANA or ASM may be used to support the conclusion that the hepatoxicity is autoimmune in nature. Given the intended mechanism of action of MDX-1106, it seems most likely that autoimmunity would be the leading suspect as a cause for hepatotoxicity, but this should not be assumed.

Active surveillance and management plans will include:

- Close follow up of LFTs, including ALT, AST, and bilirubin (hepatocellular injury), as well as PT, albumin (synthetic function):
  - Increases of 3X above baseline LFT values will require confirmation within 24 hours (via non-scheduled visits if necessary).
  - Sustained elevations will trigger heightened monitoring with frequent LFTs (daily to q72 hours as clinically required) until resolution to 1.5 X baseline or less.
  - Failure of the LFT elevations to resolve on their own within 7 days, or worsening of LFTs in this time frame and the presence of clinical symptoms (e.g., sleep disturbance, fatigue, nausea) will trigger consideration of medical interventions, to be agreed upon by the principal investigator and the Medarex medical monitor and may include:
    a. Continued observation and evaluation of likely mechanism of hepatotoxicity – change in viral loads, evaluation of potential hepatoxic con-meds, unscheduled ANA and ASM titers to compare to baseline and previous scheduled assessments. A liver biopsy may be considered by the Investigator to confirm hepatoxic vs autoimmune mediated mechanisms.
    b. For clinically significant autoimmune hepatitis (presence of symptoms and LFTs > 5X baseline), consider introduction of a course of pulse steroids to be tapered over 7 weeks and to include 60 mg/day of prednisone x 2 weeks, 40 mg/day x 2 weeks, 20 mg/day x 2 weeks, and 10 mg/day x 1 week. The decision to treat the subject with steroids should be governed primarily by clinical assessment as well as liver function abnormalities. While a decision to wait until the emergence of overt clinical symptoms of hepatic dysfunction may be preferred to treatment of biochemical abnormalities alone, this should be governed by clinical judgment with regard to the apparent extent and rate of change of the LFT functions, and

tempered by the consideration that an earlier intervention may have a better chance of success if the subject is clinically deteriorating. See next section (8.6.2) on additional considerations for steroid use.

c. Additional interventions that may be considered are the introduction of other immunosuppressive agents (such as mycophenylate or cyclosporine) for autoimmune hepatitis. These immunosuppressive agents may also be used in combination with active antiviral treatment with interferon and/or ribavirin for rising HCV viral loads.

8.6.2. Rationale for Consideration of Corticosteroid Treatment of Serious Immune Related Adverse Events (e.g., Hepatitis, or other) in Subjects with Active Hepatitis C Infection

The literature on the use of corticosteroid therapy in subjects with chronic HCV is limited. Corticosteroid therapy in the setting of acute Hepatitis B is contraindicated, and on the basis of general principles, use of an inhibitor of cellular immunity is not the standard of care for HCV infection. There are several reports however, on the effects of such therapy on HCV infection itself, as well as on the utility of steroid therapy for the autoimmune phenomena that may rarely accompany chronic HCV infection.

The 7-week steroid treatment regimen proposed above as a rescue for clinically significant autoimmune hepatitis due to MDX-1106 has been previously shown to reduce elevations in ALT and AST, albeit transiently increasing HCV viral load, and upon steroid discontinuation resulted in recovery of baseline LFTs and viral load without apparent long-term deterioration in the course of HCV disease.[49] This study concluded that the predominant mechanism of LFT abnormalities was immune mediated, rather than virally mediated, as LFTs improved on steroids even in the face of a ten fold increase in HCV titer. Therefore, it is proposed that such a regimen may suppress potential MDX-1106-induced autoimmune hepatotoxicity for a sufficient period of time for the antibody to be substantially cleared from circulation.

Other studies also demonstrate that brief courses of steroids in HCV subjects are well tolerated, and that long term courses result in neither clearance of disease nor significant worsening.[50,51,52] More recent studies have also found evidence for efficacy of corticosteroid therapy in the treatment of autoimmune phenomena in subjects with chronic HCV.[53, 54]

9. COMPLIANCE

The designated study personnel at the Investigator's site will maintain a log of all study drugs (including manufacturing information and lot number for the normal saline used as placebo) received and returned. Drug supplies will be inventoried and accounted for throughout the study.

All clinical supplies will be stored in locked facilities. All study drug(s) remaining at the time of closure of the study site will be destroyed by the site, in accordance with the site's Standard Operating Procedures (SOPs). An accountability log will be kept, indicating disposition of study drug; this log will be provided to Medarex of designee upon the completion of the study, and as appropriate during the course of the study.

10. CONCOMITANT THERAPY

All medications taken within 6 weeks prior to the administration of MDX-1106, and all concomitant therapy administered during the study and for 70 days following the dose of study drug should the subject withdraw prior to Day 85 are to be recorded on the relevant CRFs (or electronically if electronic data capture [EDC] is used) along with the reason for and details of therapy use.

a. Subjects should avoid the regular use of acetaminophen during the study; occasional use in amounts up to 2 g/day is allowed.

b. Treatment with any IFN-based therapies and/or antiviral therapies within 28 days of study drug administration and during the study is prohibited.

c. History of treatment with any disease modifying anti-rheumatoid arthritis drugs (DMARDs) for 14 days or more including but not limited to auranofin, azathioprine, chlorambucil, cyclophosphamide, cyclosporine, gold sodium thiomalate, hydroxychloroquine sulfate, leflunomide, methotrexate, minocycline, mycophenolate mofetil, and sulfasalazine. Use of these medications during the study is prohibited.

d. Use of immunosuppressive doses of steroids or any antimetabolite therapies within 28 days of entry into the study and during the study is prohibited unless required to treat clinically significant autoimmune reactions such as autoimmune hepatitis. Inhaled and topical corticosteroids are permitted.

e. Use of any immunoglobulin within 6 months prior to study drug administration and during the study is prohibited.

f. Use of antihypertensive medications during the study is permitted only if such therapy was initiated and maintained for ≥3 months prior to the Screening Visit, or indicated because of a significant change in medical condition.

All subjects should be maintained on the same concomitant medications throughout the study period, as medically feasible. Any new concomitant medications prescribed for the subject or changes to dosing/schedule of concomitant medications should be recorded on the appropriate CRF page (or electronically if EDC is used).

11. STUDY EVALUATIONS

11.1. Study Procedures by Visit

11.1.1. Overview

The study is divided into periods with associated evaluations and procedures that must be performed at specific time points, as described in the following sections. The Time and Events Schedule (Table 1) summarizes the frequency and timing of efficacy, safety, and other study procedures.

As soon as the subject is considered for this study and prior to performing any study procedures, the subject will have the nature of the study explained to him/her, and will be asked to give written informed consent and HIPAA authorization. Informed consent/HIPAA authorization must be obtained prior to any procedures that do not form a part of the subject's normal care.

All subjects (withdrawn or completed) will have final evaluations and procedures performed (Off Study Visit).

11.1.2. Screening Period

Subjects will be evaluated for entry criteria during the Screening Period within 28 days prior to administration of study drug.

Study Day -28 through Day -6

The following procedures and evaluations will be completed for each subject prior to Day -5 and prior to inclusion in the study:

- Inclusion/exclusion criteria.
- Demographics and medical history (to include collection of prior medication [specifically medications for Hepatitis C], medications administered to the subject during the Screening Period, and prior and concurrent medical conditions). Clinical events occurring after signing informed consent/HIPAA authorization, but prior to study drug administration are to be recorded on the Medical History/Current Medical Conditions CRF.
- Vital sign measurements (including weight, height, temperature, pulse, respiration, and semi-recumbent systolic and diastolic blood pressure).
- Complete physical examination (including examination of skin; head; eyes, ears, nose, and throat [HEENT]; neck, joints; lungs; heart, abdomen (including liver and spleen); stool for occult blood, lymph nodes; and extremities. Neurological examination should include assessment of cranial nerves, motor, sensory, and deep tendon reflexes.
- Chest radiograph.

- Electrocardiogram (ECG).
- Liver biopsy (if not performed within the past 2 years). A separate informed consent form must be signed by the subject before the biopsy is performed.
- Clinical laboratory tests (central laboratory): Hematology: Hemoglobin, hematocrit, CBC counts with differential (including absolute lymphocyte count) and direct platelet count.
- Biochemistry:
  - Albumin
  - Serum alkaline phosphatase
  - SGOT (AST)
  - SGPT (ALT)
  - Bilirubin (direct and total)
  - Calcium
  - GGT
  - Creatinine
  - Glucose
  - Lactate dehydrogenase (LDH)
  - Total protein
  - Urea nitrogen (BUN)
  - Uric acid
  - Creatinine kinase
  - Electrolytes (including sodium, potassium, chloride, and bicarbonate)
- Urinalysis:
  - Gross examination including specific gravity, protein, glucose, and blood.
  - Microscopic examination including WBC/HPF, RBC/HPF, and any additional findings.
- α-fetoprotein.
- Viral marker testing including HIV-1 and HIV-2 antibody and Hepatitis B surface antigen.
- Prothrombin time and INR.
- Drug and alcohol abuse screens (urine toxicology screen, blood alcohol).
- HCV genotype 1 assay (TruGene), unless previously determined by this or another documented assay.

- HCV viral load, determined by a quantitative RNA PCR-based assay (Cobas TaqMan HCV test [lower limit of detection = ~10 IU/mL]).
- Concomitant medications and prior medications taken within 6 weeks of study drug administration.
- Collection of baseline signs and symptoms

11.1.3. Pre-treatment Period

Study Day -5 through Day -2

The following procedures and evaluations will be completed for each subject between Day -5 through Day -2 and prior to inclusion in the study:

- Serum ß-HCG pregnancy test (to be taken within 5 days prior to the first infusion; for all women of childbearing potential; serum pregnancy test must be negative to continue). Women who have had a hysterectomy or bilateral oophorectomy, or who are postmenopausal (> 24 months since last menses), or who are > 60 years of age do not require a pregnancy test.
- Vital sign measurements (as outlined in Section 11.1.2; repeat height measurement is not required).
- Abbreviated physical examination: at minimum to include eyes, throat, neck/lymph nodes, chest, abdomen and skin, and others as directed by symptoms. Abnormal findings from screening exam considered of potential significance to course of Hepatitis C disease should be explicitly followed.
- Clinical laboratory tests (as outlined in Section 11.1.2).
- Immune safety assays:
  - Rheumatoid factor (RF)
  - Thyroid-stimulating hormone (TSH)
  - T3
  - Free T4 level
  - C-reactive protein (CRP)
  - Antinuclear antibody (ANA) titer and pattern
  - Anti-smooth muscle (ASM) antibody titer
  - Cardiac Troponin I level

If, during the course of the study, a 4-fold increase from baseline in RF, ASM or ANA or abnormal levels of TSH, T3, T4, or CRP are observed, the following tests will be performed on stored samples at a later date:

- Anti-DNA antibody
- Anti-SSA antibody (Ro)
- Anti-SSB antibody (La)
- Antithyroglobulin antibody
- Anti-LKM antibody
- Antiphospholipid antibody
- Anti-islet cell antibody
- Antineutrophil cytoplasm antibody
- C3
- C4
- CH50

- HCV quantitative viral load by quantitative RNA PCR-based assay (Cobas TaqMan HCV test (as outlined in Section 11.1.2).
- Placement of Quantitative DTH test (skin tests for tetanus and *C.* antigens) to be performed and read 48 to 72 hours later (AND just prior to infusion if not included at the 48 to 72 hour time point), as outlined in Appendix 2.
- Lymphocyte phenotype analysis by FACS to include the following phenotypic markers:
  - CD3
  - CD4
  - CD8
  - CD19
  - CD16
  - CD56
  - CD4+CD25
  - CD4+HLA-DR
  - CD8+HLA-DR
  - CD4+45RO
  - CD8+45RO
- Cryopreserved PBMCs.
- Serum for cytokines, and anti-tetanus antibody titers. Cytokine analytes may include: 2’5’ Oligoadenylate synthetase (2’5’ OAS), neopterin, IP-10, IL-1, 2, 4, 5, 6, 8, 10, 12, 13 and IFN gamma, TNF alpha and TGF beta -1.
- Current medication.
- Collection of baseline signs and symptoms.

11.1.4. Treatment Period

This period begins with the first i.v. infusion on Visit 3 (Day 1) and continues through the end of Visit 5 (Day 3). Subjects who meet selection criteria may start treatment with MDX-1106 or placebo within 6 to 28 days of screening tests. The subject will be given a single i.v. infusion of

MDX-1106 or placebo as outlined in Section 7.1. During this Treatment Period, the following data will be collected and recorded on the CRF at each scheduled visit (or as indicated):

- MDX-1106 or placebo infusion (Visit 3, Day 1).
- Vital sign measurements as outlined in Section 11.1.2 (excluding weight and height). On the day of infusion, vital sign measurements will be collected prior to the infusion and every 15 minutes for 1.5 hours post infusion start time, and at 2, 3, 4, and 6 hours post infusion start time (see Table 2). Subjects who experience an adverse event, including an infusion reaction, of Grade 2 during the 6 hour post dose observation period that does not resolve during this time, or of Grade ≥ 3 regardless of resolution, will be observed for a minimum of an additional 18 hours or until the adverse event resolves with vital sign measurements every 4 hours and additional evaluations as medically indicated for the management of the adverse event.
- Abbreviated physical examinations as outlined in Section 11.1.3 will be performed at Visit 3 at 4 hours post infusion start time, and at Visits 4 (Day 2) and 5 (Day 3) . Any new findings from baseline that are deemed significant should be assessed and if appropriate, be recorded as an adverse event in the eCRF.
- Electrocardiogram (Visit 4).
- Hematology and biochemistry laboratory tests (as outlined in Section 11.1.2) (to be drawn 4 hours post infusion start time at Visit 3 (Day 1), and at Visits 4 (Day 2) and 5 (Day 3).
- Urinalysis (as outlined in Section 11.1.2) to be collected 4 hours post infusion start time at the Visit 3 (Day 1) and at Visit 5 (Day 3).
- Pharmacokinetic sampling (as outlined in Table 2 and Section 11.2) (to be drawn prior to infusion and as further detailed in PK assessment schedule).
- Immunogenicity sampling (Visit 3 [Day 1]) (to be drawn prior to infusion).
- Drug and alcohol screen (Visit 3 [Day 1]). Urine toxicology screen to be performed prior to infusion; blood alcohol to be drawn prior to infusion.
- HCV viral load (pre-infusion and at 4 hours post infusion Visit 3 (Day 1) and at Visit 4 (Day 2) and Visit 5 (Day 3).
- Quantitative DTH test (Appendix 2) reading to be obtained at Visit 3 (Day 1), (prior to dosing), and at 24 hours (Visit 4 [Day 2]) and 48 hours (Visit 5 [Day 3]) post infusion end time.
- Lymphocyte FACS (Visit 3 [Day 1, to be drawn 4 hours post infusion start time] and at Visit 4 [Day 2] and Visit 5 [Day 3]) (as outlined in Section 11.1.3).

- Cryopreserved PBMCs (Visit 3 [Day 1, to be drawn 4 hours post infusion start time] and at Visit 4 [Day 2] and Visit 5 [Day 3]).
- Serum for cytokines and anti-tetanus antibody titers. (Visit 3 [Day 1 to be drawn 4 hours post infusion start time] and at Visit 4 [Day 2] and Visit 5 [Day 3]) (as outlined in Section 11.1.3).
- Concomitant medication.
- Adverse event assessment including specific elicitation of symptoms that may be indicative of immune-related adverse events (see Appendix 5). Drug and alcohol tests may be conducted at the time that an adverse event occurs if, in the clinical judgment of the Investigator, there is a possibility that such abuse may be a contributory factor to the adverse event.

11.1.5. Follow-Up Period

Subjects will return for follow-up evaluations on Days 8, 15, 22, 29, 31, 43, and 57. At follow-up visits, subjects will undergo the following:

- Vital sign measurements (excluding weight and height) (as outlined in Section 11.1.2).
- Abbreviated physical examination (as outlined in Section 11.1.3).
- Hematology (as outlined in Section 11.1.2).
- Biochemistry (as outlined in Section 11.1.2).
- Urinalysis (as outlined in Section 11.1.2; Visits 6 [Day 8] and 9 [Day 29]).
- Prothrombin time and INR (Visits 6 [Day 8] and 9 [Day 29]) (as outlined in Section 11.1.2).
- Immune safety assays (as outlined in Section 11.1.3; Visits 6 [Day 8] and 9 [Day 29]).
- Pharmacokinetic sampling (as outlined in Section 11.2).
- Immunogenicity sampling (Visit 9 [Day 29]).
- HCV viral load.
- DTH skin test (to be placed at Visit 9 [Day 29] and read 48 to 72 hours post placement [Day 31]).
- Lymphocyte FACS (Visits 6 [Day 8], 7 [Day 15], 9 [Day 29], and 11 [Day 57]) (as outlined in Section 11.1.3).
- Cryopreserved PBMCs (Visits 6 [Day 8], 7 [Day 15], 9 [Day 29], and 11 [Day 57]).
- Serum for cytokines and anti-tetanus antibody titers (Visits 6 [Day 8], 7 [Day 15], 9 [Day 29], and 11 [Day 57]).

- Concomitant medications.
- Adverse event assessment as described in Section 11.1.4, including specific elicitation of symptoms that may be indicative of immune related adverse events (see Appendix 5). Drug and alcohol tests may be conducted at the time that an adverse event occurs if, in the clinical judgment of the Investigator, there is a possibility that such abuse may be a contributory factor to the adverse event.

11.1.6. Off Study Visit

An Off Study visit will be scheduled at the time of completion or early withdrawal for all subjects treated in the study. If a subject prematurely withdraws prior to Day 85 (Visit 12), these procedures will be performed as soon as possible from the day of withdrawal from the study.

When subjects are withdrawn from the study as defined below, the reason for withdrawal will be documented on the CRF and in the source document. All subjects will be followed for adverse events and concomitant medications until Day 85, or if the subject withdraws prior to Day 85, for 70 days following administration of MDX-1106 and/or until resolution/stabilization of any adverse event suspected to be related to MDX-1106 treatment. For subjects who withdraw prior to 70 days, such follow-up will be sought by contacting the subject (preferably by telephone) every 28 days until the 70-day endpoint is reached.

- Serum β–HCG pregnancy test (as outlined in Section 11.1.3).
- Vital sign measurements (excluding weight and height) (as outlined in Section 11.1.2).
- Complete physical examination (as outlined in Section 11.1.2)
- Chest radiograph.
- Electrocardiogram.
- Clinical laboratory tests (as outlined in Section 11.1.2).
- Prothrombin time and INR.
- Immune safety assays (as outlined in Section 11.1.3).
- Pharmacokinetic sampling (as outlined in Section 11.2).
- Immunogenicity sampling.
- HCV viral load.
- Lymphocyte FACS (as outlined in Section 11.1.3).
- Cryopreserved PBMCs (as outlined in Section 11.1.3).
- Serum for cytokines, and anti-tetanus antibody titers (as outlined in Section 11.1.3).

- Concomitant medications.
- Adverse event assessment as described in Section 11.1.4 including specific elicitation of symptoms that may be indicative of immune related adverse events (see Appendix 5). Drug and alcohol tests may be conducted at the time that an adverse event occurs if, in the clinical judgment of the Investigator, there is a possibility that such abuse may be a contributory factor to the adverse event.

Off Study Category

The category of Off Study for each subject, as defined below, will be documented on the CRF page.

A. Early Withdrawal: Subjects who discontinue the study prior to Day 85. Subjects who discontinue prior to Day 29 for reasons other than a DLT or safety concerns will be replaced.

B. Completed: Subjects who received a single dose of MDX-1106 or placebo and completed all follow-up evaluations and Day 85 visit.

If for any reason, either study treatment or observations are discontinued, the reason will be recorded. Reasons for discontinuation include 1 of the following:

- Adverse event(s)
- Protocol violation
- Subject withdrew consent
- Subject is lost to follow-up
- Unsatisfactory therapeutic effect
- Disease progression
- Death
- Other

11.2. Pharmacokinetic Evaluations

Blood samples for the analysis of serum concentrations of MDX-1106 will be drawn in all subjects, regardless of treatment assignment, according to the schedule listed below and in Table 2 of the Time and Event Schedule. Samples should be drawn from a site other than the infusion site (i.e., contralateral arm). Samples from placebo subjects may not be analyzed.

Treatment Period

- **Visit 3 (Day 1):** pre-infusion, immediately post infusion and/or at 1-hour post infusion start time, and at 1.25, 1.5, 2, 3, 4, and 6 hours post-infusion start time. [If the infusion is slowed beyond the 60-minute timeframe, then the post-infusion samples will be collected relative to the infusion end time (at end of infusion, 0.25, 0.5, 1, 2, 3, and 5 hours after end of infusion)]. Additional samples will be taken at 0.25, 0.5, and 0.75 hours post infusion start time for subjects in the first cohort (0.03 mg/kg) and at 0.5, and 0.75 hours post infusion start time for subjects in the second cohort (0.1 mg/kg).
- **Visits 4 and 5 (Days 2 and 3):** 24 and 48 hours post infusion start time.

Follow-up Period

- **Days 8, 15, 22, 29, 43, and 57:** A single sample will be taken on each day.

Off Study Visit

- Day 85 or day of early withdrawal: A single sample will be taken.

12. EFFICACY EVALUATIONS

12.1. Primary Efficacy Parameter

The primary efficacy parameter is the proportion of MDX-1106-treated subjects in each dosing cohort and placebo-treated subjects who experience a 0.5-log or greater decline from baseline viral load, that is repeated on at least 2 consecutive measures.

12.2. Secondary Efficacy Parameter

The secondary efficacy parameters include the following: 1) changes in Hepatitis C viral load from baseline, by magnitude and duration, and in relation to the administered dose of MDX-1106 or placebo; 2) changes in levels of serum cytokines and PBMC reactivity to Hepatitis C viral antigens from baseline and in relation to MDX-1106 or placebo dose; 3) changes in DTH reactivity to a *Candida*/tetanus antigen skin test, anti-tetanus antibody titers, and PBMC cellular response to common recall antigens in relation to the MDX-1106 or placebo dose.

13. SAFETY EVALUATIONS

The following evaluations will be performed during the study to measure the safety and tolerability of MDX-1106: vital sign measurements, clinical laboratory tests, immunogenicity evaluations, physical examinations, ECGs, and the incidence and severity of adverse events. Drug and alcohol tests may also be performed if such abuse is considered a contributory factor to an adverse event.

13.1. Safety Precautions

The following safety precautions will be required in this study:

- Subjects will be observed for infusion reactions over a 6-hour observation period at the investigative unit (i.e., hospital or clinic unit with resuscitative capabilities) with ready access to an intensive care unit. No 2 subjects will be treated with MDX-1106 on the same day for the first 2 cohorts.
- Subjects who experience an adverse event, including an infusion reaction, of Grade 2 during the 6 hour post dose observation period that does not resolve during this time, or of Grade ≥ 3 infusion reaction, regardless of resolution, will be observed for a minimum of an additional 18 hours or until the adverse event resolves with vital sign measurements every 4 hours and additional evaluations as medically indicated for the management of the adverse event.
- Prior to discharge, subjects will be informed regarding a wide range of symptoms that may be associated with the development of late-occurring hypersensitivities and immune related adverse events, and will be given instruction regarding when they should contact the Investigator and/or report directly to the emergency room.
- All sites will have close access (within 60 minutes) to a hospital intensive care unit to which a subject can be transferred quickly.
- Medarex will be in regular telephone contact with the investigative sites during the dose-escalation phase of the study.
- Monitoring will include attention to both acute life-threatening cytokine release syndromes, as well as the emergence of more delayed immune-based toxicities.

14. ADVERSE EVENT REPORTING

Clinical events occurring after signing informed consent/HIPAA authorization, but prior to study drug administration are to be recorded on the Medical History/Current Medical Conditions CRF.

14.1. Definitions

An adverse event is any undesirable sign, symptom, clinically significant laboratory abnormality, or medical condition occurring after starting study treatment, even if the event is not considered to be treatment-related. Each adverse event is to be reported on an Adverse Event CRF page. Adverse events are graded using the DMID Adult Toxicity Tables November 2007 Draft (Appendix 3 and http://www3.niaid.nih.gov/research/resources/DMIDClinRsrch/toxtables.htm).

If DMID grading does not exist for an adverse event, the severity of mild (1), moderate (2), severe (3), life-threatening (4), and death related to an adverse event (5) will be used. Information about all adverse events, whether volunteered by the subject, discovered by Investigator questioning, or detected through physical examination, laboratory testing, or other means, will be collected and recorded on the Adverse Event CRF page and followed as appropriate. Adverse event monitoring should be continued for at least 70 days following the last dose of study drug until adverse event resolution/stabilization, or through all follow-up periods.

Medical conditions/diseases present before the infusion of study drug are only considered adverse events if they worsen after receiving any study drug. Clinical events occurring before the administration of study drug but after signing the informed consent form and providing HIPAA authorization are to be recorded on the Medical History/Current Medical Conditions CRF page. Abnormal laboratory values or test results constitute adverse events only if they induce clinical signs or symptoms or require therapy, in which case they should be recorded on the Adverse Events CRF page and should include the signs, symptoms, or diagnosis associated with them. As far as possible, each adverse event will also be described by:

1. Description
2. Duration (start and end dates)
3. DMID Grade 1 to 4 or severity if DMID not available
4. Relationship to the investigational product – attribution (by categories below)
5. Action(s) taken to treat the adverse event
6. Outcome

Relationship to Investigational Product

The relationship of each adverse event to study drug will be defined as “unrelated,” “unlikely,” “possible,” “probable,” or “definite.” The Investigator is responsible for determining the study drug relationship for each adverse event that occurs during the study. Assessments are to be recorded on the appropriate CRF page.

Unrelated

It is beyond all reasonable doubt that the reported adverse event was not caused by the study drug.

Unlikely

There is little or no possibility that the study drug caused the reported adverse event; and another factor(s) including concurrent illnesses, progression and expression of the disease state, concurrent medications, or a reaction to concurrent medications appear to explain the adverse event.

Possible

The reported adverse event follows a reasonable temporal sequence from administration of the study drug, but could reasonably be explained by the subject's clinical state or concurrent therapy.

Probable

The reported adverse event follows a reasonable temporal sequence from administration of the study drug, and could not reasonably be explained by the subject's clinical state or concurrent therapy.

Definite

The reported adverse event follows an anticipated response to the study drug; and is confirmed by both improvement upon stopping of study drug (withdrawal) and reappearance of the reaction on repeated exposure (rechallenge).

Action Taken

The actions taken in response to an adverse event are described on a numerical scale (from 0 to 5) that covers the various possibilities. One or more of these are to be selected:

0 No action taken
1 Investigational product dosage adjusted/temporarily interrupted
2 Investigational product permanently discontinued due to this adverse event
3 Concomitant medication taken
4 Non-drug therapy given
5 Hospitalization/prolonged hospitalization

14.2. Protocol-Defined Adverse Events of Importance

All events of colitis ≧ Grade 2 are deemed of special interest and should be reported using the serious adverse event reporting procedures, even if the event itself is not deemed serious. A diarrhea management algorithm is provided in the Investigator Brochure for treatment of this

adverse event, should it arise. Any Grade 2 event of colitis (per CTCAE) that also results in additional medical requirements, such as high dose steroids, blood transfusion or intravenous hyperalimentation, will be defined as a Grade 3 adverse event.

14.3. Serious Adverse Events

Information about all serious adverse events will be collected and recorded on the Serious Adverse Event Report Form. To ensure subject safety, each serious adverse event must also be reported to Medarex within 24 hours of learning of its occurrence.

All adverse events that potentially meet DLT criteria, as well as any unexpected toxicity must be reported to Medarex within 24 hours. Medarex will communicate confirmed DLTs and other toxicities to the study site as necessary to facilitate subject safety. A serious adverse event is defined in general as an untoward (unfavorable) event which:

1. is fatal or life-threatening;
2. requires or prolongs hospitalization;
3. is significantly or permanently disabling or incapacitating;
4. constitutes a congenital anomaly or a birth defect; or
5. may jeopardize the subject and may require medical or surgical intervention to prevent 1 of the outcomes listed above.

Hospitalizations occurring under the following circumstances are not considered serious adverse events: protocol-mandated hospitalization/observation for adverse event/infusion reaction that would otherwise not be routinely admitted (e.g., ongoing Grade 2, or resolved Grade 3 reaction); admission to a hospice for respite care; hospitalizations planned before entry into the clinical study; hospitalization for elective treatment of a condition unrelated to the studied indication or its treatment; hospitalization on an emergency, outpatient basis that does not result in admission (unless fulfilling the criteria above); hospitalization as part of the normal treatment or monitoring of the studied indication; hospitalization to facilitate the work up of a ≤ Grade 2 adverse event; and hospitalization not associated with any deterioration in condition.

14.4. Instructions for Rapid Notification of Serious Adverse Events

14.4.1. Reporting Responsibility

Any serious adverse event occurring in a subject after providing informed consent (and HIPAA authorization in the United States), while receiving study treatment, or in the 70 days following study drug administration or follow-up must be reported. The timeframe after discontinuing study drug may be extended if there is a strong suspicion that the study drug has not yet been

eliminated or the pharmacodynamic effects of the study drug persist beyond 70 days. All serious adverse events must also be reported for the timeframe in which the study drug interferes with the standard medical treatment given to a subject.

Each serious adverse event must be reported by the Investigator to Medarex, Inc. Pharmacovigilance (PVG) Desk, or designee within 24 hours of learning of its occurrence, even if it is not felt to be related to study drug. The report must include the concomitant medication(s) used to treat the event. Follow-up information about a previously reported serious adverse event must also be reported to Medarex or designee within 24 hours of receiving it. Medarex or designee may contact the Investigator to obtain further information on a reported Serious Adverse Event. If warranted, an Investigator Alert may be issued, to inform all Investigators involved in any study with the same study drug that a serious adverse event has been reported.

14.4.2. Reporting Procedures

The Investigator must complete the Serious Adverse Event Report Form in English, assess the causal relationship to study drug and send the completed form to the **SAE Reporting FAX Number** within 24 hours to Medarex or its designee. The monitor will review the Serious Adverse Event Form and supporting source documents during the monitoring visits.

Follow-up information should be sent to Medarex or its designee, within 24 hours of when the information is known. Either a new Serious Adverse Event Form is faxed (indicating that the information is a follow-up), or the original form may be re-faxed (with the new information highlighted and a new date provided). The follow-up report should describe whether the serious adverse event has resolved or is continuing, if and how it was treated, and whether the subject continued or permanently discontinued study participation. The form(s) and fax confirmation sheet(s) must be retained in the site’s study file.

14.4.3. Overdose

An overdose is defined as the accidental or intentional ingestion/infusion of any excessive dose of a product. For reporting purposes, Medarex, Inc. considers an overdose, regardless of adverse outcome, as a serious adverse event (see Section 14.3, Serious Adverse Events).

14.4.4. Pregnancy

All women of childbearing potential must have a negative pregnancy test prior to their infusion as specified in Section 11. If the pregnancy test is positive, the subject must not receive study drug (MDX-1106 or placebo) and must not continue in the study.

Pregnancy testing must also be performed throughout the study as specified in Tables 1 and 2 and Section 11 and the results of all pregnancy tests (positive or negative) will be recorded. In

addition, all women of childbearing potential should be instructed to contact the Investigator immediately if they suspect they might be pregnant (e.g., missed or late menstrual period) at any time during study participation.

14.4.4.1. *Reporting of Pregnancy*

If, following initiation of treatment with study drug, it is discovered that a subject is pregnant or may have been pregnant at the time of exposure to study drug, the study drug will be permanently discontinued. Additionally, females who received study drug must report any pregnancies that occur within 70 days of their dose of study drug. For males who received study drug, they must report any pregnancies in female partners that occur within 180 days of their dose of study drug.

The Investigator must immediately notify the Medarex medical monitor of this event and record the pregnancy on a Serious Adverse Event Form. Initial information on a pregnancy must be reported immediately to Medarex and the outcome information provided once the outcome is known. The Serious Adverse Event Form must be faxed to Medarex according to Serious Adverse Event reporting procedures described in Section 14.4.2.

Protocol-required procedures for study discontinuation and follow-up must be performed on the subject unless contraindicated by pregnancy (e.g., x-ray studies). Other appropriate pregnancy follow-up procedures should be considered if indicated. Follow-up information regarding the course of the pregnancy, including perinatal and neonatal outcome must be reported. Infants should be followed for a minimum of 8 weeks.

14.4.5. Contact Persons and Numbers

The Medarex Central Emergency Contact telephone and SAE telefax numbers are listed on the cover page of the protocol.

15. STATISTICAL METHODS

15.1. Sample Size Determination

The sample size of up to 54 subjects (up to 36 subjects in the dose-escalation phase, 6 subjects in the expansion cohort, and 12 subjects in the interferon naïve cohort) for this study is based on the study design for dose escalation, safety evaluation requirements, and preliminary estimate of efficacy. Cohorts of 6 subjects who are interferon experienced will be enrolled at MDX-1106 dose levels of 0.03, 0.1, 0.3, 1, 3, and 10 mg/kg. One of the 6 subjects will be randomly assigned to the placebo arm at each dose level. After the dose escalation portion of the study, an expansion cohort with 1 subject treated with placebo and 5 subjects treated with MDX-1106 will

be enrolled at or below the MTD or the highest tested dose if the MTD is not identified, to obtain additional safety information and preliminary efficacy estimation at that dose level. A cohort of 12 subjects naïve to interferon based HCV therapy will be enrolled in parallel with the interferon experienced expansion cohort, and randomly assigned to receive either placebo (2 subjects) or MDX-1106 (10 subjects) at the same dose level as the expansion cohort. This sample size determination is not based on a statistical power consideration.

15.2. Study Populations

15.2.1. Safety Population

The safety population includes subjects who receive at least 1 dose or any partial dose of MDX-1106 or placebo.

15.2.2. Per-protocol Population

The Per-Protocol population (PPP) includes all subjects in the safety population who received any dose or partial dose of study medication, have the correct disease diagnosis, no major violation of inclusion/exclusion criteria, and complete the Day 29 assessment or who prematurely discontinue prior to Day 29 due to safety concerns.

15.2.3. Pharmacokinetic Population

The pharmacokinetic population includes all subjects in the safety population who complete the infusion with MDX-1106 and complete at least up to Day 29 of pharmacokinetic assessments.

15.3. Statistical Consideration

The Biostatistics group at Medarex, Inc. or its designees will analyze the data collected in this study.

Baseline is defined as the last valid measurement before the dose of MDX-1106 or placebo.

All data will be listed individually by subject. Continuous variables will be summarized using the following descriptive statistics: mean, standard deviation, median, quartiles (Q1, Q3), and minimum and maximum values. Categorical variables will be reported as frequencies and percentages.

Unless otherwise indicated, statistical significance will be declared if the two-sided P-value is ≤ 0.05.

15.3.1. Demographics and Baseline Characteristics

Subject demographics and baseline characteristics including age, sex, race, height, weight, disease information, and medical conditions will be summarized by dose level and by treatment group (MDX-1106 or placebo) using descriptive statistics.

15.3.2. Extent of Exposure

The total MDX-1106 dosage (mg) will be summarized in a table by dose cohort and treatment group using descriptive statistics. The listing of treatment exposure will be generated.

15.3.3. Concomitant Medication

Concomitant medications will be coded using the World Health Organization (WHO) Drug Dictionary. Concomitant medications will be summarized. Tabulation will be made with respect to the proportion of subjects taking at least 1 concomitant medication for each preferred term during the study. A listing of concomitant medications by subject will be provided.

15.3.4. Efficacy

The efficacy analysis will be conducted on the safety population and per-protocol population. Per-protocol population is the primary population for preliminary efficacy analysis.

The primary efficacy endpoint is the occurrence of a 0.5-log or greater decline from baseline viral load, which is repeated on at least 2 consecutive measures, and will be summarized using descriptive statistics by dose cohort of MDX-1106 and by treatment group (MDX-1106 or placebo). The exact 95% confidence interval will be estimated for the expansion dose cohort.

The secondary efficacy endpoint, change in Hepatitis C viral load from baseline, will be summarized by dose cohort of MDX-1106 and by treatment group (MDX-1106 or placebo) using descriptive statistics.

Changes in levels of serum cytokines and PBMC reactivity to Hepatitis C viral antigens from baseline will be summarized by dose cohort of MDX-1106 and by treatment group (MDX-1106 or placebo) using descriptive statistics.

Changes in delayed-type hypersensitivity (DTH) reactivity to a *Candida*/tetanus antigen skin test, anti-tetanus antibody titers, and PBMC responses to common recall antigens in relation to the MDX-1106 dose will be summarized by dose cohort of MDX-1106 and by treatment group (MDX-1106 or placebo) using descriptive statistics.

15.3.5. Safety

The safety analysis will be conducted on the safety population. The following safety parameters will be evaluated:

Adverse Events

Adverse event is defined as any undesirable sign or symptom that emerges during treatment having been absent pre-treatment or that has worsened relative to the pre-treatment state. All adverse events will be summarized using descriptive statistics by system organ class, preferred term, severity, seriousness, and relationship to the investigational product. The severity is graded using the DMID Adult Toxicity Tables November 2007 Draft (Appendix 3). Infusion reactions should be graded according to NCI Common Terminology Criteria for Adverse Events (Version 3.0) guidelines, http://ctep.cancer.gov (no specific guidelines regarding infusion reactions are included in the DMID criteria).

Adverse events will be coded using MedDRA to get a preferred term and a system/organ class term for each event. The number of subjects who experienced at least 1 adverse event, treatment-related adverse event, serious adverse event, treatment-related serious adverse event, and the number of subjects withdrawn due to adverse events will be summarized. For each system/organ class and preferred term, summaries will be made with respect to the number and proportion of subjects having at least 1 occurrence of that adverse event during the study. The incidence of adverse events will be presented overall, by system/organ class and preferred term, and additional grouping by severity and relationship to study drug. Individual listing of adverse events will be provided.

Vital Signs

Vital signs and changes in vitals signs from baseline to each post-baseline visit will be summarized by dose level and treatment group (MDX-1106 or placebo) using descriptive statistics. Vital sign abnormalities will be listed or summarized by dose level and treatment group using descriptive statistics.

Clinical Laboratory Tests

Clinical laboratory test values outside the normal range will be flagged in the data listing.

Laboratory data will be summarized by dose level and by treatment group (MDX-1106 or placebo) using shift tables (baseline to post-baseline value). The absolute value of lab test and change from baseline will be summarized by dose level and by treatment group (MDX-1106 or placebo) using descriptive statistics.

DMID grade will be assigned to the laboratory parameters, which are included in DMID Adult Toxicity draft November 2007. Laboratory values will be listed. The lab values which are outside normal range will be flagged as H (above high normal limit) or L (below lower normal limit) in the listings. The DMID grade will also be flagged in the listings.

Other Safety Variables

Other safety variables will be summarized using descriptive statistics by dose level and by treatment group (MDX-1106 or placebo) if applicable.

15.3.6. Pharmacokinetic and Immunogenicity Parameters

Pharmacokinetic and immunogenicity parameters will be summarized by dose level using descriptive statistics.

Serum concentration of MDX-1106 will be determined by a validated method according to assessment schedules. The concentrations will be summarized by visit and schedule sample time using descriptive statistic for safety population by dose levels.

Definition and Calculation of PK Parameters

The following pharmacokinetic parameters will be estimated and reported:

$AUC_{0\text{-}t}$ Area under the concentration-time curve from the time of dosing to the time of the last observation (calculated by linear trapezoidal summation).

$AUC_{0\text{-}\infty}$ Area under the curve from the time of dosing extrapolated to infinity (calculated by the linear trapezoidal summation and extrapolated to infinity using C_{last}/λ_z).

C_{max} Maximum plasma concentration observed post-dose.

t_{max} Time at which the C_{max} occurs.

$t_{1/2}$ Elimination half-life, determined as $0.693/\lambda_{z.}$

The pharmacokinetic parameters, AUC, C_{max}, t_{max}, and $t_{1/2}$ will be summarized using descriptive statistics for pharmacokinetic population. Individual as well as mean concentration-time plots will be depicted.

15.4. Statistical Software

The estimation of pharmacokinetic parameters will be performed using WinNonlin® Version 3.3 or higher. All statistical analyses will be performed using SAS® Version 9.13 or higher.

16. ETHICAL ASPECTS

16.1. Ethics and Good Clinical Practice

This study will be conducted in accordance with Good Clinical Practice (GCP), as defined by the International Conference on Harmonisation (ICH) and in accordance with the ethical principles underlying European Union Directive 2001/20/EC and the United States Code of Federal Regulations, Title 21, Part 50 (21CFR50). The study will be conducted in compliance with the protocol. The protocol and any amendments and the subject informed consent will receive Institutional Review Board/Independent Ethics Committee (IRB/IEC) approval/favorable opinion prior to initiation of the study.

Study personnel involved in conducting this study will be qualified by education, training, and experience to perform their respective tasks. This study will not use the services of study personnel where sanctions have been invoked or where there has been scientific misconduct or fraud (e.g., loss of medical license, debarment).

16.2. Institutional Review Board/Independent Ethics Committee

Before implementing this study, the protocol, the proposed informed consent/HIPAA authorization, and other information to subjects must be reviewed by an IRB/IEC. A signed and dated statement that the protocol and Informed consent/HIPAA authorization have been approved by the IRB/IEC must be given to Medarex before study initiation. The name and occupation of the chairperson and the members of the IRB/IEC (preferred) or the IRB's HHS Assurance number must be supplied to Medarex. Any amendments to the protocol which need formal approval as required by local law or procedure will be approved by this committee. The IRB/IEC may be notified for all other administrative amendments (i.e., administrative changes).

16.3. Informed Consent

The Investigator or designee will explain to each subject (or legally authorized representative) the nature of the research study, its purpose, the procedures involved, the expected duration of study participation, alternative treatment, and the potential risks and benefits involved and any discomfort which may occur during the subject's participation in the study. Each subject will be informed that participation in the study is voluntary and that he/she may withdraw from the study at any time and that withdrawal of consent will not affect his/her subsequent medical treatment or relationship with the treating physician.

This informed consent should be given by means of a standard written statement, written in non-technical language. The subject should read and consider the statement before signing and

dating it, and should be given a copy of the signed document. No subject can enter the study before his/her informed consent has been obtained.

The informed consent form is part of the protocol, and must be submitted by the Investigator with the protocol for IRB/IEC approval. Medarex supplies a proposed informed consent form, which complies to regulatory requirements and is considered appropriate for the study. Any changes to the proposed consent form suggested by the Investigator must be agreed to by Medarex before submission to the IRB/IEC and a copy of the approved version must be provided to the Medarex monitor after IRB/IEC approval.

17. ADMINISTRATIVE REQUIREMENTS

17.1. Protocol Amendments

Any change or addition to this protocol requires a written protocol amendment that must be approved by Medarex, before implementation. Amendments significantly affecting the safety of subjects, the scope of the investigation or the scientific quality of the study, require additional approval by the IRB/IEC of all centers, and, in some countries, by the regulatory authority. A copy of the written approval of the IRB/IEC must be given to the Medarex monitor or their designee. Examples of amendments requiring such approval are:

1. Increase in drug dosage or duration of exposure of subjects;
2. Significant change in the study design (e.g., addition or deletion of a control group);
3. Increase in the number of invasive procedures to which subjects are exposed; or
4. Addition or deletion of a test procedure for safety monitoring.

These requirements for approval should in no way prevent any immediate action from being taken by the Investigator or by Medarex in the interests of preserving the safety of all subjects included in the study. If an immediate change to the protocol is felt to be necessary by the Investigator and is implemented by him/her for safety reasons Medarex should be notified and the IRB/IEC at the center should be informed within 10 working days.

Amendments affecting only administrative aspects of the study do not require formal protocol amendments or IRB/IEC approval but the IRB/IEC of each center must be kept informed of such administrative changes. Examples of administrative changes not requiring formal protocol amendments and IRB/IEC approval that can be treated as administrative amendments include but are not limited to:

1. Changes in the staff used to monitor studies (e.g., Medarex staff versus a contract research organization [CRO]); and

2. Minor changes in the packaging or labeling of study drug.

17.2. Monitoring Procedures

Before study initiation, at a site initiation visit or at an Investigator's meeting, a Medarex representative will review the protocol and CRF with the Investigators and their staff. During the study, the Medarex monitor or its designee will visit the site regularly to check the completeness of subject records, the accuracy of entries on the CRFs, the adherence to the protocol and to Good Clinical Practice, the progress of enrollment, and also to ensure that study drug is being stored, dispensed, and accounted for according to specifications.

The Investigator must give the monitor access to relevant hospital or clinical records to confirm their consistency with the CRF entries. No information in these records about the identity of the subjects will leave the study center. Medarex monitoring standards require full verification for the presence of informed consent, HIPAA authorization, adherence to the inclusion/exclusion criteria, documentation of serious adverse events and the recording of efficacy and safety variables. Additional checks of the consistency of the source data with the CRFs are performed according to the study-specific monitoring plan.

17.3. Recording of Data and Retention of Documents

Data on subjects recorded on CRFs (electronic or paper) during the study will be documented in an anonymous fashion and the subject will only be identified by the subject number, and by his/her initials if also required. If, as an exception, it is necessary for safety or regulatory reasons to identify the subject, both Medarex and the Investigator are bound to keep this information confidential.

All the information required by the protocol should be provided; any omissions require explanation. All CRFs (electronic or paper) should be completed and available for review within a timely manner, preferably no more than 10 days after the subject's visit (except for the last visit of the last subject, which should be completed in a timely manner, preferably within 5 working days), so that the monitor may check the entries for completeness and accuracy and legibility, ensure the CRF is signed by the Investigator and transmit the data to Medarex or designee.

In the event that paper CRFs are used, all entries to the CRFs must be made clearly in black ballpoint pen to ensure the legibility of self-copying or photocopied pages. Corrections will be made by placing a single horizontal line through the incorrect entry, so that the original entry can still be seen, and placing the revised entry beside it. The revised entry must be initialed and dated by a member of the Investigator's research team authorized to make CRF entries. Correction fluid must not be used.

The Investigator must maintain source documents for each subject in the study. All information on CRFs will be traceable to these source documents, which are generally maintained in the subject's file. The source documents will contain all demographic and medical information, including laboratory data, ECGs, etc., also a copy of the signed informed consent/HIPAA authorization, which should indicate the study number and title of the study.

Essential documents, as listed below, will be retained by the Investigator for as long as needed to comply with national and international regulations. Medarex will notify the Investigator(s)/institution(s) when the study-related records are no longer required. The Investigator agrees to adhere to the document retention procedures by signing the protocol. Essential documents include:

1. Signed protocol and all amendments;
2. IRB/IEC approvals for the study protocol and all amendments;
3. All source documents and laboratory records;
4. CRF copies;
5. Subjects' Informed consent/HIPAA authorization; and
6. Any other pertinent study document

17.4. Auditing Procedures

In addition to the routine monitoring procedures, Medarex or its designees may conduct audits of clinical research activities in accordance with internal SOPs to evaluate compliance with the principles of Good Clinical Practices. Medarex, its designee, or a regulatory authority may wish to conduct an inspection (during the study or after its completion). If an inspection is requested by a regulatory authority, the Investigator will inform Medarex, immediately that this request has been made.

17.5. Publication of Results

Any formal presentation or publication of data collected from this study will be considered as a joint publication by the Investigator(s) and the appropriate personnel of Medarex. Authorship will be determined by mutual agreement. For multicenter studies, it is mandatory that the first publication be based on data from all centers, analyzed as stipulated in the protocol by Medarex, statisticians, and not by the Investigators themselves. Investigators participating in multicenter studies agree not to present data gathered from 1 center or a small group of centers before the full, initial publication, unless formally agreed to by all other Investigators and Medarex.

Medarex must receive copies of any intended communication in advance of publication (at least 15 working days for an abstract or oral presentation and 30 working days for a journal submission). Medarex will review the communications for accuracy (thus avoiding potential discrepancies with submissions to health authorities), verify that confidential information is not being inadvertently disclosed and to provide any relevant supplementary information. Authorship of communications arising from pooled data may include members from each of the contributing centers as well as Medarex personnel.

17.6. Disclosure and Confidentiality

By signing the protocol, the Investigator agrees to keep all information generated in connection with the study or provided by Medarex, in strict confidence and to request similar confidentiality from his/her staff and the IRB/IEC. Study documents provided by Medarex (protocols, investigators' brochures, CRFs and other material) will be stored appropriately to ensure their confidentiality. Such confidential information may not be disclosed to others without direct written authorization from Medarex, except to the extent necessary to obtain informed consent/HIPAA authorization from subjects who wish to participate in the study.

17.7. Discontinuation of Study

Medarex reserves the right to discontinue any study for any reason at any time.

17.8. Data Management

17.8.1. Data Collection

Investigators must enter the information required by the protocol onto the Medarex CRFs that are printed on no carbon required paper or eCRF (electronic CRF) if electronic data capture (EDC) is deployed. Medarex monitors or designees will review the CRFs for completeness and accuracy, and instruct site personnel to make any required corrections or additions. The paper CRFs will be forwarded to Medarex or its designee, with 1 copy retained at the investigational site. Receipt of CRFs will be recorded by Medarex or its designee; the original copy will be placed in the Central Trial Master Files and the NCR copy will be forwarded to the responsible Medarex data management staff or its designee for processing. If EDC is used, electronic version of the final CRF book will be forwarded to the study sites for archival at the study closure.

17.8.2. Database Management and Quality Control

Data items from the paper CRFs will be entered into the study database using double data entry with verifications. If EDC is deployed, Medarex Study Monitor or designee will review the data entry as compared to the study site source document.

Subsequently, the information entered into the database will be systematically checked by Data Management staff following Medarex or its designee data management procedures. Obvious errors will be corrected by Medarex or its designee personnel. Other errors, omissions, or requests for clarification will be queried; queries will be returned to the investigational site for resolution. A copy of the signed Data Clarification Form will be kept with the CRFs, and once the original is received, the resolutions will be entered into the database. Quality control audits of all key safety and efficacy data in the database will be made after entering data from each visit. If EDC is deployed for the study, electronic queries will be used to communicate discrepant data with the study sites.

When the database has been declared to be complete and accurate, the database will be closed. Any changes to the database after that time can only be made by joint written agreement of the Medarex study team.

18. REFERENCES

1 Guidotti LG, Chisari FV. Immunobiology and pathogenesis of viral Hepatitis. Annu Rev Pathol: Mechanisms of Dis 2006;1(1):23-61.

2 Sharpe AH, Wherry EJ, Ahmed R, Freeman GJ. The function of programmed cell death 1 and its ligands in regulating autoimmunity and infection. Nat Immunol 2007;8(3)239-45.

3 Barber DL, Wherry EJ, Masopust D, Zhu B, Allison JP, Sharpe AH, et al. Restoring function in exhausted CD8 T Cells during chronic viral infection. Nat 2006;Feb 9(439):682-7.

4 Bernard A, Lamy L, Alberti I. The two-signal model of T-cell activation after 30 years. Transplant 2002;73:S31-35.

5 Greenwald RJ, Freeman GJ, Sharpe AH. The B7 family revisited. Annu Rev Immunol 2005;23:515-48.

6 Nishimura H, Honjo T. PD-1: an inhibitory immunoreceptor involved in peripheral tolerance. Trends Immunol 2001;22(5):265-8.

7 Freeman GJ, Long AJ, Iwai Y, Bourque K, Chernova T, Nishimura H, et al. Engagement of the PD-1 immunoinhibitory receptor by a novel B7 family member leads to negative regulation of lymphocyte activation. J Exp Med 2000;192(7):1027-34.

8 Latchman Y, Wood CR, Chernova T, Chaudhary D, Borde M, Chernova I, et al., PD-L2 is a second ligand for PD-1 and inhibits T cell activation. Nat Immunol 2001;2(3):261-8.

9 Carter LL, Fouser LA, Jussif J, Fitz L, Deng B, Wood CR, et al. PD-1:PD-L inhibitory pathway affects both CD4+ and CD8+ T cells and is overcome by IL-2. Eur J Immunol 2002;32(3):634-43.

10 Chemnitz JM, Parry RV, Nichols KE, June CH, Riley JL. SHP-1 and SHP-2 associate with immunoreceptor tyrosine-based switch motif of programmed death 1 upon primary human T cell stimulation, but only receptor ligation prevents T cell activation. J Immunol 2004;173(2):945-54.

11 Sheppard KA, Fitz LJ, Lee JM, Benander C, George JA, Wooters J. PD-1 inhibits T-cell receptor induced phosphorylation of the ZAP70/CD3zeta signalosome and downstream signaling to PKCtheta. FEBS Letters 2004 Sep 10;574(1-3):37-41.

12 Nishimura H, Nose M, Hiai H, Minato N, Honjo T. Development of lupus-like autoimmune diseases by disruption of the PD-1 gene encoding an ITIM motif-carrying immunoreceptor. Immun 1999;11(2):141-51.

13 Nishimura H, Okazaki T, Tanaka Y, Nakatani K, Hara M, Matsumori A, et al. Autoimmune dilated cardiomyopathy in PD-1 receptor-deficient mice. Science 2001;291(5502):319-22.

14 Wang J, Yoshida T, Nakaki F, Hiai H, Okazaki T, Honjo T. Establishment of NOD-Pdcd1-/- mice as an efficient animal model of type I diabetes. Proc Natl Acad Sci USA 2005;102(33):11823-8.

15 Okazaki T, Tanaka Y, Nishio R, Mitsuiye T, Mizoguchi A, Wang, J, et al. Autoantibodies against cardiac troponin I are responsible for dilated cardiomyopathy in PD-1-deficient mice. Nat Med 2003;9(12):1477-83.

16 Okazaki T, Honjo T. The PD-1-PD-L pathway in immunological tolerance. Trends Immunol 2006; 27(4):195-201.

(continued)

REFERENCES *(continued)*

17 Okazaki T, Otaka Y, Wang J, Hiai H, Takai T, Ravetch HV, et al. Hydronephrosis associated with antiurotherlial and antinuclear autoantibodies in BALB/c-Fcgr2b-/-Pdcd1-/-mice. J Exp Med 2005;202(12):1643-8.

18 Blazar BR, Carreno BM, Panoskaltsis-Mortari A, Carter L, Iwai Y, Yagita H, et al. Blockade of programmed death-1 engagement accelerates graft-versus-host disease lethality by an IFN-γ-dependent mechanism. J Immunol 2003;171(3):1272-7.

19 Keir ME, Liang SC, Guleria I, Latchman YE, Qipo A, Albacker LA, et al. Tissue expression PD-L1 mediates peripheral T cell tolerance. J Exp Med 2006;203(4):883-95.

20 Carter LL, Leach MW, Azoitei ML, Cui J, Pelker JW, Jussif J et al. PD-1/PD-L1, but not PD-1/PD-L2, interactions regulate the severity of experimental autoimmune encephalomyelitis. J Neuroimmunol 2007;182:124-34.

21 Salama AD, Chitnis T, Imitola J, Ansari MJ, Akiba H, Tushima F, et al. Critical role of the programmed death-1 (PD-1) pathway in regulation of experimental autoimmune encephalomyelitis. J Exp Med 2003;198(1):71-8.

22 Ansari MJ, Salama AD, Chitnis T, Smith RN, Yagita H, Akiba H, et al. The programmed death 1 (PD-1) pathway regulates autoimmune diabetes in nonobese diabetic (NOD) mice. J Exp Med 2003;198(1)63-9.

23 Okazaki T, Honjo T. Rejuvenating exhausted T cells during chronic viral infection. Cell 2006;124(3):459-61.

24 Yao S, Chen L. Reviving exhausted T lymphocytes during chronic virus infection by B7-H1 blockade. Trends Mol Med 2006 12(6):244-6.

25 Iwai Y, Terawaki S, Ikegawa M, Okazaki T, Honjo T. PD-1 inhibits antiviral immunity at the effector phase in the liver. J Exp Med 2003;198(1):39-50.

26 Urbani S, Amadei B, Tola D, Massari M, Schivazappa S, Missale G, et al. PD-1 expression in acute Hepatitis C virus (HSV) infection is associated with HCV-specific CD8 exhaustion. J Virol 2006;80(22):11398-403.

27 Radziewicz H, Ibegbu CC, Fernandez ML, Workowski KA, Obideen K, Wehbi M et al. Liver infiltrating lymphocytes in chronic human HCV infection display an exhausted phenotype with high PD-1 and low CD127 expression. J Virol 2006 [Epub] – replace with final publication.

28 Golden-Mason L, Palmer B, Klarquist J, Mengshol JA, Castelblanco N, Rosen HR. Upregulation of PD-1 Expression on Circulating and Intrahepatic HCV-specific CD8+ T Cells Associated with Reversible Immune Dysfunction. J Virol 2007 [Epub].

29 Trautmann L, Janbazian L, Chomont N, Said EA, Gimmig S, Bessette B, et al. Upregulation of PD-1 expression on HIV-specific CD8+ T cells leads to reversible immune dysfunction. Nat Med 2006;12(10):1198-202.

30 Day CL, Kaufmann DE, Kiepiela P, Brown JA, Moodley ES, Reddy S, et al. PD-1 expression on HIV-specific T cells is associated with T-cell exhaustion and disease progression. Nature 2006;443(7109):350-4.

(continued)

REFERENCES *(continued)*

31 Petrovas C, Casazza JP, Brenchley JM, Price DA, Gostick E, Adams WC, et al. PD-1 is a regulator of virus-specific CD8+ T cell survival in HIV infection. J Exp Med 2006;203(10):2281-92.

32 Petrovas C, Price DA, Mattapallil J, Ambrozak DR, Geldmacher C, Cecchinato V, et al. SIV-specific CD8+ T cells express high levels of PD1 and cytokines but have impaired proliferative capacity in acute and chronic SIVmac251 infection.

33 Freeman GJ, Wherry EJ, Ahmed R, Sharpe AH. Reinvigorating exhausted HIV-specific T cells via PD-1-PD-1 ligand blockade. J Exp Med 2006;203(10):2223-7.

34 Riley JL, June CH. The road to recovery: translating PD-1 biology into clinical benefit. Trends in Immunol; article in press:www.sciencedirect.com.

35 Pardoll D. Does the immune system see tumors as foreign or self? Annu Rev Immunol 2003;21:807-39.

36 Iwai Y, Terawaki S, Honjo T. PD-1 blockade inhibits hematogenous spread of poorly immunogenic tumor cells by enhanced recruitment of effector T cells. Int Immunol 2004;17(2):133-44.

37 Iwai Y, Ishida M, Tanaka Y, Okazaki T, Honjo H, Minato N. Involvement of PD-L1 on tumor cells in the escape from host immune system and tumor immunotherapy by PD-L1 blockade, Proc Natl Acad Sci USA 2002;99(19):12293-7.

38 Strome SE, Dong H, Tamura H, Voss SG, Flies DB, Tamada K, et al. B7-H1 blockade augments adoptive T-cell immunotherapy for squamous cell carcinoma. Cancer Res 2003;63:6501-5.

39 Dong H, Strome SE, Salomao DR, Tamura H, Hirano F, Flies DB, et al. Tumor-associated B7-H1 promotes T-cell apoptosis: a potential mechanism of immune evasion. Nat Med 2002;8(8):793-800.

40 Hirano F, Kaneko K, Tamura H, Dong H, Wang S, Ichikawa M, et al. Blockade of B7-H1 and PD-1 by monoclonal antibodies potentiates cancer therapeutic immunity. Cancer Res 2005;65(3):1089-96.

41 Blank C, Brown I, Peterson AC, Spiotto M, Iwai Y, Honjo T, et al. PD-L1/B7H-1 inhibits the effector phase of tumor rejection by T cell receptor (TCR) transgenic CD8+ T cells. Cancer Res 2004;64:1140-45.

42 Konishi J, Yamazaki K, Azuma M, Kinoshita I, Dosaka-Akita H, Nishimura M. B7-H1 Expression on non-small cell lung cancer cells and its relationship with tumor-infiltrating lymphocytes and their PD-1 expression. Clin Cancer Res 2004;10(15):5094-100.

43 Thompson RH, Gillett MD, Cheville JC, Lobse CM, Dong H, Webster WS, et al. Costimulatory B7-H1 in renal cell carcinoma patients: indicator of tumor aggressiveness and potential therapeutic target. Proc Natl Acad Sci USA 2004;101(49):17174-9.

44 Ohigashi Y, Sho M, Yamada Y, Tsurui Y, Hamada K, Ikeda N, et al. Clinical significance of programmed death-1 ligand-1 and programmed death-1 ligand-2 expression in human esophageal cancer. Clin Cancer Res 2005;11(8):2947-53.

45 Tsushima F, Tanaka K, Otsuki N, Youngnak P, Iwai H, Omura K, et al. Predominant expression of B7-H1 and its immunoregulatory roles in oral squamous cell carcinoma. Oral Oncol 2005 (article in press).

(continued)

46 Thompson RH, Webster WS, Cheville JC, Lohse CM, Dong H, Leibovich BC, et al. B7-H1 glycoprotein blockade: a novel strategy to enhance immunotherapy in patients with renal cell carcinoma. Urology 2005;66(5A):10-14.

47 Thompson RH, Gillett MD, Cheville JC, Lohse CM, Dong H, Webster WS, Chen L, et al. Costimulatory Molecule B7-H1 in primary and metastatic clear cell renal cell carcinoma. Cancer 2005;104(10):2084-91.

48 Dong H, Chen L. B7-H1 pathway and its role in the evasion of tumor immunity. J Mol Med 2003;81(5):281-7.

49 Fong T, Valinluck B, Govindarajan S, Charboneua F, Adkins RH, Redeker AG. Short-Term Prednisone Therapy Affects Aminotransferase Activity and Hepatitis C Virus RNA Levels in Chronic Hepatitis C. Gastroenterology 1994; 107: 196-199.

50 Chayama K, Tsubota A, Kobayashi M, Hashimoto M, Miyano Y, Koike H, Kobayahsi M, et al. A Pilot Study of Corticosteroid Priming for Lymphoblastoid Interferon Alfa in Patients with Chronic Hepatitis C. Hepatology 1996; 23: 953-957.

51 Magrin S, Craxi A, Fabiano C, Fiorentino G, Almasio P, Palazzo U, Pinzello G, et al. Hepatitis C Virus Replication in Autoimmune Chronic Hepatitis. Hepatology 1991. 13: 364-367.

52 Thiele D, DuCharme L, Cunningham M, Mimms L, Cuthbert J, Lee W, Combes B. Steroid Therapy of Chronic Hepatitis: Characteristics Associated with Response in Anti-Hepatitis C Virus-Positive and-Negative Patients. Gastroenterology 1996; 91:300-308.

53 Schiano T, Te H, Thomas R, Hussain H, Bond K, Black M. Results of Steroid-Based Therapy for the Hepatitis C-Autoimmune Hepatitis Overlap Syndrome. Gastroenterology 2001; 96: 2984 – 2991.

54 Ramos-Casals M, Trejo O, Garcia-Carrasco M, Font J. Therapeutic Management of Extrahepatic Manifestations in Patients with Chronic Hepatitis C Virus Infection. Rheumatology 2003; 42:818-828.

19. APPENDICES

Appendix 1: Categories of Prior HCV Treatment Experience

Subjects must have received a prior course of interferon/ribavirin based treatment.

1. **Treatment Naïve** – No prior treatment for HCV
2. **Treatment Experienced** – IFN based therapy:

   2a. Prior therapy with less than 12 weeks of IFN or PEG-IFN based therapy (inadequate therapy)

   Prior therapy with approved doses of IFN or PEG-IFN and RBV, but with currently active disease, categorized by:

   2b. Receipt of adequate previous IFN or PEG-IFN and RVN therapy for a minimum of 12 weeks (PEG-IFN alpha-2a doses of > 180 μg/week or PEG-IFN alpha-2b 1.5 μg/kg/week and at least 600 mg RBV daily) not resulting in a minimum of a 2-log decrease in serum HCV RNA concentrations while on treatment (null responders); or

   2c. Receipt of adequate previous IFN or PEG-IFN and RBV therapy for a minimum of 24 weeks (PEG-IFN alpha-2a doses of ≥ 180 μg/week or PEG-IFN alpha-2b 1.5 μg/kg/week and at least 600 mg RBV daily) resulting in a minimum of a 2-log decrease in serum HCV RNA concentrations by 12 weeks of treatment but not resulting in an undetectable viral load after 24 weeks of treatment (partial responders); or

   2d. Receipt of adequate previous IFN or PEG-IFN and RBV therapy for a minimum of 24 weeks (PEG-IFN alpha-2a doses of ≥ 180 μg/week or PEG-IFN alpha-2b 1.5 μg/kg/week and at least 600 mg RBV daily) resulting in a minimum of a 2-log decrease in serum HCV RNA concentrations by 12 weeks of treatment and an undetectable viral load after 24 weeks of treatment, but subsequent relapse in viremia (relapsers).

   2e. Prior treatment did not meet criteria (incomplete therapy) e.g., a subject treated for more than 12 weeks with an initial 2 log reduction in viremia, but who stopped due to adverse events and never reached a timepoint of 24 weeks on therapy to assess for an SVR.
3. **Treatment Experienced** – Other

   Includes other unapproved or investigational agents.

Details of prior HCV treatment history that corroborate classification above should be included in source documents (but will not be collected in CRFs).

Appendix 2: Instructions for Placing and Reading the DTH Skin Tests for Tetanus and *Candida* Antigens

PROCEDURE FOR PLACING THE DELAYED TYPE HYPERSENSITIVITY (DTH) TEST

Before beginning check with the subject, to determine whether the subject has ever had a positive reaction to *Candida* or tetanus antigen testing, and also identify any allergies that the subject may have that may contraindicate using a specific antigen. An antigen (*Candida* or tetanus) test should NOT be administered to a subject with a known prior history of a severe reaction (e.g., vesiculation, ulceration or necrosis) to antigen exposure that can occur in highly sensitive persons. (Subjects with history of severe reaction to one antigen may still be administered the test to the other antigen if no contraindication).

Supplies

- 1 ml tuberculin syringes with 26-27 gauge needles (1/2" - 5/8" length)
- Gloves
- Alcohol swabs
- Marking pen
- induration ruler or millimeter ruler
- Test antigens: Candida and Tetanus

Procedure

1) Assemble all necessary equipment and wash hands. Don gloves.
2) The forearm is the preferred site of injection. Select injection site and cleanse skin. Space injection sites at center of forearm, with *Candida* test site on left arm first, and Tetanus test site on right arm second (for consistency). If forearm cannot be used, use backside of upper arm or thigh.
3) Draw up 0.1 mL of Candin and 0.1 mL of Tetanus into separate syringes.
4) Hold the skin taut with thumb and forefinger. Select a site that is at least 2" (5 cm) from an intravenous site and is free from lesions, hair and tattoos. The syringe should be positioned with the bevel up at a 10-15° angle above the subject's arm.
5) Insert the needle bevel up, just below the epidermis (approximately 2 mm).When the bevel of the needle is fully inserted, an outline of the needle should be visible under the skin. If

not visible, the needle has been inserted too deeply and should be partially retracted without exposing the bevel.

6) When the needle is in place, inject the antigen slowly (resistance will be felt). The solution will cause the skin to blanch and form a sharply defined wheal or bleb 6-10 mm in diameter. A wheal will not form if the antigen is injected too deeply. If the wheal is less than 6 mm in diameter, repeat the process with a new needle approximately 1" below the first site.

7) Withdraw the needle quickly. The bleb will disappear within minutes. Do not massage or use dressing. Blot if necessary. Circle and name the area with a pen (Use C for C*andida*, T for tetanus). Record it in the medical record.

8) Repeat the process with the second antigen test.

9) Record the date, time and location of the antigens administered.

Caution: A sudden or severe allergic reaction, such as a rash, hives, or anaphylaxis can occur in people who are highly sensitive. Therefore, the subject should be observed for approximately 20 minutes after injections.

References:

1. Calianno, C. & Ino, T. Getting a Reaction of Anergy Panel Testing. Nursing 95. 1995, 20:58-612.

2. Clyne, K. & Ternes, R. Pharmacy-based skin testing program in a community hospital. Am J Health-Syst Pharm Vol. 53 Sept. 1, 1963.

3. CDC Core Curriculum on Tuberculosis, What the Clinician Should Know: Fourth Edition, 2004.

4. CDC Anergy skin testing and preventative therapy for HIV-infected persons: revised recommendations MMWR 1997; 46 (No.RR-15)

PROCEDURE FOR READING THE DELAYED TYPE HYPERSENSITIVITY (DTH) TEST

NOTE: The results of the skin test must be read by a trained health care worker 48 to 72 hours from the time the test was administered. A reading must also be performed just prior to administration of MDX-1106 if not already subsumed under the 48/72 hour timepoint.

Supplies

- Small, plastic, flexible ruler marked in millimeters
- Ball point pen to mark edges of the induration
- Alcohol pad to clean off pen marks
- Subject's record or other appropriate form for documenting the measurement results

Preparation

- Explain the procedure to the subject to put him/her at ease
- Wash your hands
- Make the subject feel at ease with his/her arm in a relaxed position

Locate the skin-test site

- Inspect the arm in good light and on a firm surface
- Locate the site of injection on the palm-side-up surface of the forearm with the subject's arm supported and slightly flexed at the elbow

Palpate

- The basis of reading the skin test is the presence or absence of induration (hard, dense, raised formation)
- Keep your fingernails short enough so they do not extend beyond the fingertip
- Only the part of the reaction that can be felt, which is the induration, is measured, even if there is soft swelling or redness at the site. Keep in mind that there might not be an induration.
- Since the induration is not always visible, you must rely on palpation with your fingertips to determine induration at the injection site
- Touch the area lightly with the pads of your fingertips
- Using a light, gentle motion, sweep your fingertips in 2-inch diameters from the injection site in all four directions to locate the margins or edges of the induration
- Use a zigzag, feather-like touch to palpate the area for margins of induration. Be careful not to confuse a margin of induration with a margin of muscle on the forearm. To check this, repeat the palpation with the subject's arm raised to a 45-degree angle.

Mark

- The diameter of the induration is measured across two diameters. The mean of the longest and midpoint perpendicular diameters of the indurated area should be reported.

- From 1 inch beyond any palpable induration, apply the tip of the ballpoint pen to the skin holding it a 45° angle and draw a line toward the induration. When a distinct change in rolling resistance is felt, remove the pen tip from the skin. That is one edge of the induration.
- Repeat the steps on the opposite side of the induration.
- For irregular margins of induration, mark and measure the longest diameter across the forearm

Measure

- Measure only the area of induration, a hard, dense, raised formation
- Do not measure erythema, reddening of the skin that can also have swelling, but grade as indicated below.
- Use the millimeter ruler to measure the diameter of the induration perpendicular to the long axis of the forearm
- Measure the distance in millimeters between the ends of the 2 pen marks.
- If the measurement falls between the two divisions on the millimeter scale, record the lower mark.

Record

- Record the exact measurement in millimeters of induration in the subject's record or other appropriate form for documenting the measurement results
- Do not record the interpretation of the results as "positive" or "negative."
- Record the date and time the test was read, the name and signature of the person who read the skin test, and the presence or absence of adverse effects (i.e., blistering, redness, and swelling)
- If there is no induration, this measurement should be recorded as 0 mm of induration

Example

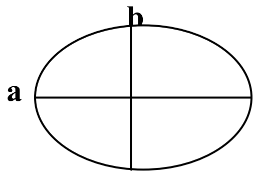

Longest diameter **a** - 10 mm

Perpendicular diameter **b -** 8 mm

10 mm + 8 mm = 18 mm (sum of induration)

18 mm/2 = 9 mm (mean of induration)

Grading of Erythema

0 = Absent

1 = Within limits of induration

2 = Exceeds limits of induration or erythema without induration

3 = Symptomatic (i.e., erythema with pain, itching or vesiculation)

Appendix 3: DMID Adult Toxicity Tables

Division of Microbiology and Infectious Disease (DMID) Adult Toxicity Table

November 2007

Draft

Note: The following toxicity table is a DRAFT and designed to provide general guidance on parameters for monitoring safety in clinical trials. This toxicity table is not comprehensive and should not be applied directly to all trials.

When selecting a toxicity table, the following are some of the items that must be taken into consideration:

- The population being studied
- Does the clinical trial evaluate healthy subjects, subjects with a particular disease or condition?
- The stage of test article development
- Is the clinical trial a Phase I, II, III or IV?
- The type of test article
- Does the clinical trial evaluate a drug, device, vaccine or other biologic agent?
- The prior human and preclinical experience with the test article
- Are there any specific findings that require adjustment of the toxicity table?

Single site clinical trials evaluating healthy subjects should conform to the laboratory normal values at the single site. Multi-center clinical trials should reconcile among their laboratory normal values when evaluating a healthy volunteer population.

ABBREVIATIONS: Abbreviations utilized in the Table:

ULN = Upper Limit of Normal	LLN = Lower Limit of Normal
R_x = Therapy	Req = Required
Mod = Moderate	IV = Intravenous
ADL = Activities of Daily Living	Dec = Decreased

ESTIMATING SEVERITY GRADE

For abnormalities NOT found elsewhere in the Toxicity Tables use the scale below to estimate grade of severity:

**GRADE 1**	**Mild:** Transient or mild discomfort (< 48 hours); no medical intervention/therapy required
**GRADE 2**	**Moderate:** Mild to moderate limitation in activity – some assistance may be needed; no or minimal medical intervention/therapy required
**GRADE 3**	**Severe:** Marked limitation in activity, some assistance usually required; medical intervention/therapy required, hospitalizations possible
**GRADE 4**	**Life-threatening:** Extreme limitation in activity, significant assistance required; significant medical intervention/therapy required, hospitalization or hospice care probable

SERIOUS OR LIFE-THREATENING ADVERSE EVENTS

ANY clinical event deemed by the clinician to be serious or life-threatening should be considered a Grade 4 event. Clinical events considered to be serious or life-threatening include, but are not limited to: seizures, coma, tetany, diabetic ketoacidosis, disseminated intravascular coagulation, diffuse petechiae, paralysis, acute psychosis, severe depression.

COMMENTS REGARDING THE USE OF THESE TABLES

- Standardized and commonly used toxicity tables (Division of AIDS, NCI's Common Toxicity Criteria (CTC), and World Health Organization (WHO)) have been adapted for use by the Division of Microbiology and Infectious Diseases (DMID) and modified to better meet the needs of participants in DMID trials.
- For parameters not included in the following Toxicity Tables, sites should refer to the "Guide For Estimating Severity Grade" located above.
- Criteria are generally grouped by body system.

**HEMATOLOGY**				
	**Grade 1**	**Grade 2**	**Grade 3**	**Grade 4**
Hemoglobin	9.5 – 10.5 gm/dL	8.0 - 9.4gm/dL	6.5 – 7.9 gm/dL	< 6.5 gm/dL
Absolute Neutrophil Count	1000-1500/mm^3	750-999/mm^3	500-749/mm^3	<500/mm^3
Platelets	75,000-99,999/mm^3	50,000-74,999/mm^3	20,000-49,999/mm^3	<20,000/mm^3
WBCs	11,000-13,000/ mm^3	13,000-15,000 /mm^3	15,000-30,000/mm^3	>30,000 or <1,000 /mm^3
% Polymorpho-nuclear Leucocytes + Band Cells	> 80%	90 – 95%	>95%	————————
Abnormal Fibrinogen	Low: 100-200 mg/dL<br><br>High: 400-600 mg/dL	Low: <100 mg/dL<br><br>High: >600 mg/dL	Low: < 50 mg/dL<br><br>----------	Fibrinogen associated with gross bleeding or with disseminated coagulation
Fibrin Split Product	20-40 mcg/mL	41-50 mcg/mL	51-60 mcg/mL	> 60 mcg/mL
Prothrombin Time (PT)	1.01 – 1.25 x ULN	1.26-1.5 x ULN	1.51 -3.0 x ULN	>3 x ULN
Activated Partial Thromboplastin (APPT)	1.01 -1.66 x ULN	1.67 – 2.33 x ULN	2.34 – 3 x ULN	> 3 x ULN
Methemoglobin	5.0 – 9.9 %	10.0 – 14.9 %	15.0 – 19.9%	> 20.0 %

**CHEMISTRIES**				
	**Grade 1**	**Grade 2**	**Grade 3**	**Grade 4**
Hyponatremia	130-135 mEq/L	123-129 mEq/L	116-122 mEq/L	< 116 mEq/L or abnormal sodium *with* mental status changes or seizures
Hypernatremia	146-150 mEq/L	151-157 mEq/L	158-165 mEq/L	> 165 mEq/L or abnormal sodium *with* mental status changes or seizures
Hypokalemia	3.0 – 3.4 mEq/L	2.5 – 2.9 mEq/L	2.0 – 2.4 mEq/L or intensive replacement therapy or hospitalization required	< 2.0 mEq/L or abnormal potassium *with* paresis, ileus or life-threatening arrhythmia

**CHEMISTRIES**				
	**Grade 1**	**Grade 2**	**Grade 3**	**Grade 4**
Hyperkalemia	5.6 – 6.0 mEq/L	6.1 – 6.5 mEq/L	6.6 – 7.0 mEq/l	> 7.0 mEq/L or abnormal potassium *with* life-threatening arrhythmia
Hypoglycemia	55-64 mg/dL	40-54 mg/dL	30-39 mg/dL	<30 mg/dL or abnormal glucose *with* mental status changes or coma
Hyperglycemia (nonfasting and no prior diabetes)	116 – 160 mg/dL	161- 250 mg/dL	251 – 500 mg/dL	> 500 mg/dL or abnormal glucose *with* ketoacidosis or seizures
Hypocalcemia (corrected for albumin)	8.4 – 7.8 mg/dL	7.7 – 7.0 mg/dL	6.9 – 6.1 mg/dL	< 6.1 mg/dL or abnormal calcium *with* life threatening arrhythmia or tetany
Hypercalcemia (correct for albumin)	10.6 – 11.5 mg/dL	11.6 – 12.5 mg/dL	12.6 – 13.5 mg/dL	> 13.5 mg/dL or abnormal calcium *with* life threatening arrhythmia
Hypomagnes-emia	1.4 – 1.2 mEq/L	1.1 – 0.9 mEq/L	0.8 – 0.6 mEq/L	< 0.6 mEq/L or abnormal magnesium *with* life-threatening arrhythmia
Hypophospha-temia	2.0 – 2.4 mg/dL	1.5 -1.9 mg/dL or replacement Rx required	1.0 -1.4 mg/dL intensive therapy or hospitalization required	< 1.0 mg/dL or abnormal phosphate *with* life-threatening arrhythmia
Hyperbilirubin-emia (when accompanied by any increase in other liver function test)	1.1 – <1.25 x ULN	1.25 – <1.5 x ULN	1.5 – 1.75x ULN	> 1.75 x ULN
Hyperbilirubinemia (when other liver function are in the normal range)	1.1 - <1.5 x ULN	1.5 - <2.0 x ULN	2.0 – 3.0 x ULN	> 3.0 x ULN
BUN	1.25 – 2.5 x ULN	2.6 – 5 x ULN	5.1 – 10 x ULN	> 10 x ULN
Hyperuricemia (uric acid)	7.5 – 10.0 mg/dL	10.1 – 12.0 mg/dL	12.1 – 15.0 mg/dL	>15.0 mg/dL
Creatinine	1.1 – 1.5 x ULN	1.6 – 3.0 x ULN	3.1 – 6 x ULN	> 6 x ULN or dialysis required

**ENZYMES**				
	**Grade 1**	**Grade 2**	**Grade 3**	**Grade 4**
AST (SGOT)	1.1 - <2.0 x ULN	2.0 – <3.0 x ULN	3.0 – 8.0 x ULN	> 8 x ULN
ALT (SGPT)	1.1 - <2.0 x ULN	2.0 – <3.0 x ULN	3.0 – 8.0 x ULN	> 8 x ULN
GGT	1.1 - <2.0 x ULN	2.0 – <3.0 x ULN	3.0 – 8.0 x ULN	> 8 x ULN
Alkaline Phosphatase	1.1 - <2.0 x ULN	2.0 – <3.0 x ULN	3.0 – 8.0 x ULN	> 8 x ULN
Amylase	1.1 – 1.5 x ULN	1.6 – 2.0 x ULN	2.1 – 5.0 x ULN	> 5.1 x ULN
Lipase	1.1 – 1.5 x ULN	1.6 – 2.0 x ULN	2.1 – 5.0 x ULN	> 5.1 x ULN

**URINALYSIS**				
	**Grade 1**	**Grade 2**	**Grade 3**	**Grade 4**
Proteinuria	1+ or 200 mg – 1 gm loss/day	2-3+ or 1- 2 gm loss/day	4+ or 2-3.5 gm loss/day	nephrotic syndrome or > 3.5 gm loss/day
Hematuria	microscopic only <10 rbc/hpf	gross, no clots >10 rbc/hpf	gross, with or without clots, OR red blood cell casts	obstructive or required transfusion

**CARDIOVASCULAR**				
	**Grade 1**	**Grade 2**	**Grade 3**	**Grade 4**
Cardiac Rhythm		asymptomatic, transient signs, no Rx required	recurrent/persistent; symptomatic Rx required	unstable dysrythmia; hospitalization and treatment required
Hypertension	transient increase > 20 mm/Hg; no treatment	recurrent, chronic increase >20mm/Hg. /treatment required	acute treatment required; out-subject treatment or hospitalization possible	end organ damage or hospitalization required
Hypotension	transient orthostatic hypo-tension with heart rate increased by <20 beat/min or decreased by <10 mm Hg systolic BP, No treatment required	symptoms due to orthostatic hypotension or BP decreased by <20 mm Hg systolic; correctable with oral fluid treatment	requires i.v. fluids; no hospitalization required	mean arterial pressure <60mm/Hg or end organ damage or shock; requires hospitalization and vasopressor treatment
Pericarditis	minimal effusion	mild/moderate asymptomatic effusion, no treatment	symptomatic effusion; pain; EKG changes	tamponade; pericardiocentesis or surgery required
Hemorrhage, Blood Loss	microscopic/occult	mild, no transfusion	gross blood loss; 1-2 units transfused	massive blood loss; > 3 units transfused

**RESPIRATORY**				
	**Grade 1**	**Grade 2**	**Grade 3**	**Grade 4**
Cough	transient- no treatment	persistent cough; treatment responsive	Paroxysmal cough; uncontrolled with treatment	--------------------
Bronchospasm, Acute	transient; no treatment; 70% - 80% FEV_1 of peak flow	requires treatment; normalizes with bronchodilator; FEV_1 50% - 70% (of peak flow)	no normalization with bronchodilator; FEV_1 25% - 50% of peak flow; or retractions present	cyanosis: FEV_1 < 25% of peak flow or intubation necessary
Dyspnea	dyspnea on exertion	dyspnea with normal activity	dyspnea at rest	dyspnea requiring Oxygen therapy

**GASTROINTESTINAL**				
	**Grade 1**	**Grade 2**	**Grade 3**	**Grade 4**
Nausea	mild or transient; maintains reasonable intake	moderate discomfort; intake decreased significantly; some activity limited	no significant intake; requires IV fluids	hospitalization required;
Vomiting	1 episode in 24 hours	2-5 episodes in 24 hours	>6 episodes in 24 hours or needing IV fluids	physiologic consequences requiring hospitalization or requiring parenteral nutrition
Constipation	requiring stool softener or dietary modification	requiring laxatives	obstipation requiring manual evacuation or enema	obstruction or toxic megacolon
Diarrhea	mild or transient; 3-4 loose stools/day or mild diarrhea last < 1 week	moderate or persistent; 5-7 loose stools/day or diarrhea lasting >1 week	>7 loose stools/day or bloody diarrhea; or orthostatic hypotension or electrolyte imbalance or >2L IV fluids required	hypotensive shock or physiologic consequences requiring hospitalization
Oral Discomfort/ Dysphagia	mild discomfort; no difficulty swallowing	some limits on eating/drinking	eating/talking very limited; unable to swallow solid foods	unable to drink fluids; requires IV fluids

**NEUROLOGICAL**				
	**Grade 1**	**Grade 2**	**Grade 3**	**Grade 4**
Neuro-Cerebellar	slight incoordination dysdiadochokinesis	intention tremor, dysmetria, slurred speech; nystagmus	locomotor ataxia	incapacitated
Psychiatric	mild anxiety or depression	moderate anxiety or depression; therapy required; change in normal routine	severe mood changes requiring therapy; or suicidal ideation; or aggressive ideation	acute psychosis requiring hospitalization; or suicidal gesture/attempt or hallucinations
Muscle Strength	subjective weakness no objective symptoms/ signs	mild objective signs/symptoms no decrease in function	objective weakness function limited	paralysis
Paresthesia (burning, tingling, etc.)	mild discomfort; no treatment required	moderate discomfort; non-narcotic analgesia required	severe discomfort; or narcotic analgesia required with symptomatic improvement	incapacitating; or not responsive to narcotic analgesia
Neuro-sensory	mild impairment in sensation (decreased sensation, e.g., vibratory, pinprick, hot/cold in great toes) in focal area or symmetrical distribution; or change in taste, smell, vision and/or hearing	moderate impairment (mod decreased sensation, e.g., vibratory, pinprick, hot/cold to ankles) and/or joint position or mild impairment that is not symmetrical	severe impairment (decreased or loss of sensation to knees or wrists) or loss of sensation of at least mod degree in multiple different body areas (i.e., upper and lower extremities)	sensory loss involves limbs and trunk; paralysis; or seizures

**MUSCULOSKELETAL**				
	**Grade 1**	**Grade 2**	**Grade 3**	**Grade 4**
Arthralgia (joint pain)	mild pain not interfering with function	moderate pain, analgesics and/or pain interfering with function but not with activities of daily living	severe pain; pain and/or analgesics interfering with activities of daily living	disabling pain
Arthritis	mild pain with inflammation, erythema or joint swelling – but not interfering with function	moderate pain with inflammation, erythema or joint swelling – interfering with function, but not with activities of daily living	severe pain with inflammation, erythema or joint swelling –and interfering with activities of daily living	permanent and/or disabling joint destruction
Myalgia	myalgia with no limitation of activity	muscle tenderness (at other than injection site) or with moderate impairment of activity	severe muscle tenderness with marked impairment of activity	frank myonecrosis

**SKIN**				
	**Grade 1**	**Grade 2**	**Grade 3**	**Grade 4**
Mucocutaneous	erythema; pruritus	diffuse, maculopapular rash, dry desquamation	vesiculation or moist desquamation or ulceration	Exfoliative dermatitis, mucous membrane involvement or erythema, multiforme or suspected Stevens-Johnson or necrosis requiring surgery
Induration	< 15mm	15-30 mm	>30mm	
Erythema	< 15mm	15-30 mm	>30mm	
Edema	< 15mm	15-30 mm	>30mm	
Rash at Injection Site	< 15mm	15-30 mm	>30mm	
Pruritus	slight itching at injection site	moderate itching at injection extremity	itching over entire body	

**SYSTEMIC**				
	**Grade 1**	**Grade 2**	**Grade 3**	**Grade 4**
Allergic Reaction	pruritus without rash	localized urticaria	generalized urticaria; angioedema	anaphylaxis
Headache	mild, no treatment required	transient, moderate; treatment required	severe; responds to initial narcotic therapy	intractable; requires repeated narcotic therapy
Fever: oral	37.7 – 38.5°C or 100.0 – 101.5°F	38.6 – 39.5°C or 101.6 – 102.9°F	39.6 – 40.5°C or 103 – 105°F	> 40°C or > 105°F
Fatigue	normal activity reduced < 48 hours	normal activity decreased 25- 50% > 48 hours	normal activity decreased > 50% can't work	unable to care for self

Appendix 4: Pre-existing Autoimmune Diseases

Subjects should be carefully questioned regarding their history of acquired or congenital immune deficiencies or autoimmune disease. Subjects with any history of immune deficiencies or autoimmune disease are excluded from participating in the study. Possible exceptions to this exclusion could be subjects with a medical history of such entities as atopic disease or childhood arthralgias where the clinical suspicion of autoimmune disease is low. In addition, transient autoimmune manifestations of an acute infectious disease that resolved upon treatment of the infectious agent are not excluded (e.g., acute Lyme arthritis). Please contact the medical monitor regarding any uncertainty over autoimmune exclusions.

Diseases that may be autoimmune related include but are not limited to the following:

Acute disseminated encephalomyelitis (ADEM)
IgA nephropathy
Addison’s disease
Inflammatory bowel disease
Alopecia universalis
Interstitial cystitis
Ankylosing spondylitis
Lambert-Eaton Myasthenia Syndrome
Antiphospholipid antibody syndrome (APS)
Lupus erythematosus
Aplastic anemia
Lyme disease - chronic
Asthma
Meniere’s Syndrome
Autoimmune hemolytic anemia
Mooren’s ulcer
Autoimmune hepatitis
Morphea
Autoimmune hypophysitis
Multiple sclerosis
Autoimmune hypoparathyroidism
Myasthenia gravis
Autoimmune myocarditis
Neuromyotonia
Autoimmune Oophoritis
Opsoclonus myoclonus syndrome (OMS)
Autoimmune orchitis
Optic neuritis
Autoimmune thrombocytopenic purpura
Ord’s thyroiditis
Behcet’s disease
Pemphigus
Bullous pemphigold
Pernicious anemia
Celiac disease
Polyarteritis nodusa
Chronic fatigue syndrome
Polyarthritis
Chronic inflammatory demyelinating polyneuropathy
Polygrandular autoimmune syndrome
Chung-Strauss Syndrome
Primary biliary cirrhosis
Crohn’s disease
Psoriasis
Dermatomyositis
Reiter’s syndrome
Diabetes mellitus type 1
Rheumatoid arthritis
Dysautonomia
Sarcoidosis
Eczema
Scleroderma
Sjögren’s syndrome
Epidermolysis bullosa acquista
Stiff-Person Syndrome
Gestational pemphigoid
Takayasu’s Arteritis
Giant Cell Arteritis
Ulcerative colitis
Goodpasture’s syndrome
Vitiligo
Graves’ disease
Vogt-Kovanagi-Harada disease
Guillain-Barré syndrome (GBS)
Vulvodynia
Hashimoto’s disease
Wegener’s granulomatosis
Kawasaki’s Disease

Appendix 5: List of Immune-Related Adverse Event Symptoms

Subjects should be questioned to elicit information regarding the occurrence of any of the following adverse events, as they may be indicators of immune-related adverse events such as cardiomyopathy, diabetes, thyroid deficiency, adrenal insufficiency, gastritis, lupus, hypersensitivity, or liver toxicity.

**Body System**	**Adverse Event**
Cardiovascular	Chest pain
Cardiovascular	Hypotension
Cardiovascular	Pale or purple fingers or toes from cold or stress (Raynaud's phenomenon)
Eyes	Blurry vision
Gastrointestinal	Abdominal bloating
Gastrointestinal	Abdominal pain
Gastrointestinal	Belching
Gastrointestinal	Black stool or blood in stool
Gastrointestinal	Blood in vomit
Gastrointestinal	Burning feeling in stomach
Gastrointestinal	Constipation
Gastrointestinal	Diarrhea
Gastrointestinal	Feeling of fullness
Gastrointestinal	Foul taste in mouth
Gastrointestinal	Mouth sores
Gastrointestinal	Mucosal pigmentation
Gastrointestinal	Nausea
Gastrointestinal	Stomach cramping
Gastrointestinal	Stomach upset
Gastrointestinal	Vomiting
General	Cold intolerance
General	Dizziness
General	Excessive thirst
General	Extreme hunger
General	Fatigue

**Body System**	**Adverse Event**
General	Fever
General	Hypoglycemia
General	Lethargy
General	Loss of appetite
General	Swelling of the abdomen, legs, ankles, feet, face or around the eyes
General	Swollen glands
General	Weakness
General	Weight gain or increased difficulty losing weight
General	Weight loss
Musculoskeletal	Flu-like symptoms, aching muscles or joint pains.
Musculoskeletal	Painful or swollen joints and muscle pain
Nervous	Memory loss
Psychiatric	Decreased libido
Psychiatric	Depression
Psychiatric	Irritability
Reproductive	Abnormal menstrual cycles
Respiratory	Difficulty breathing
Skin	Blistering of the skin
Skin	Dry, rough pale skin
Skin	Hair loss
Skin	Itching
Skin	Rash
Skin	Sensitivity to the sun
Skin	Coarse, dry hair
Skin	Cutaneous pigmentation
Skin	Jaundice
Urinary	Frequent urination
